# Radical Reactivity of the Biradical [⋅P(μ‐NTer)_2_P⋅] and Isolation of a Persistent Phosphorus‐Cantered Monoradical [⋅P(μ‐NTer)_2_P‐Et]

**DOI:** 10.1002/chem.202200624

**Published:** 2022-05-19

**Authors:** Jan Rosenboom, Lukas Chojetzki, Tim Suhrbier, Jabor Rabeah, Alexander Villinger, Ronald Wustrack, Jonas Bresien, Axel Schulz

**Affiliations:** ^1^ Institut für Chemie, Universität Albert-Einstein-Straße 3a 18059 Rostock Germany; ^2^ Leibniz-Institut für Katalyse e.V. Albert-Einstein-Straße 29a 18059 Rostock Germany

**Keywords:** biradicals, molecular chemistry, persistent radicals, phosphorus chemistry, radical chemistry

## Abstract

The activation of C−Br bonds in various bromoalkanes by the biradical [⋅P(μ‐NTer)_2_P⋅] (**1**) (Ter=2,6‐bis‐(2,4,6‐trimethylphenyl)‐phenyl) is reported, yielding *trans*‐addition products of the type [Br−P(μ‐NTer)_2_P−R] (**2**), so‐called 1,3‐substituted *cyclo*‐1,3‐diphospha‐2,4‐diazanes. This addition reaction, which represents a new easy approach to asymmetrically substituted *cyclo*‐1,3‐diphospha‐2,4‐diazanes, was investigated mechanistically by different spectroscopic methods (NMR, EPR, IR, Raman); the results suggested a stepwise radical reaction mechanism, as evidenced by the in‐situ detection of the phosphorus‐centered monoradical [⋅P(μ‐NTer)_2_P‐R].< To provide further evidence for the radical mechanism, [⋅P(μ‐NTer)_2_P‐Et] (**3Et**⋅) was synthesized directly by reduction of the bromoethane addition product [Br‐P(μ‐NTer)_2_P‐Et] (**2** **a**) with magnesium, resulting in the formation of the persistent phosphorus‐centered monoradical [⋅P(μ‐NTer)_2_P‐Et], which could be isolated and fully characterized, including single‐crystal X‐ray diffraction. Comparison of the EPR spectrum of the radical intermediate in the addition reaction with that of the synthesized new [⋅P(μ‐NTer)_2_P‐Et] radical clearly proves the existence of radicals over the course of the reaction of biradical [⋅P(μ‐NTer)_2_P⋅] (**1**) with bromoethane. Extensive DFT and coupled cluster calculations corroborate the experimental data for a radical mechanism in the reaction of biradical [⋅P(μ‐NTer)_2_P⋅] with EtBr. In the field of hetero‐cyclobutane‐1,3‐diyls, the demonstration of a stepwise radical reaction represents a new aspect and closes the gap between P‐centered biradicals and P‐centered monoradicals in terms of radical reactivity.

## Graphical abstract

**Revealing a radical route**: The radical reactivity of the biradical [⋅P(μ‐NTer)_2_Ṗ] has been investigated. It was possible to synthesize the persistent phosphorus‐centered monoradical [⋅P(μ‐NTer)_2_P−Et] and identify it as a radical intermediate during the addition of bromoethane to [⋅P(μ‐NTer)_2_Ṗ]. A possible stepwise mechanism of this reaction was formulated and is supported by both detailed quantum chemical calculations and experimental results.

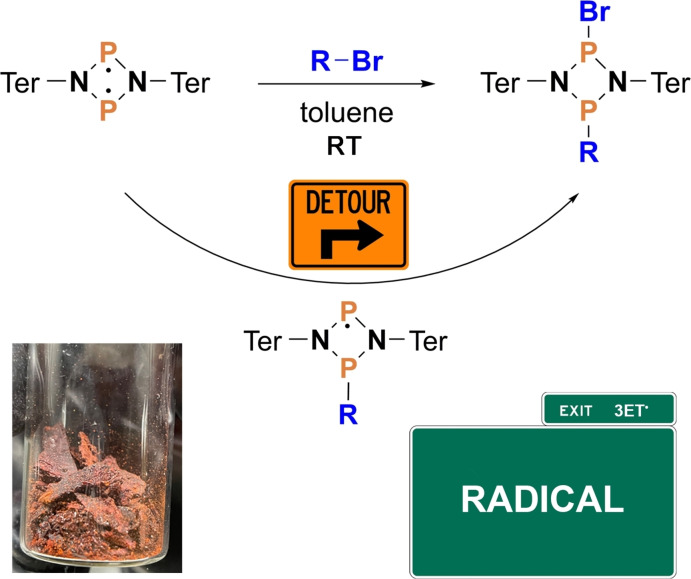

## Introduction

**Scheme 1:**
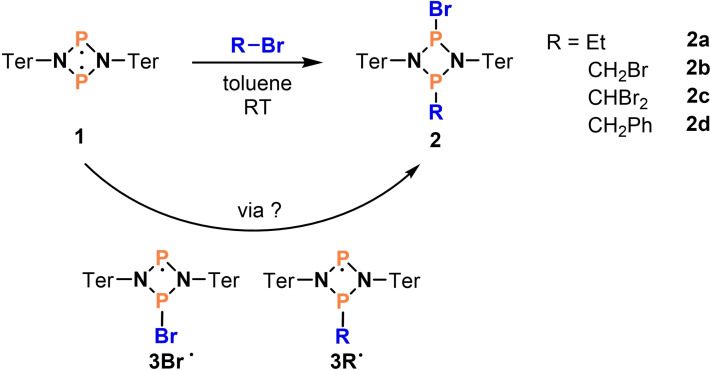
Addition of different bromoalkanes to biradical **1**.

Open‐shell singlet biradical(oid)s, such as [⋅P(μ‐NTer)_2_P⋅] (**1**; Scheme 1) with a biradical character of 25 %,^[1]^ are molecular species with a spin density of exactly zero at any point in space,^[2]^ even though the two radical electrons tend to avoid each other.^[3]^ There are many classifications and names for biradicals (diradical, biradicaloid, etc.);^[4]^ however, throughout this paper we will only use the term biradical since the transitions between open‐shell singlet biradical→biradicaloid→closed‐shell singlet species are smooth in terms of electronic interaction.[^5^, ^6^] Depending on the strength of the antiferromagnetic coupling between the electrons and therefore on the degree of biradical character, singlet biradicals usually feature a reactivity that lies between typical closed‐shell and radical species.[^3^, ^7^, ^8^, ^9^, ^10^, ^11^, ^12^] That is, biradicals can undergo pericyclic, concerted reactions, or step‐wise, radical‐type reactions.

Herein, we report on the activation of C−Br bonds in the reaction of biradical **1** with bromoalkanes featuring a radical mechanism. Phosphorus‐centered biradical **1** that was first synthesized in 2011 (Scheme 1)^[13]^ belongs to the class of hetero‐cyclobutane‐1,3‐diyls, which was made accessible by the Niecke group who published the first stable congener in 1995 ([ClC(μ‐PMes*)]_2_, Mes*=2,4,6‐tri‐*tert*‐butylphenyl).^[14]^ Ever since, various congeneric hetero‐cyclobutanediyls have been discovered,[^15^, ^16^, ^17^, ^18^, ^19^, ^20^, ^21^, ^22^, ^23^, ^24^] and their activation chemistry has been extensively studied.[^15^, ^18^, ^23^, ^24^, ^25^, ^26^, ^27^, ^28^] To our knowledge, there are only few examples of the activation of carbon‐halogen bonds by biradicals in the literature. For example, Sekiguchi and co‐workers were able to activate CCl_4_ with a Si_2_N_2_ biradical resulting in the formation of the chlorinated four‐membered ring.^[19]^ Bertrand and co‐workers achieved the activation of CCl_3_Br with a P_2_B_2_ biradical.^[29]^

**Scheme 2:**
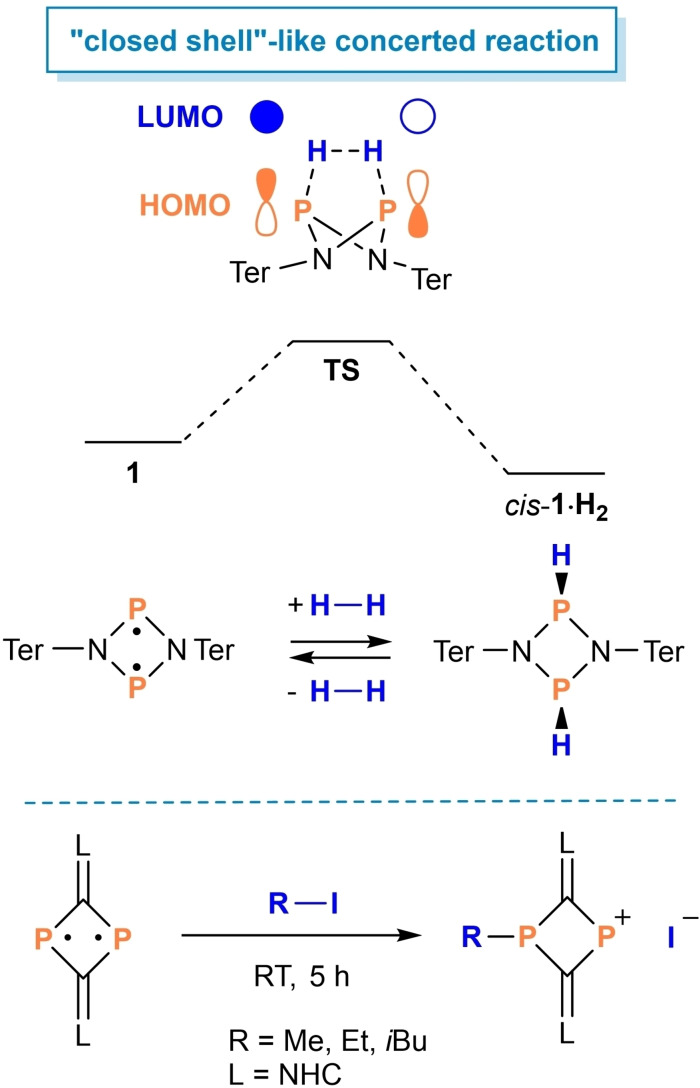
Addition of different bromoalkanes to biradical **1**. Top: reversible addition of H_2_ to **1**.[^2^, ^35^] Bottom: activation of alkyl iodides with a C_2_P_2_ biradical.^[30]^

Very recently the groups of Zu and Li reported the activation of alkyl iodides by a NHC‐stabilized C_2_P_2_ biradical forming an ion pair (Scheme 2), bottom, NHC=N‐heterocyclic carbene).^[30]^ However, to the best of our knowledge, the mechanism of the activation chemistry of these hetero‐cyclobutanediyls with respect to radical behavior has not yet been investigated. This is because many biradical reactions follow a classical “closed‐shell”‐like concerted reaction path (Scheme 2). For example, we could show that **1** is capable of activating small molecules bearing single (H_2_, chalkogenes) and multiple bonds (alkenes, alkynes, isonitriles).[^13^, ^31^, ^32^, ^33^] Mechanistically, these reactions mostly represent concerted [2+2] additions or insertion reactions (i. e., typical “closed‐shell” reactivity).^[34]^ Radical reactivity of **1** (i. e., stepwise addition reactions), which would close the gap between P‐centered biradicals and P‐centered monoradicals, has not been described so far.

## Results and Discussion

### Bromoalkane addition: Synthesis of 1,3‐substituted cyclo‐1,3‐diphospha‐2,4‐diazanes

**Figure 1:**
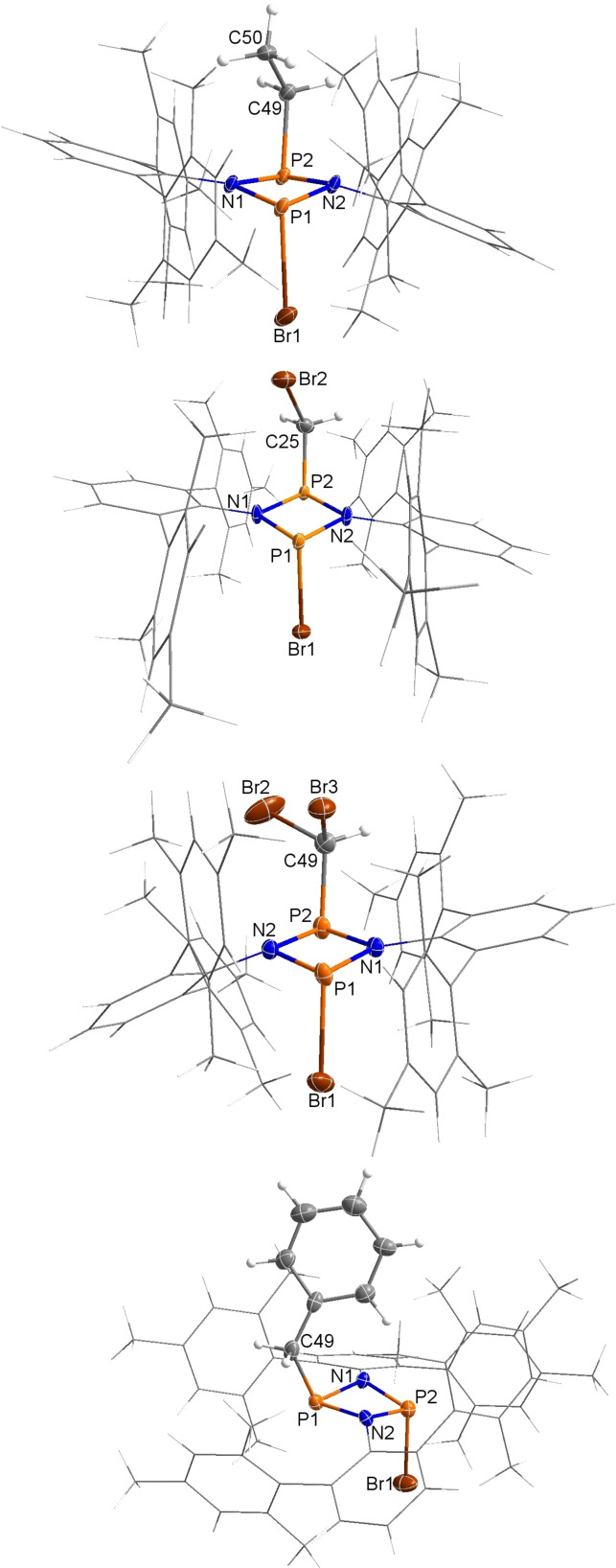
Molecular structure of bromoalkane products **2** in the crystal (from top to the bottom: **2** **a**, **2** **b**, **2** **c**, and **2** **d**). Ellipsoids set at 50 % probability (123 K). Selected bond lengths [Å] and dihedral angles [°] are listed in Table S4 in the Supporting Information.

We started this project with addition reactions between biradical **1** and a series of bromoalkanes (Scheme 1), affording addition products **2**, so‐called 1,3‐substituted cyclo‐1,3‐diphospha‐2,4‐diazanes. Over the course of the addition, the C−Br bond of the bromoalkane is cleaved and the bromine atom is attached to one phosphorus atom and the organic substituent to the other. The dominant product of the reaction was always the *trans*‐addition product (Figure 1).

**Figure 2:**
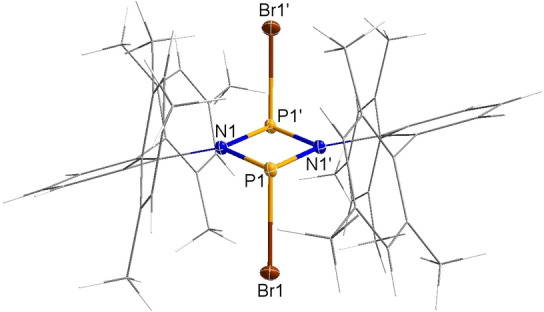
Molecular structure of 1,3‐dibromo‐cyclo‐1,3‐diphospha‐2,4‐diazane **4** in the crystal. Ellipsoids set at 50 % probability (123 K). Selected bond lengths [Å] and dihedral angles [°]: N1−P1 1.730(2), N1−P1’ 1.726(2), P1−Br1 2.311 (1), N1−P1’−P1−N1’ −180.0(1).

The reactions were carried out in toluene at ambient temperature and gave the products in good yields (between 65–90 %). Using ^31^P NMR spectroscopy, the addition reactions could be easily traced as the characteristic singlet of biradical **1** (*δ*[^31^P]=276 ppm)^[13]^ disappeared while two signals appeared in the 195–278 ppm region for the products **2** (**2** **a**: 229/255; **2** **b**: 195/251, **2** **c**: 210/278, and **2** **d**: 242/273 ppm). Interestingly, only the *trans*‐isomers of **2** and always small traces of the dibrominated species *trans*‐[Br‐P(μ‐NTer)_2_P‐Br] (**4**, 278 ppm) could be characterized (Figure 2), hinting at a radical mechanism of the reaction (see below).

1,3‐Dibromo‐*cyclo*‐diphosphadiazane **4** could also be synthesized directly by reacting **1** with dry bromine in benzene at ambient temperature, however, yielding a mixture of the *cis‐* and *trans*‐isomer in a ratio of 2 : 7 (δ[^31^P]=243.9 for *cis*‐ and 277.9 ppm for *trans*‐**4**). After crystallization, almost pure *trans*‐**4** (97 % *trans* isomer) was obtained. **4** has not been reported before and completes the series of 1,3‐dihalogen‐*cyclo*‐1,3‐diphospha‐2,4‐diazanes (formal dihalogen addition products of **1**).^[36—^, ^38]^ ^31^P NMR shifts of all dihalogen addition products are compiled in Table 1. The ^31^P NMR shift increases towards heavier halogens, so the data of **4** fits in with the data of the other adducts. The molecular structure of **4** as determined by single crystal X‐ray diffraction is shown in Figure 2. It reveals a planar N_2_P_2_ ring system and typical P−Br single bond lengths (2.311(1) Å).

**Table 1:** ^31^P NMR shifts (in ppm) of halogen addition products *trans*‐ and *cis*‐[XP(μ‐NTer)_2_PX] (X=F‐I, all recorded in C_6_D_6_).

	*cis*	*trans*
[FP(μ‐NTer)_2_PF]^[36]^	202.9	249.8
[ClP(μ‐NTer)_2_PCl]^[37]^	227.4	264.1
[BrP(μ‐NTer)_2_PBr] (**4**)	243.9	277.9
[IP(μ‐NTer)_2_PI]^[38]^	267.3	296.7

All addition products **2** (see above) could be crystallized and fully characterized (see the Supporting Information). After repeated recrystallization the bromoalkane adducts **2** still contained small impurities, mainly *trans*‐**4**. Corresponding NMR spectra can be found in the Supporting Information. As depicted in Figure 1, the molecular structures were determined by single‐crystal X‐ray diffraction and reveal slightly puckered N_2_P_2_ ring systems (in contrast to **4**) with somewhat shortened N−P single bonds (1.70–1.78 Å, cf. Σ*r*_cov_(P−N)=1.82 Å^[39]^). NBO[^40^, ^41^, ^42^, ^43^] analysis revealed substantial bond polarization (N: 77 % valence electron density, P: 23 %; data for **2** **a**), indicating polar covalent N−P bonding. The newly formed P−C bonds (1.81–1.88 Å) are in the typical range of P−C single bonds (Σ*r*_cov_(P−C)=1.86 Å),^[39]^ while the P−Br bonds (2.33‐2.43 Å) are somewhat elongated in comparison to the sum of the covalent radii Σ*r*_cov_(P−Br)=2.25 Å).^[39]^

1,3‐Substituted *cyclo*‐1,3‐diphospha‐2,4‐diazanes have been known for many decades,[^44^, ^45^] however, almost all synthesis routes lead to symmetrically substituted species.[^46^, ^47^, ^48^, ^49^, ^50^, ^51^, ^52^, ^53^, ^54^, ^55^, ^56^, ^57^, ^58^, ^59^, ^60^] Therefore, the biradical route described here is an elegant alternative, which can be used to generate asymmetrically substituted *cyclo*‐1,3‐diphospha‐2,4‐diazanes (such as **2** **a**–**d**).

### Mechanistic studies for the reaction of biradical 1 with EtBr

**Scheme 3:**
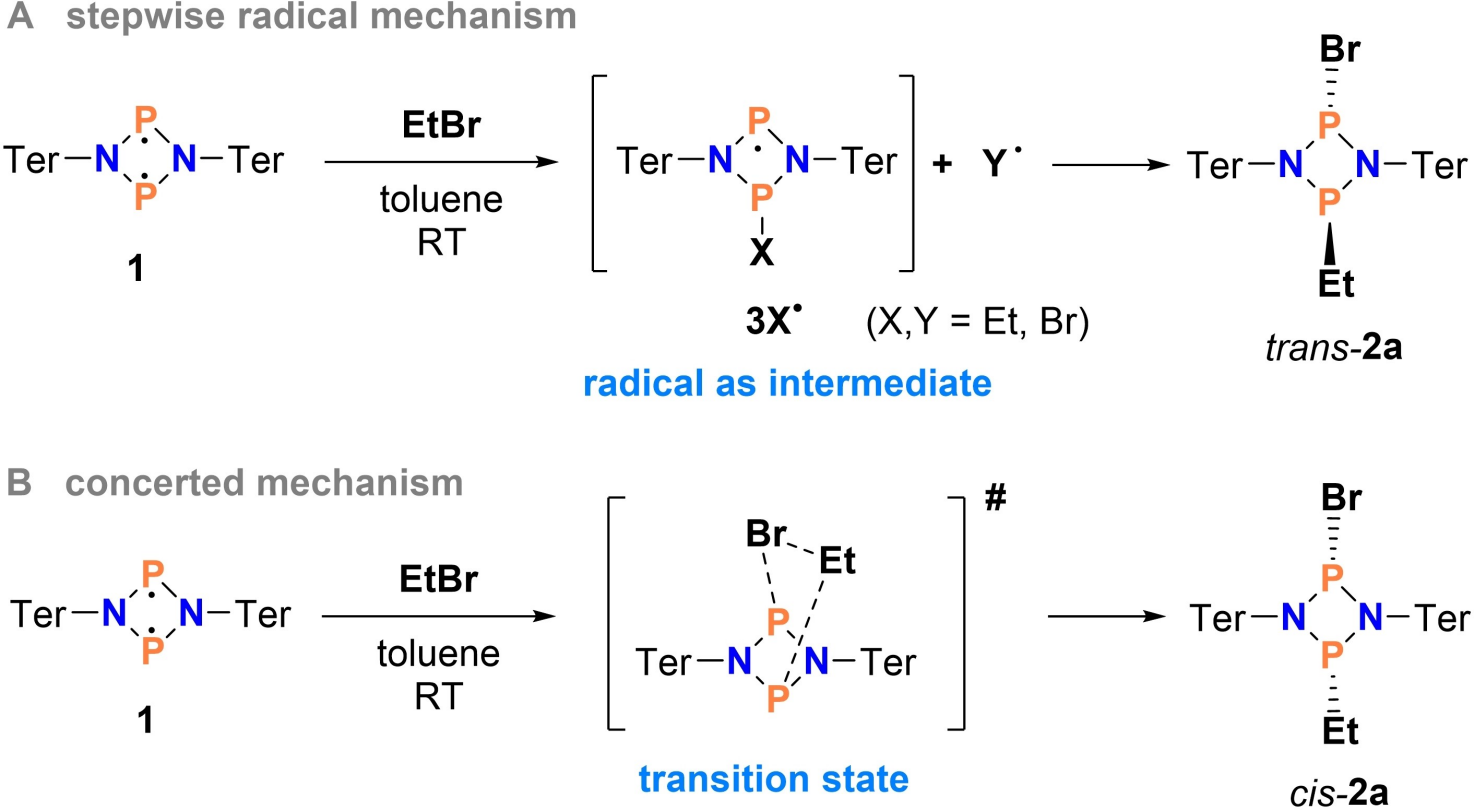
Two conceivable reaction mechanisms for the reaction of biradical **1** with EtBr.

Although only small amounts of 1,3‐dibromo‐*cyclo‐*1,3*‐*diphosphadiazane **4** were found in all reactions of **1** with bromoalkanes, this observation prompted us to further investigate the mechanism of the formation of 1,3‐substituted *cyclo*‐1,3‐diphospha‐2,4‐diazanes (**2**), since only a radical mechanism (Scheme 3) should allow the formation of **4**. Moreover, concerted addition of R−Br to **1** should always give the *cis*‐products of **2**, but we observed only the *trans*‐products. To answer these questions, a series of kinetic studies were performed along with EPR studies for the addition reaction of EtBr giving **2** **a**.

**Figure 3:**
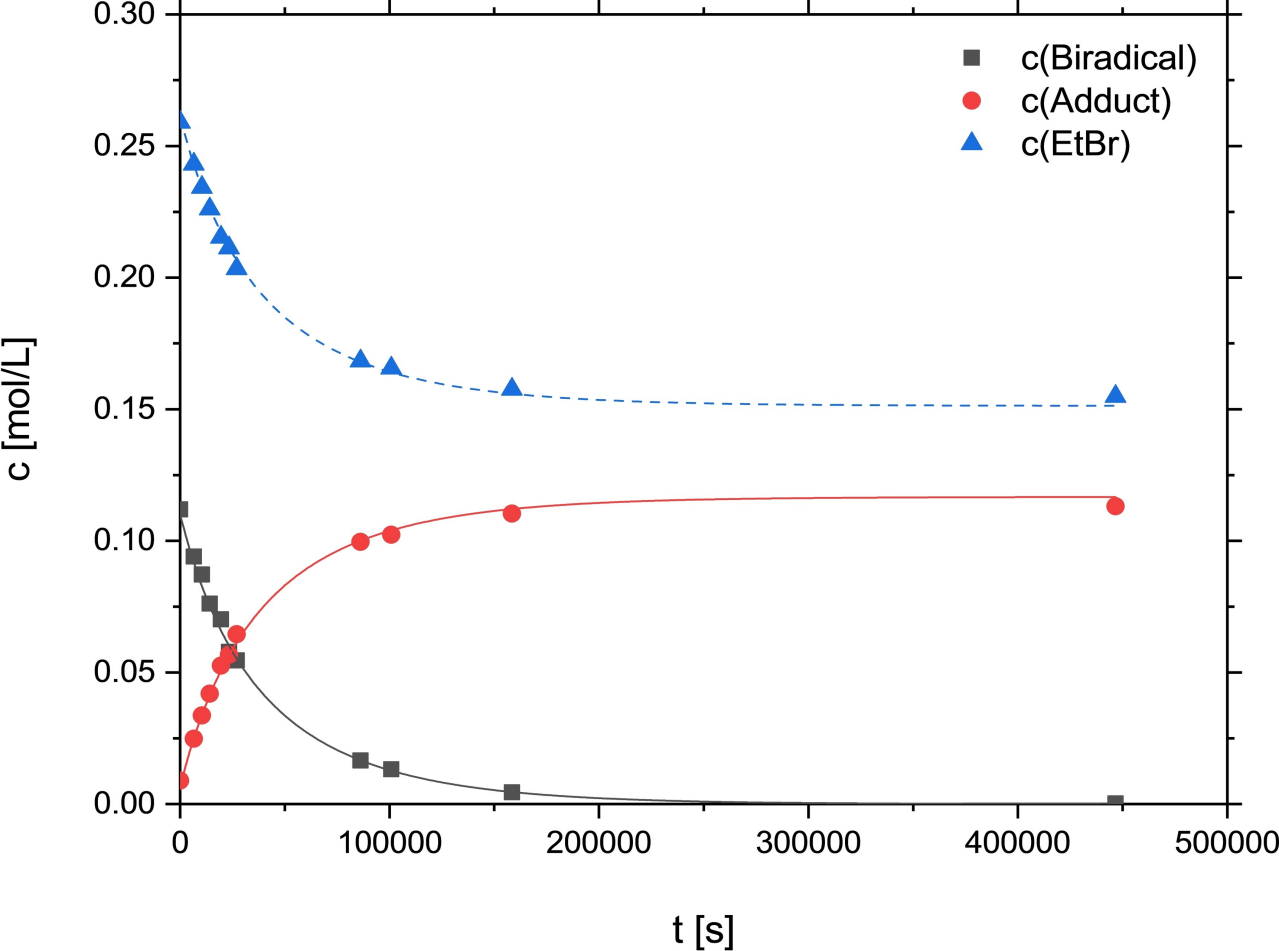
Kinetics of the reaction modeled to ^31^P NMR spectroscopic data as second order kinetics with [Biradical, **1**]_0_=0.110(1), [EtBr]_0_=0.261(1), [Adduct, **2** **a**]_0_=0.007(1) mol L^−1^, and *k*=1.11(3) ×10^−4^ L mol^−1^ s^−1^).

First, the reaction of **1** with EtBr was traced by ^31^P NMR spectroscopy. Spectra were recorded over several days in shorter intervals at the beginning and longer ones towards the end of the experiment. The reaction rate was determined by modeling second order kinetics to the experimental data as illustrated in Figure 3. In particular, the in situ NMR spectra (see the Supporting Information) display the appearance and disappearance of several small signals, including the above‐mentioned by‐product [BrP(μ‐NTer)_2_PBr] (**4**). Thus, the by‐products were attributed to typical chain‐termination reactions (see also Computational Studies below).

Considering the evidence for a radical mechanism, the reaction of EtBr with **1** was repeated in the presence of azobis(isobutyronitrile) (AIBN; Figure S12). To compare the outcomes of the addition reaction with and without radical starter, two batches of **2** **a** were prepared, one with and one without the use of AIBN, but otherwise under exactly the same conditions. A reaction temperature of 60 °C was chosen to activate AIBN. After 2 h, ^31^P NMR spectra were recorded. The ratio of biradical **1** (starting material) to addition product **2** **a** was 2 : 7 with AIBN and 2 : 3 without AIBN (Figure S12), indicating a significant acceleration (x2.5) of the addition reaction upon addition of AIBN. Only a radical reaction mechanism can explain this acceleration.

**Figure 4:**
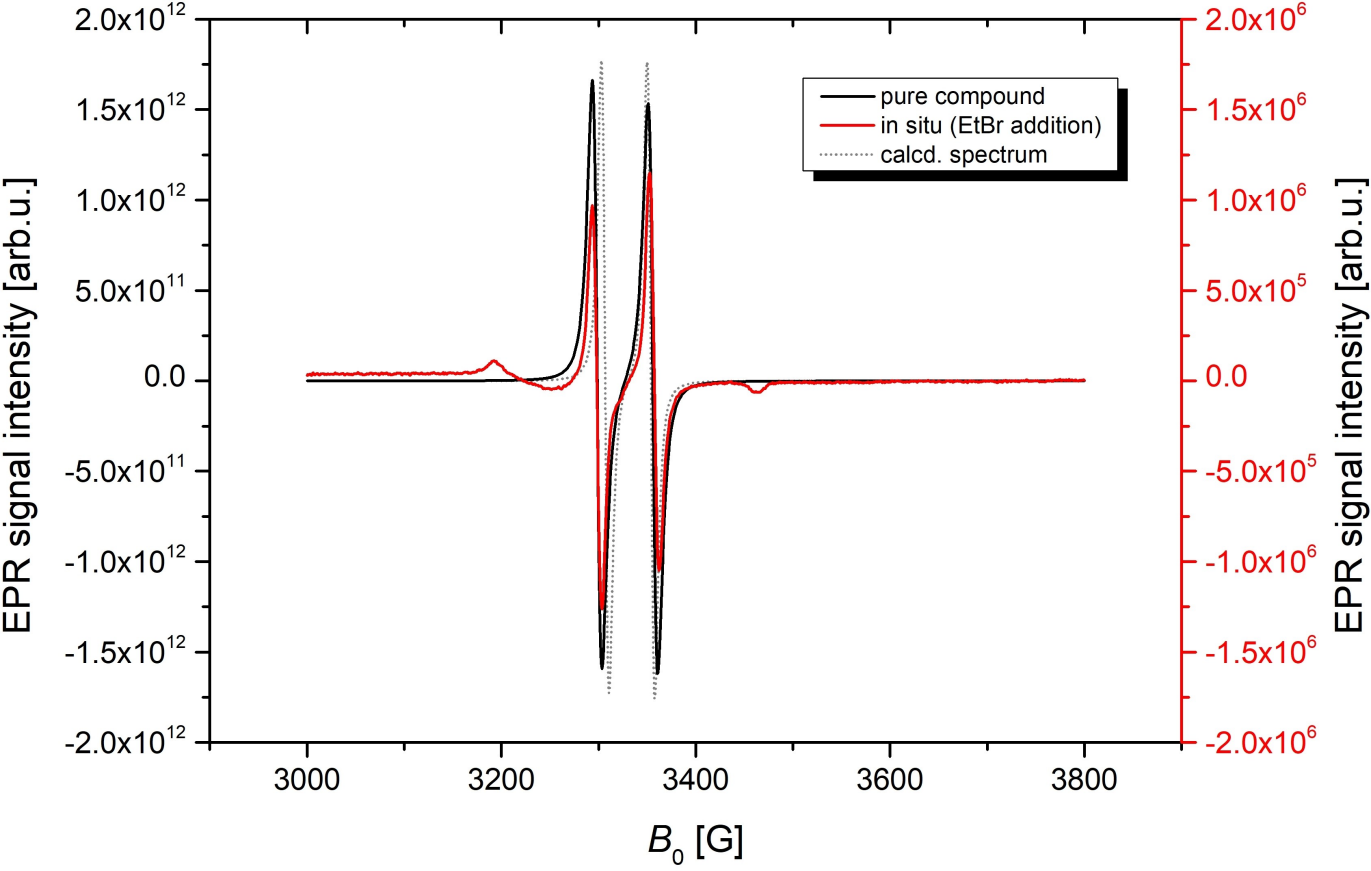
EPR spectra of **3Et**⋅, pure compound (black), in situ during EtBr addition (red), in silico (gray)[^61^, ^62^, ^63^, ^64^, ^65^, ^66^, ^67^] at ambient temperature. The small “side bands” in the red spectrum can be attributed to impurities (note the different scale of the red and black spectra) as they did not change in intensity over time and were present even after the completion of the reaction.

To finally prove that the addition of EtBr is indeed a radical reaction, in situ EPR spectra (red graph in Figure 4) were recorded during the reaction. The spectrum displays a doublet (*g*=2.003) with a large hyperfine coupling constant (*a*(^31^P)=59 G) due to the coupling of an unpaired electron with a phosphorus nucleus. This and the disappearance of the signal once the reaction is completed suggests the presence of a phosphorus‐centered radical intermediate during the reaction.

To elucidate the identity of this intermediate radical, we tried to generate both the bromine‐substituted radical **3Br**⋅ and the Et‐substituted radical **3Et**⋅ directly (Scheme 3). When the dibromine adduct **4** ([BrP(μ‐NTer)_2_PBr]) is treated with magnesium, always biradical **1** ([⋅P(μ‐NTer)_2_P⋅]) is formed, while the reduction of the EtBr adduct **2** **a** leads to the persistent radical **3Et**⋅ that can be isolated in substance (see below). The recorded EPR spectrum of **3Et**⋅ is virtually identical to the spectrum taken from the reaction solution of **1**+EtBr (Figure 4), so that we have a direct proof that **3Et**⋅ forms in situ as intermediate.

Also, DFT computations (see the Supporting Information for further details) indicated that the phosphorus‐centered radical intermediate is the ethyl substituted radical **3Et**⋅ (Scheme 3). The EPR parameters calculated for **3Et**⋅ in silico are in good agreement with the experimental data (gray graph in Figure 4). Additionally, calculations on the other possible, bromine‐substituted radical intermediate **3Br**⋅ hint at a different, more complicated EPR coupling pattern, not at all in alignment with the experimental data.

**Figure 5:**
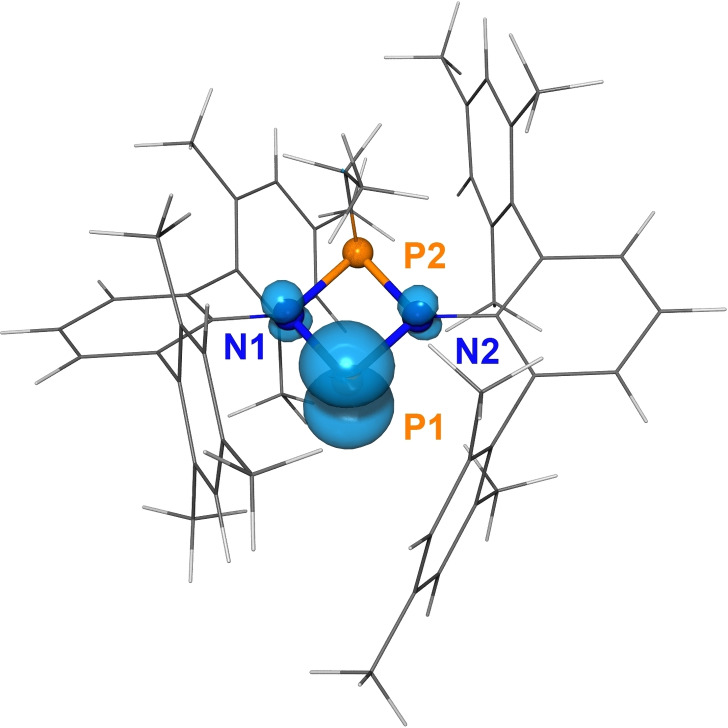
Calculated spin density distribution of **3Et**⋅ (PBE‐D3/def2‐TZVP, iso value=0.008).[^68^, ^69^, ^70^, ^71^, ^72^]

Spin density computations of **3Et**⋅ also confirm it as a phosphorus‐centered radical. The Mulliken spin density is mainly localized at P1 (0.70); while only small values are computed for N1 (0.043) and N2 (0.045), respectively (Figure 5).

### Synthesis and characterization of 1‐ethyl‐1,3‐diphospha‐2,4‐diaza‐3‐yl (3Et⋅)

**Scheme 4:**
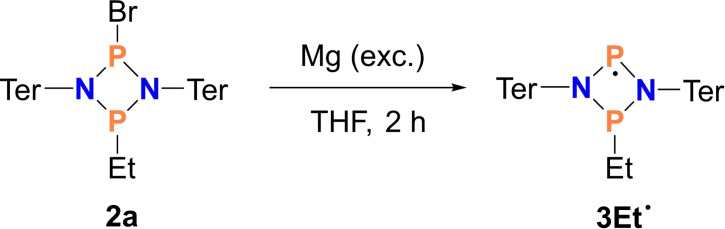
Synthesis of **3Et**⋅.

**Figure 6:**
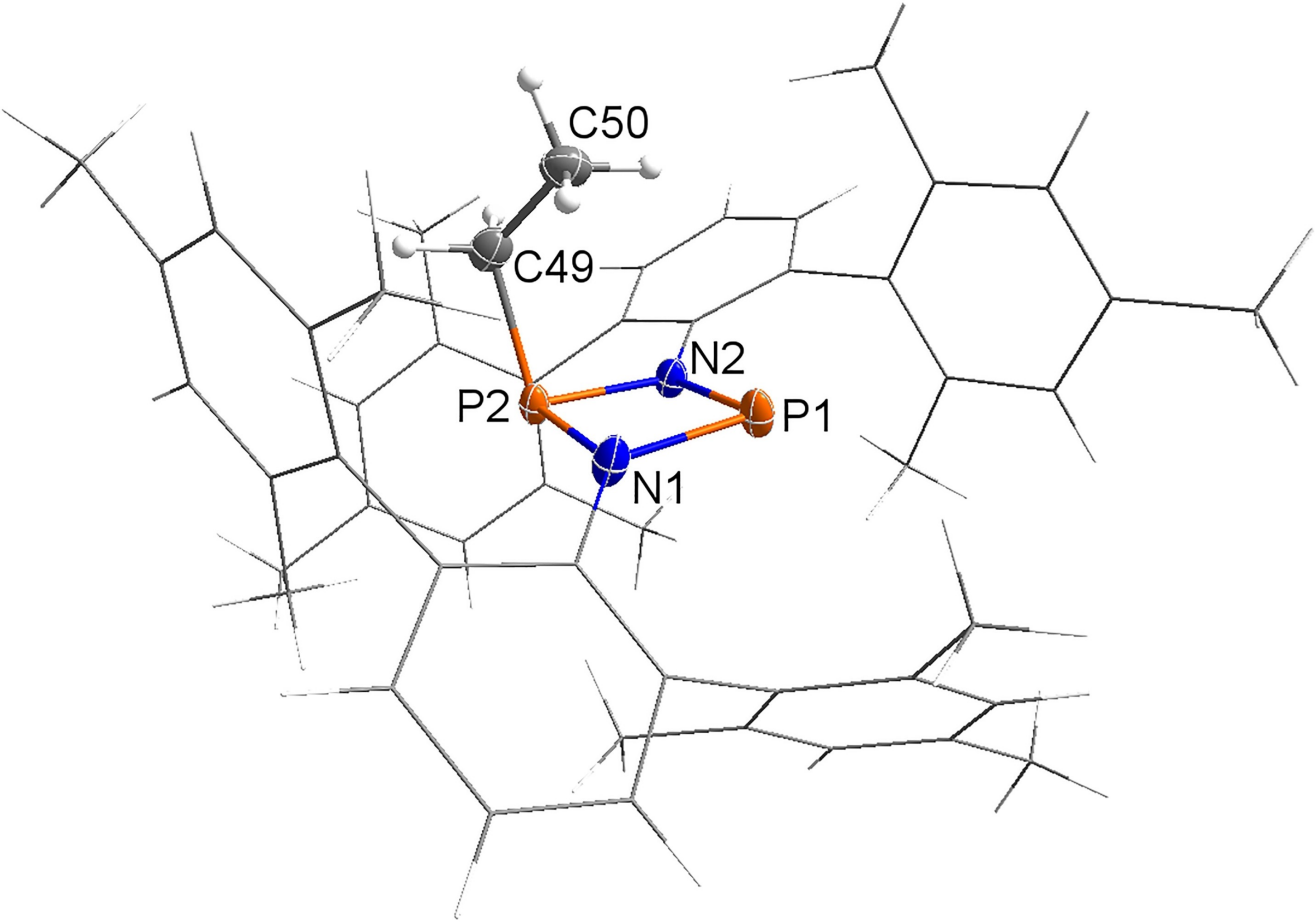
Molecular structure of **3Et**⋅ in the crystal. Ellipsoids set at 50 % probability (123 K). Selected bond lengths [Å] and dihedral angles [°]: N1−P1 1.743(3), N1−P2 1.741(3), N2−P2 1.761(4), N2−P1 1.730(4), P2−C49 1.846(2), N1−P2−P1−N2 174.7(3).

As discussed above, the radical **3Et**⋅ was synthesized purposely by reducing **2** **a** with an excess of magnesium in THF (Scheme 4), by analogy with the reduction of [ClP(μ‐NTer)_2_PCl] to form the biradical **1**.^[73]^ Indeed, the reduction with magnesium worked nicely and the P−Br bond was reductively cleaved within 2 h, yielding after recrystallization the desired, dark red, persistent phosphorus‐centered radical **3Et**⋅ in good yields (83 %). The presence of the molecular radical **3Et**⋅ in the solid state was unequivocally proven by single crystal X‐ray diffraction (Figure 6). Surprisingly, the oxygen‐ and moisture‐sensitive radical **3Et**⋅ is stable for long periods as a solid when stored sealed in an ampoule under inert gas. Thermally, decomposition only begins above its melting point of 195 °C. These properties, together with its good solubility in many organic solvents, allowed a full characterization (see the Supporting Information) and suggest an interesting follow‐up chemistry for radical **3Et**⋅.

Red crystals of **3Et**⋅ crystallized in the monoclinic space group *P*2_1_/*n* with four formula units per cell. As depicted in Figure 6, the molecular radical sits well protected in a pocket formed by the terphenyl substituents attached to both N atoms. The ethyl substituent adopts an *endo* position, which is thermodynamically slightly favored over the *exo*‐species by 9.2 kJ mol^−1^ (level of theory: DLPNO‐CCSD(T)/def2‐TZVP//PBE−D3/def2‐TZVP, see the Supporting Information), and the four‐membered N_2_P_2_ ring system is almost planar (∢(N1‐P2‐P1‐N2)=174.7(3)°). The P−N bond lengths range between 1.730(4) Å (P1−N2) and 1.761(4) Å (P2−N2) and are shortened compared to the sum of covalent radii for a P−N single bond (Σ*r*_cov_(P−N)=1.82 Å),^[39]^ thus suggesting polarized covalent N−P bonds. This is, however, less pronounced than in the starting material **2** **a** (see above).

EPR spectra of a solution of a crystalline sample of **3Et**⋅ (black graph in Figure 4) are virtually identical with the in situ EPR signal during the formation of **2** **a** in the reaction of **1** with EtBr (small “side bands” in the in situ spectrum are attributed to impurities as they did not change over time, see above). Thus, we conclude that **3Et**⋅ is in fact an intermediate in the addition of EtBr to **1**. The measured EPR parameters (*g*=2.003, A=59 G) are in alignment with other literature‐known P‐centered radicals.^[74]^ Also note the different scales of the two measurements depicted in Figure 4 with an intensity difference of six orders of magnitude, showing the difference in concentration between a sample of the pure compound and the in situ measurement.

The UV‐vis spectrum of **3Et**⋅ shows a distinct absorption band at 399 nm (Figure S8), responsible for the dark red color of the compound. DFT calculations indicate that the red color is caused by a combination of excitations, in particular the excitation of the unpaired electron at P1 into a π* orbital of the Ter substituents (see the Supporting Information for TD‐DFT data and NTO transformation, Figures S23 and S24).

**Scheme 5:**
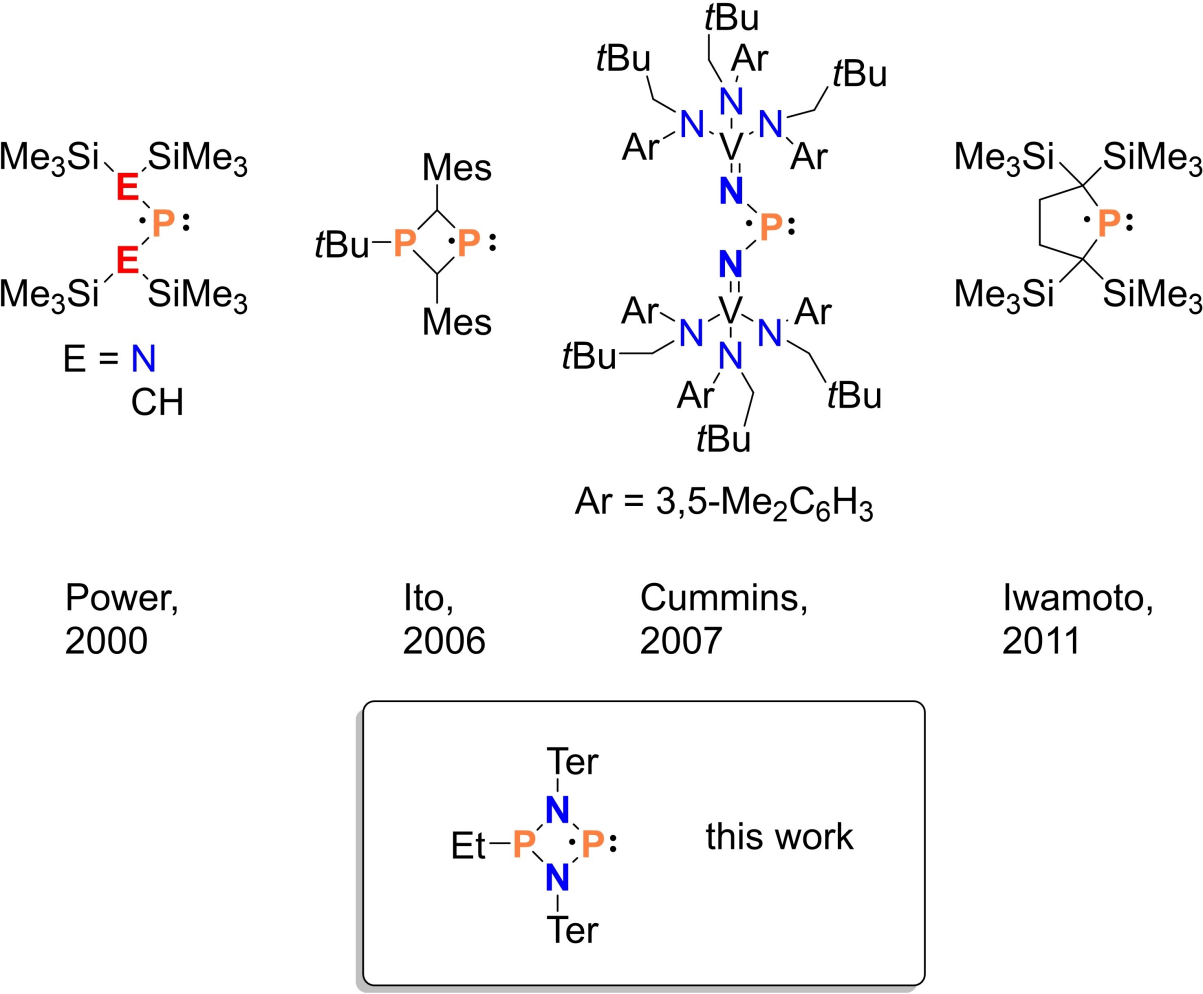
Selected examples of neutral, persistent P‐centered radicals.[^74^, ^83^, ^84^, ^85^]

Persistent radical^[75]^ **3Et**⋅ belongs to the class of P‐centered radicals (Scheme 5). Since the discovery of the first persistent radical (triphenylmethyl) by Gomberg in 1900,^[76]^ considerable advances have been made in persistent radical chemistry and radicals such as TEMPO (2,2,6,6‐tetramethylpiperidin‐1‐yl)oxyl) have found widespread applications in organic chemistry.^[77]^ However, only very few phosphorus‐centered persistent radicals that can be isolated as a solid are known. An overview with examples of neutral phosphorus radicals that could be structurally characterized is presented in Scheme 5. Phosphorus‐centered radicals are known to activate various small molecules, such as P_4,_ [^78^, ^79^] chalcogens_,_[^79^, ^80^] CS_2_ and CO_2._ ^[81]^ Thus, persistent P‐centered radicals are a worthy synthetic target for the investigation of small‐molecule activation.[^74^, ^75^, ^82^, ^83^, ^84^, ^85^, ^86^, ^87^, ^88^, ^89^, ^90^]

Finally, with the ethyl‐substituted radical **3Et**⋅ in hand, we studied the reaction of **3Et**⋅ with EtBr as well as Br_2_ (see Sections 4.3/4.4 in the Supporting Information). Whereas the reaction with bromine led to the formation of *trans*‐**2** **a** and oxidation products, the stoichiometric reaction with EtBr resulted in the formation of a 1 : 1 mixture of *trans*‐**2** **a** and [EtP(μ‐NTer)_2_PEt] (**5** **a**) as shown by ^31^P NMR studies (*trans*‐**2** **a**: 229.2/255.2 ppm; *cis/trans*‐**5** **a**: 225.2/266.7 ppm). The formation of *trans*‐**2** **a** is consistent with the result of the reaction of biradical **1** with EtBr, where only *trans*‐**2** **a** was obtained, but not **5** **a**. Still, the formation of **5** **a** in the reaction of **3Et**⋅ with EtBr is not surprising, since in this instance there is a very large concentration of the **3Et**⋅ radical in solution at the beginning of the reaction, which can react directly with another Et⋅ radical that is formed upon reaction of EtBr with **3Et**⋅ (see reaction 4 in Scheme 7, below), finally yielding [EtP(μ‐NTer)_2_PEt] (**5** **a**). In contrast, the formation of **5** **a** is very unlikely in the reaction of biradical **1** with EtBr, as **3Et**⋅ is only formed as a low‐concentrated intermediate in this case. From these combined experimental (and theoretical) studies, we can summarize that in the reaction of biradical **1** with EtBr, persistent radical **3Et**⋅ occurs as an intermediate, indicating a radical mechanism for the addition reaction.

**Scheme 6:**
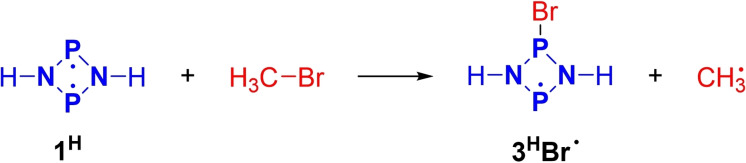
Initial reaction step in the model reaction of **1^H^** with MeBr.

**Scheme 7:**
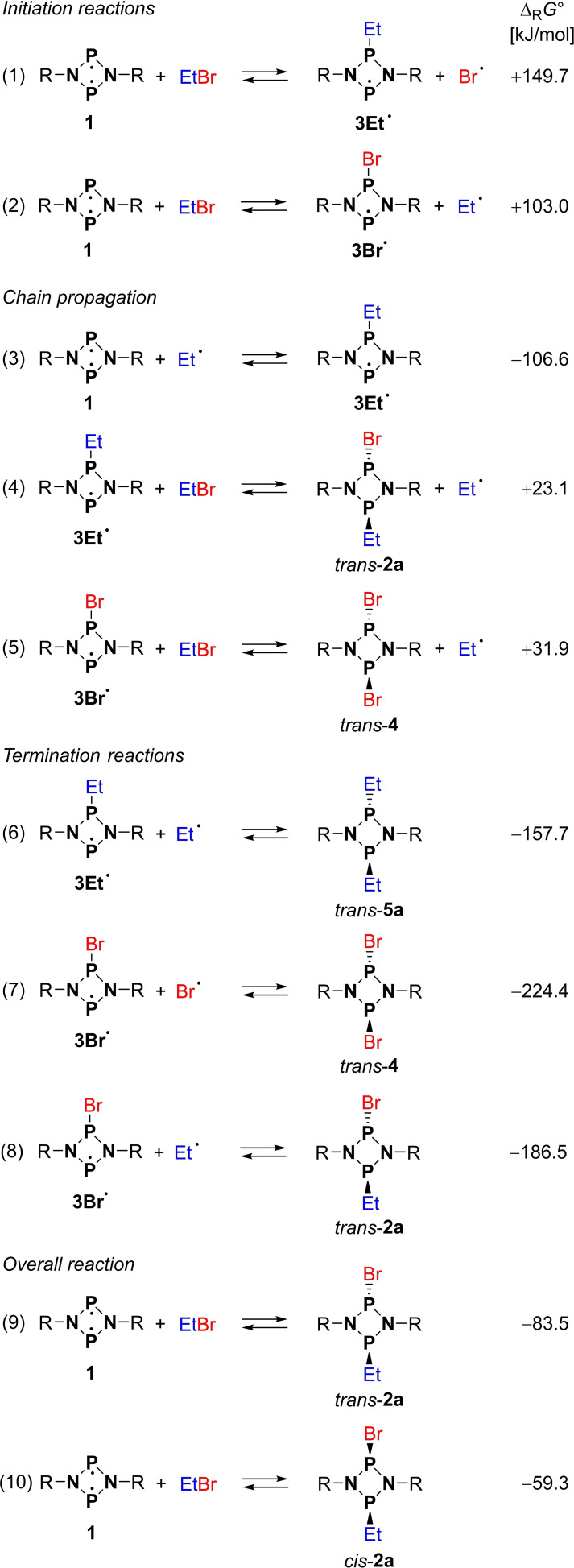
Selected computed radical reaction steps, DLPNO‐CCSD(T)/def2‐TZVP//PBE−D3/def2‐TZVP level of theory. Only the most likely reactions (based on Δ_R_ *G*° and concentrations) in each group are listed. See also Table S6.

#### Computational studies

From the experimental studies on the mechanism of the EtBr addition to biradical **1**, several questions arose, such as why is the radical mechanism preferred over a concerted mechanism (Scheme 3) or which radicals form preferentially? To answer these questions, DFT and coupled cluster calculations on kinetics and thermodynamics were performed (see the Supporting Information for details). Furthermore, to account for the open‐shell biradical character of biradical **1** along the reaction pathway, Complete Active Space SCF (CASSCF) methods were applied. The orbitals of the active space and their contributions are listed in Table S5. To account for dynamic correlation, the CASSCF reference wavefunctions were subjected to multireference perturbation calculations, using the Fully Internally Contracted N‐Electron Valence State Perturbation Theory (FIC‐NEVPT2).[^91^, ^92^, ^93^]

*Model system*. To obtain an initial idea of possible reaction pathways, the model reaction of [⋅P(μ‐NH)_2_P⋅] (**1^H^**) with MeBr was investigated. Therefore, a variety of nudged elastic band (NEB)[^94^, ^95^, ^96^, ^97^, ^98^] and relaxed potential energy surface (PES) scans were performed at the UPBE−D3/def2‐SVP^[99]^ level of theory using ORCA 4.2.1[^61^, ^67^] or Gaussian 09,^[68]^ respectively. Different orientations of the starting materials and configurations of the product were considered. We could not locate a transition state for a concerted mechanism (e. g., analogous to the addition of H_2_ to the singlet biradical [⋅P(μ‐NTer)_2_P⋅], Scheme 2).[^35^, ^100^, ^101^] All results pointed towards a stepwise (i. e., radical) mechanism of the reaction, in agreement with experimental observations. In particular, our model computations implied that the formal abstraction of a Br⋅ radical from the bromoalkane by the singlet biradical initiated the radical chain reaction (see also section “Real system” below). It was therefore of special interest to investigate this first reaction step in more detail (Scheme 6).

**Figure 7:**
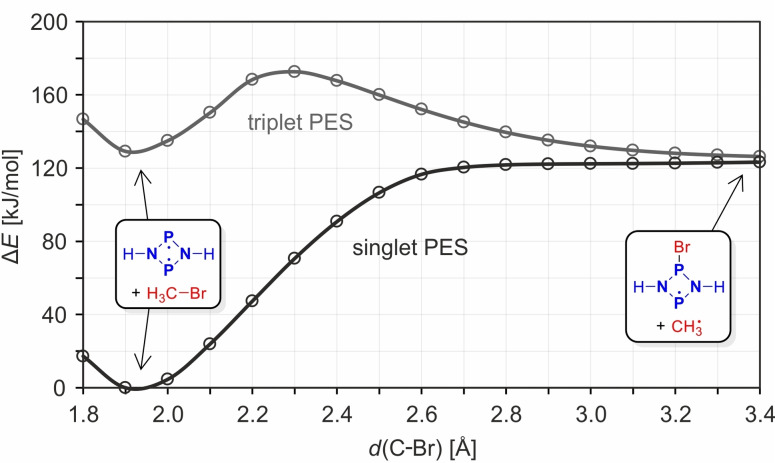
MEP of the reaction [⋅P(μ‐NH)_2_P⋅]+MeBr→[⋅P(μ‐NH)_2_PBr]+Me⋅ on the singlet (S_0_, blue) and triplet (T_1_, red) PES at the FIC‐NEVPT2/def2‐TZVP//UPBE‐D3/def2‐TZVP level of theory. The near degeneracy of singlet and triplet at the end of the reaction indicates two separate radicals.

**Figure 8:**
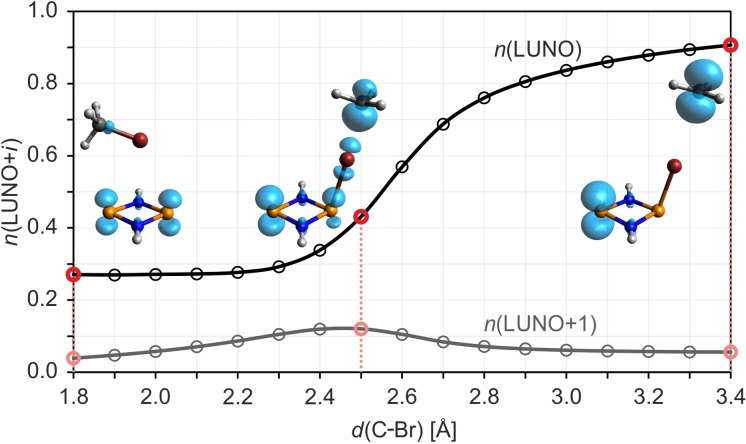
Change in the biradical character (LUNO occupancy; LUNO=lowest unoccupied natural orbital) and tetraradical character (LUNO+1 occupancy) along the MEP of the initiation reaction (using a model system). The insets show the local nondynamic correlation function[^102^, ^103^, ^104^] (iso=0.0025) at the points indicated in red.

Thus, the minimum‐energy path (MEP) on the singlet PES of the reaction of **1^H^** with MeBr was computed at the FIC‐NEVPT2/def2‐TZVP//UPBE‐D3/def2‐TZVP (Figure 7) as well as FIC‐NEVPT2/def2‐TZVP//CASSCF(4,4)/def2‐TZVP levels of theory. The results of both approaches are similar (Figure S18). The biradical character increases smoothly as the reaction progresses, ultimately leading to two separate radical species (Figures 7 and 8).

**Figure 9:**
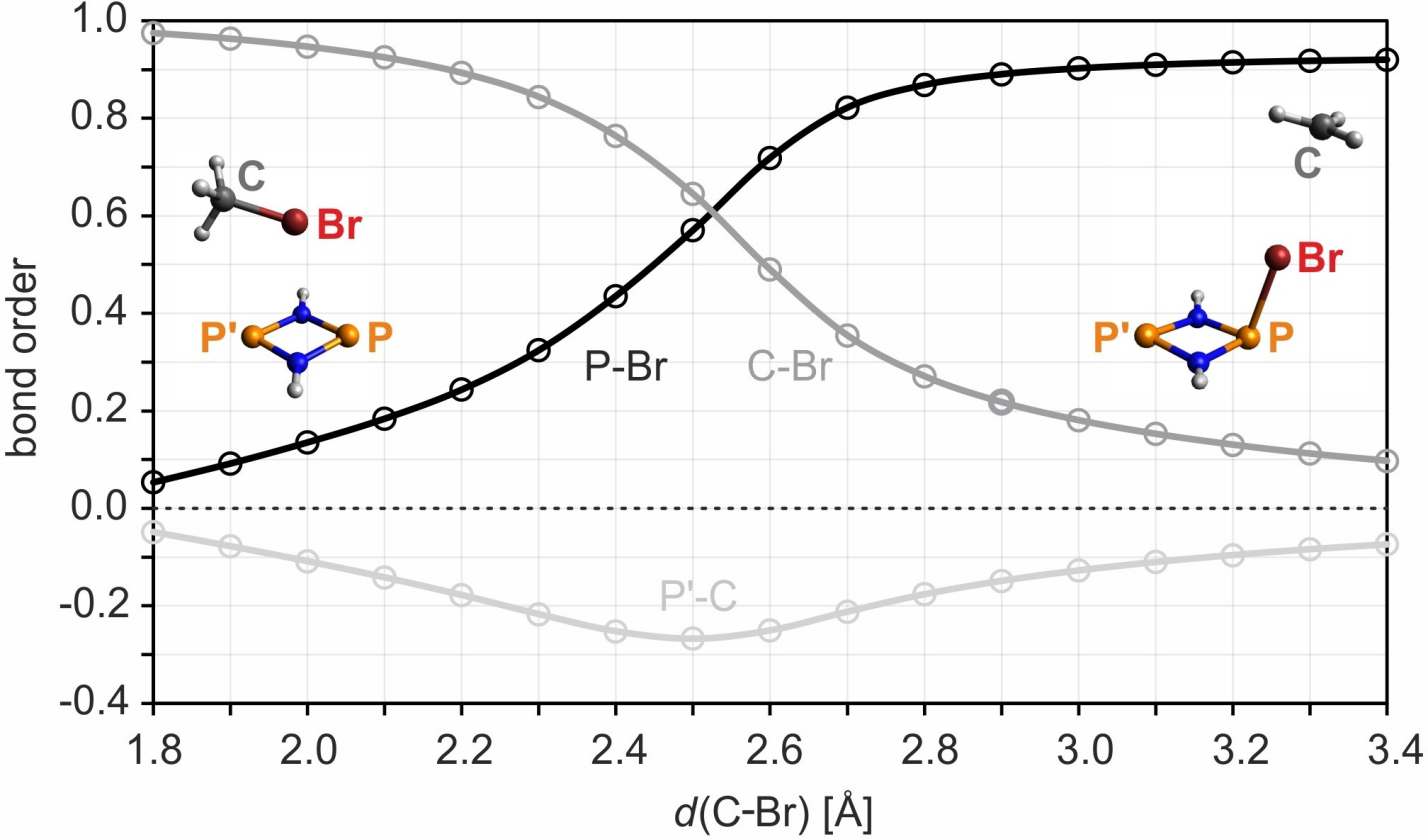
Change in the P−Br, C−Br, and P’−C bond orders along the MEP of the initiation reaction (using a model system). The C−Br bond order decreases (corresponding to the bond being broken), while the P−Br bond order increases smoothly. The interaction between the two radical centers (P’, C) in the product is slightly antibonding throughout the reaction.

Therefore, this initiation reaction (resulting in a radical chain reaction, see below and Scheme 6) is an intrinsically biradical process with a steadily decreasing singlet‐triplet gap and an increasing biradical character (Figures 7 and 8). At the end of the reaction, when only the two separate radicals **3^H^Br**⋅ and CH_3_⋅ are present, the singlet and triplet states are degenerate, that is, two separate doublet species are present. Along the path on the singlet PES, no barrier is found but the formation of the two doublet species is endergonic. For the separated molecules **1^H^** and MeBr, the biradical character (about 25 %) is attributed exclusively to the radical electrons at the two P atoms in **1^H^** (as represented by the blue isosurfaces of the local nondynamic correlation function[^102^, ^103^, ^104^] depicted in Figure 8). The biradical character increases dramatically over the course of the reaction, while one of the associated radical electrons shifts across the bromine atom to the methyl‐C atom. Note that the tetraradical character (Figure 8), although low throughout the process, reaches a maximum at about *d*(C−Br)=2.5 Å, where the C−Br and P−Br bond orders are roughly equal (Figure 9), indicating a small admixture of a Lewis‐type structure of the type ⋅P(μ‐NH)_2_P⋅⋯Br⋅⋯Me⋅ (see also Table S5 for a depiction of the active orbitals).

**Scheme 8:**
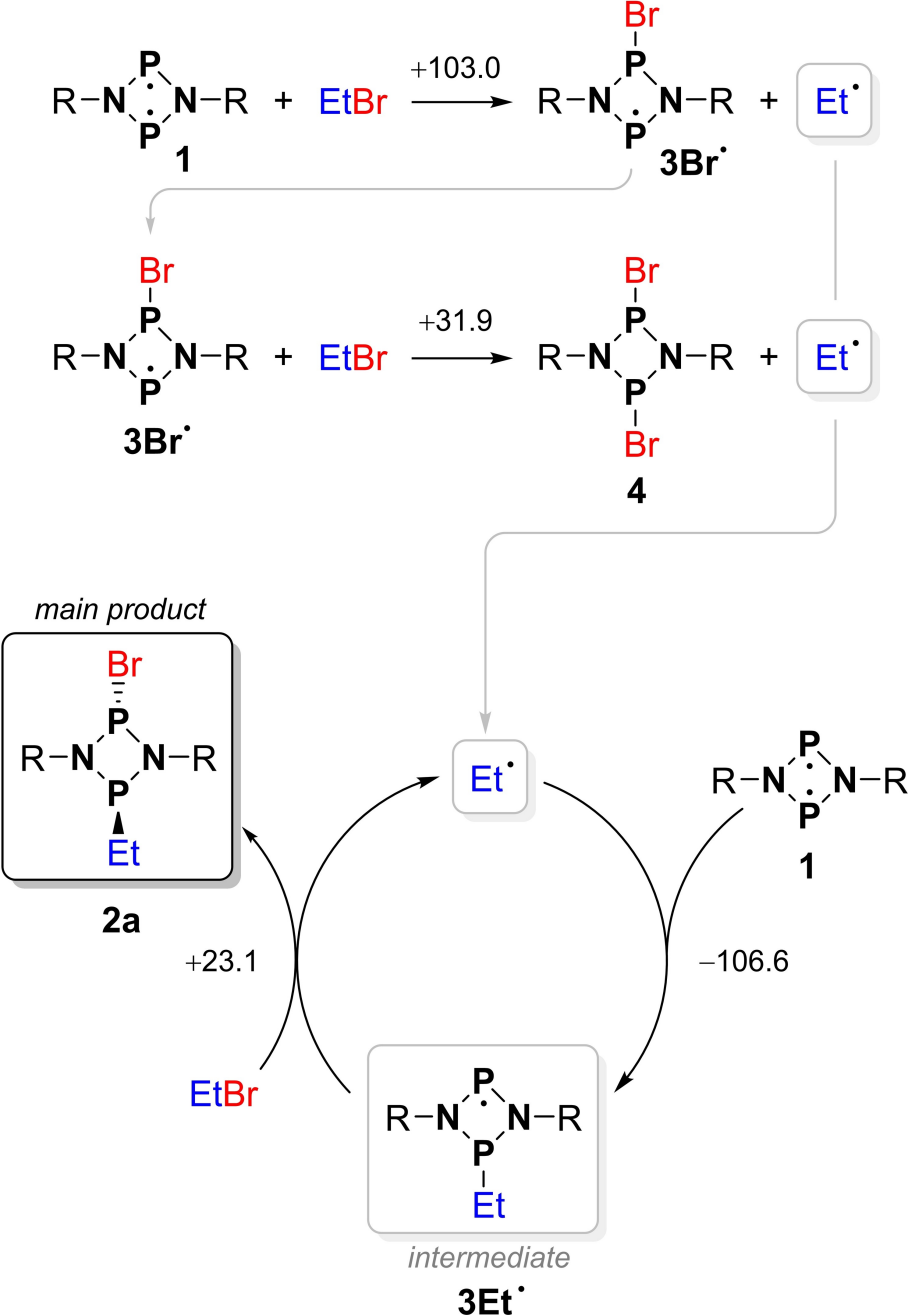
Proposed radical reaction mechanism. Free reaction energies (Δ_R_ *G*°, *p*°=1 atm) in kJ mol^−1^ (DLPNO‐CCSD(T)/def2‐TZVP//PBE‐D3/def2‐TZVP).

*Real system*. Possible radical reaction steps were computed using the actual molecular structures (i. e., including the Ter and Et substituents). The relevant steps are listed in Scheme 7 (a full set of all possible reactions can be found in Table S6). In agreement with our computations on the model system (see above), we identified the abstraction of Br⋅ from EtBr by the biradical [⋅P(μ‐NTer)_2_P⋅] (**1**) as the probable initiation reaction in an endergonic process (Δ_R_ *G*°=103 kJ mol^−1^) yielding **3Br**⋅. The formation of the ethyl‐substituted radical **3Et**⋅ in this first step is thermodynamically significantly less favored (Δ_R_ *G*°=149 kJ mol^−1^, cf. reactions 1 and 2 in Scheme 7). In this sense, the initiation reaction(s) may be understood as the “reaction barrier” for the radical process (cf. Scheme 8). Radical **3Br**⋅ now has different reactions channels it can follow: i) **3Br**⋅ can react with the free Et⋅ radical to give the (experimentally observed) final *trans* product **2** **a** in a highly exergonic reaction (Δ_R_ *G*°=−186.5 kJ mol^−1^, reaction 8 in Scheme 7). Therefore, the overall reaction **1**+EtBr → *trans‐* **2** **a** becomes energetically favored with −83.5 kJ mol^−1^ (reaction 9 in Scheme 7). However, this termination reaction is rather unlikely because the concentration of free Et⋅ radicals is very small compared to the concentration of EtBr. ii) **3Br**⋅ can further react with EtBr affording the experimentally observed by‐product **4** and Et⋅ (Δ_R_ *G*°=+31.9 kJ mol^−1^, reaction 5). The actual reaction turnover proceeds through addition of the free Et⋅ radical (which is initially generated by reactions 2 and 5 as discussed above) to the biradical [⋅P(μ‐NTer)_2_P⋅] (**1**), leading to the spectroscopically observed intermediate [⋅P(μ‐NTer)_2_P‐Et] (**3Et**⋅) in an exergonic reaction (Δ_R_ *G*°=−106.6 kJ mol^−1^, reaction 3). Radical **3Et**⋅ then reacts with another equivalent of EtBr to the product [Br‐P(μ‐NTer)_2_P‐Et] (**2** **a**) and a new Et⋅ radical (Δ_R_ *G*°=+23.1 kJ mol^−1^, reaction 4). The free Et⋅ radical can then repeat the reaction cascade outlined above (Scheme 8). All termination reactions are highly exergonic (Scheme 7 and Table S6).

Why is the radical **3Et**⋅ the crucial intermediate (which we do observe experimentally), but not the bromine‐substituted radical **3Br**⋅, which forms at the beginning of the reaction? This can be explained when considering the possible reactions of both radical species **3Et**⋅ and **3Br**⋅ with EtBr (e. g., reaction 4 in Scheme 7): The reaction of **3Br**⋅ with EtBr leads preferentially to the side product **4** (+31.9 kJ mol^−1^, reaction 5), while the reaction **3Br**⋅+EtBr→**2a**+Br⋅ is thermodynamically unfavored (+69.9 kJ mol^−1^, Table S6). In contrast, the formation of the product **2** **a** from **3Et**⋅ and EtBr (+23.1 kJ mol^−1^, reaction 4) is much more likely than the formation of the hypothetical by‐product [Et‐P(μ‐NTer)_2_P‐Et] (**5** **a**, +98.6 kJ mol^−1^), which was not observed experimentally (see above). Thus, formation of the final reaction product **2** **a** via the intermediate **3Et**⋅ corresponds to the minimum energy path on the potential energy surface.

As outlined above, the by‐product [Et‐P(μ‐NTer)_2_P‐Et] (**5** **a**) could in fact be generated when isolated **3Et**⋅ was treated with EtBr. This is also in line with our computed radical reaction steps (Scheme 7 and Table S6). In this case, **5** **a** is likely formed by the termination reaction **3Et**⋅+Et⋅ (−157.7 kJ mol^−1^, reaction 6), owing to the very large concentration of **3Et**⋅, which is at least six orders of magnitude larger than the concentration of the intermediately formed **3Et**⋅ in the reaction of **1** with EtBr. Thus, in the latter case, the same termination reaction is very unlikely to happen.

Finally, compared to *cis*‐**2** **a**, the formation of *trans*‐**2** **a** is energetically more favored by −24.3 kJ mol^−1^ (cf. reactions 9 and 10 in Scheme 7). This fact is best understood by looking at reaction 4 in Scheme 7. The formation of *trans*‐**2** **a** is associated with a Gibbs energy of +23.1 kJ mol^−1^, while the analogous reaction to the *cis*‐**2** **a** product requires +47.7 kJ mol^−1^ (Table S6). Since these radical reaction steps are equilibrium reactions, it is understandable that the formation of *trans*‐**2** **a** is thermodynamically significantly favored, which explains the exclusively observed formation of *trans*‐**2** **a**. It is also worth noting that a solution of pure *trans*‐**2** **a** does not isomerize to *cis*‐**2** **a**.

## Conclusion

We have demonstrated the radical reactivity of the biradical [⋅P(μ‐NTer)_2_P⋅] (**1**) for addition reactions of bromoalkanes. This approach represents a new synthesis route to asymmetrically substituted 1,3‐substituted *cyclo*‐1,3‐diphospha‐2,4‐diazanes. Extensive experimental and theoretical studies revealed [⋅P(μ‐NTer)_2_PEt] (**3Et**⋅) as a radical intermediate for the addition process of ethyl bromide to **1**. With the direct synthesis of **3Et**⋅, it was possible to obtain a hitherto unknown persistent phosphorus‐centered radical that can be generated on a large scale and stored for a long time under inert gas. It was possible to fully characterize **3Et**⋅, inter alia by EPR spectroscopy, single‐crystal X‐ray diffraction and UV‐vis spectroscopy. Reactivity studies of **3Et**⋅ with EtBr demonstrated its application in small‐molecule activation processes, and we believe that it has the potential to open a new field of activation chemistry involving phosphorus, due to its relatively high thermal stability and good accessibility.

## Experimental Section

All experiments were carried out under oxygen‐ and moisture‐free conditions in an inert argon atmosphere using standard Schlenk or dry‐box techniques. Experimental details including synthetic protocols, spectroscopic data and computational details can be found in the Supporting Information.

Deposition Numbers 2068187 (for **2** **a**), 2068188 (for **2** **b**), 2068189 (for **2** **c**), 2107762 (for **2** **d**), 2068190 (for **3Et**⋅), 2069052 (for **4**) contain the supplementary crystallographic data for this paper. These data are provided free of charge by the joint Cambridge Crystallographic Data Centre and Fachinformationszentrum Karlsruhe Access Structures service.

## Conflict of interest

The authors declare no conflict of interest.

1

## Supporting information

As a service to our authors and readers, this journal provides supporting information supplied by the authors. Such materials are peer reviewed and may be re‐organized for online delivery, but are not copy‐edited or typeset. Technical support issues arising from supporting information (other than missing files) should be addressed to the authors.

Supporting Information

## Data Availability

The data that support the findings of this study are available from the corresponding author upon reasonable request.
