# Supplementary material for: Radical Reactivity of the Biradical [⋅P(μ‐NTer)2P⋅] and Isolation of a Persistent Phosphorus‐Cantered Monoradical [⋅P(μ‐NTer)2P‐Et]
Source: Chemistry. 2022 May 19;28(36):e202200624. doi: 10.1002/chem.202200624 (PMC9322606; doi:10.1002/chem.202200624)
Supplement: Supplementary file 1 — Supporting Information [file CHEM-28-0-s001.pdf]

Chemistry–A European Journal

Supporting Information

Radical Reactivity of the Biradical [$^{\bullet}$P(μ-NTer)$_2$P$^{\bullet}$] and Isolation of a Persistent Phosphorus-Cantered Monoradical [$^{\bullet}$P(μ-NTer)$_2$P-Et]

Jan Rosenboom, Lukas Chojetzki, Tim Suhrbier, Jabor Rabeah, Alexander Villinger, Ronald Wustrack, Jonas Bresien,* and Axel Schulz*

SUPPORTING INFORMATION

This file includes:

1 Experimental ... 2

2 Structure elucidation ... 5

3 Syntheses of compounds ... 9

4 Additional spectroscopic details ... 37

5 Computational details ... 47

6 References ... 84

1 Experimental

General information. If not stated otherwise, all manipulations were carried out under oxygen- and moisture-free conditions in an inert argon atmosphere using standard Schlenk or drybox techniques. All glassware was heated three times *in vacuo* using a heat gun and cooled under argon atmosphere. Solvents were transferred using syringes, which were purged three times with argon prior to use. Solvents and reactants were either obtained from commercial sources or synthesized as detailed in Table S1.

Table S1: Origin and purification of solvents and reactants.

Substance	Origin	Purification
Et_2O, THF	local trade	dried over Na/benzophenone<br>freshly distilled prior to use
Benzene, toluene	local trade	dried over Na/benzophenone<br>freshly distilled prior to use
EtBr	local trade	dried over molecular sieve<br>freshly distilled prior to use
Br_2	Sigma Aldrich	dried over molecular sieve<br>freshly distilled prior to use
$CHBr_3$	local trade	dried over molecular sieve<br>freshly distilled prior to use
CH_2Br_2	local trade	dried over molecular sieve<br>freshly distilled prior to use
benzyl bromide	local trade	dried over molecular sieve<br>freshly distilled prior to use
C_6D_6	euriso-top	dried over Na<br>freshly distilled prior to use
C_7D_8	euriso-top	dried over Na<br>freshly distilled prior to use
$[^{\bullet}P(\mu\text{-NTer})_2P^{\bullet}]$	synthesized[1]	used as synthesized
Mg	abcr, 99.8%, for Grignards	activated by stirring under Argon for 3 weeks<br>using a glass-covered stir bar

NMR spectra were recorded on Bruker spectrometers AVANCE 250, AVANCE 300 or AVANCE 500 and were referenced internally to the deuterated solvent (C_6D_6 $\delta_{ref.}$ = 128.06 ppm, $\delta_{ref.,2}$ = 67.21 ppm, toluene-d_8 $\delta_{ref.,1}$ = 20.40 ppm, $\delta_{ref.,2}$ = 125.49 ppm), to protic impurities in the deuterated solvent (^{1}H: C_6HD_5 $\delta_{ref.}$ = 7.16 ppm, $\delta_{ref,2}$ = 3.58 ppm, toluene-d_7 $\delta_{ref,1}$ = 2.09 ppm, $\delta_{ref,2}$ = 6.98 ppm)[2] or externally (^{31}P: 85% H_3PO_4 $\delta_{ref.}$ = 0 ppm). All measurements were carried out at ambient temperature unless denoted otherwise. NMR signals were assigned using experimental data (e.g. chemical shifts, coupling constants, integrals where applicable).

IR spectra of crystalline samples were recorded on a Bruker Alpha II FT-IR spectrometer equipped with an ATR unit at ambient temperature under argon atmosphere. Relative intensities are reported according to the following intervals: very weak (vw, 0–10%), weak (w, 10–30%), medium (m, 30–60%), strong (s, 60–90%), very strong (vs, 90–100%).

Raman spectra of crystalline samples were recorded using a LabRAM HR 800 Horiba Jobin YVON Raman spectrometer equipped with an Olympus BX41 microscope with variable lenses. The samples were excited by a red laser (633 nm, 17 mW, air-cooled HeNe laser). All measurements were carried out at ambient temperature unless stated otherwise.

Elemental analyses were obtained using an Elementar vario Micro cube CHNS analyser.

Melting points (uncorrected) were determined using a Stanford Research Systems EZ Melt at a heating rate of 20 °C/min. Clearing points are reported.

DSC analyses were carried out at a heating rate of 5 °C/min using a Mettler-Toledo DSC 823e.

Mass spectra were recorded on a Thermo Electron MAT 95-XP sector field mass spectrometer using crystalline samples or an Advion Expression L benchtop mass spectrometer (*m/z* 10–2000) equipped with an Advion Expression CMS detector using sample solutions.

UV-vis spectra were acquired on a Perkin-Elmer Lambda 19 UV-Vis spectrometer or an Agilent Cary 60 UV-vis spectrometer using 6Q quartz cuvettes.

EPR spectra were recorded on a Bruker EMXCW micro spectrometer equipped with an ER 4119HS-WI high-sensitivity optical resonator.

2 Structure elucidation

X-ray Structure Determination: X-ray quality crystals were selected in Fomblin YR-1800 perfluoroether (Alfa Aesar) at ambient temperature. The samples were cooled to 123(2) K during measurement. The data were collected on a Bruker D8 Quest diffractometer or a Bruker Kappa Apex II diffractometer using Mo K_{α} radiation (λ = 0.71073 Å). The structures were solved by iterative methods (SHELXT)[3] and refined by full matrix least squares procedures (SHELXL).[4] Semi-empirical absorption corrections were applied (SADABS).[5] All non-hydrogen atoms were refined anisotropically, hydrogen atoms were included in the refinement at calculated positions using a riding model.

Table S2: Crystallographic details of **2a**, **2b**, **2c**·C_6H_6.

Compound	**2a**	**2b**	**2c**·C_6H_6
Chem. Formula	$C_{50}H_{55}BrN_2P_2$	$C_{49}H_{52}Br_2N_2P_2$	$0.9(C_{49}H_{51}Br_3N_2P_2)$ $\cdot 0.1(C_{48}H_{50}Br_2N_2P_2)\cdot C_6H_6$
Formula weight [g/mol]	825.81	890.68	1036.79
Colour	colourless	colourless	colourless
Crystal system	monoclinic	triclinic	triclinic
Space group	$P2_1/c$	P_i	P_i
a [Å]	20.485(4)	11.197(1)	11.916(1)
b [Å]	10.687(2)	21.339(1)	15.832(2)
c [Å]	19.906(3)	21.379(1)	25.720(2)
α [°]	90	117.502(2)	89.076(3)
β [°]	91.298(7)	100.306(2)	87.654(3)
γ [°]	90	98.619(2)	89.211(3)
V [$Å^3$]	4356.7(2)	4296.4(4)	4847.2(6)
Z	4	4	4
$\rho_{calcd.}$ [g/cm^3]	1.259	1.377	1.421
μ [mm^{-1}]	1.05	2.00	2.50
T [K]	123(2)	123(2)	123(2)
Measured reflections	69172	181593	218440
Independent reflections	9516	20736	25770
Reflections with $I > 2\sigma(I)$	6416	17603	18478
R_{int}	0.114	0.064	0.0722
F(000)	1736	1840	2124.3
$R_1(R[F^2>2\sigma(F^2)])$	0.054	0.098	0.0578
$wR_2(F^2)$	0.1492	0.2286	0.1299
GooF	1.014	1.289	1.093
No. of Parameters	509	1088	1144
diffractometer	Bruker Kappa Apex II	Bruker D8 Quest	Bruker D8 Quest
CCDC #	2068187	2068188	2068189

Table S3: Crystallographic details of **2d**, **3Et˙**, **4**.

Compound	**2d**	**3Et˙**	**4**
Chem. Formula	$C_{55}H_{57}BrN_2P_2$	$C_{50}H_{55}N_2P_2$	$C_{48}H_{50}Br_2N_2P_2$
Formula weight [g/mol]	887.87	745.90	876.66
Colour	yellow	brown	123(2)
Crystal system	monoclinic	monoclinic	triclinic
Space group	$P2_1$	$P2_1/n$	P_i
a [Å]	12.226 (2)	12.6560 (6),	8.5753(6)
b [Å]	12.377 (2)	21.0297 (9),	10.7613(8)
c [Å]	14.826 (2)	16.0437 (8)	11.3624(9)
α [°]	90	90	79.733(2)
β [°]	91.18 (3)	106.798 (2)	85.274(2)
γ [°]	90	90	81.975(2)
V [Å^3]	2243 (4)	4087.9 (3)	1019.9(2)
Z	2	4	1
$\rho_{calcd.}$ [g/cm^3]	1.315	1.212	1.427
μ [mm^{-1}]	1.03	0.14	2.102
T [K]	123 (2)	123(2)	123(2)
Measured reflections	22117	106549	9212
Independent reflections	10786	10861	9212
Reflections with $I > 2\sigma(I)$	6745	8374	7608
R_{int}	0.086	0.0479	0.0475
$F(000)$	932	1596	452
$R_1(R[F^2>2\sigma(F^2)])$	0.063	0.0490	0.0442
$wR_2(F^2)$	0.157	0.1372	0.1008
GooF	0.92	1.021	1.035
No. of Parameters	554	519	251
diffractometer	Bruker Kappa Apex II	Bruker D8 Quest	Bruker D8 Quest
CCDC #	2107762	2068190	2069052

Table S4: Selected bond lengths [Å], angles and dihedral angles [°]

Compound	**2a**	**2b**	**2c**	**2d**	**3Et˙**	**4**
P−N	1.697(3) – 1.770(3)	1.722(5)– 1.724(5)	1.710(2)– 1.745(3)	1.703– 1.783(6)	1.730(4) – 1.761(4)	1.726(2) – 1.730(2)
P−Br	2.420(1)	2.435(2)	2.327(1)	2.379(3)	-	2.311(1)
P−C	1.844(3)	1.81(2)	1.876(4)	1.851(8)	1.846(2)	-
P−P	2.616(2)	2.604(3)	2.605(2)	2.597(3)	2.631(1)	2.628(1)
NPN	79.1(2), 82.8(2)	81.8(2)	81.4(2), 82.6(2)	79.8(2), 83.0(3)	81.1(1), 82.1(1)	81.0(1)
PNP	97.9(2), 97.7(2)	98.2(2)	97.9(2), 97.8(2)	97.5(3), 96.3(3)	97.9(1), 98.0(1)	99.0(1)
NPNP	161.8(2)	180.0(5)	174.3(2)	159.0(4)	174.7(3)	180.0(1)

3 Syntheses of compounds

3.1 [BrP(μ-NTer)$_2$PEt] (2a)

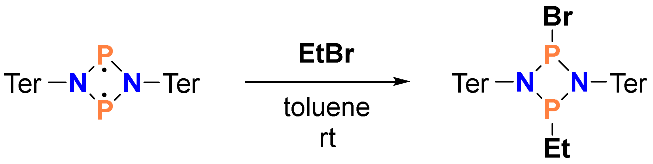

EtBr (76 mg, 0.69 mmol) is added to a solution of [$^{\bullet}$P(μ-NTer)$_2$P$^{\bullet}$] (500 mg, 0.69 mmol) in toluene (10 mL) at ambient temperature with a microliter syringe. The reaction mixture is stirred for two days, its colour slowly changing from orange to yellow over the course of the reaction. After two days, all volatile components are removed *in vacuo* (1×10^{-3} mbar) at 50 °C (water bath). The residue is dissolved in fresh toluene and insoluble solids are removed by filtration over celite. The clear solution is concentrated *in vacuo* (1×10^{-3} mbar) to incipient crystallization and stored overnight. Fine colourless crystals can be obtained. Even after several recrystallizations small impurities remain in the final product. The purity is estimated based on NMR integrals (^{31}P NMR spectrum) and is ca. 97%.

Yield: 512 mg (0.62 mmol, 89%, (97% purity based on ^{31}P NMR spectrum)).

Mp. 208-212 °C. **CHN** calcd. (found) in %: C 72.72 (71.83), H 6.71 (6.61), N 3.39 (3.21). **^{31}P{^{1}H} NMR** (C_6D_6, 202.5 MHz) δ = 229.0 (br s, 1 P, *P*-CH$_2$), 255.1 (br s, 1 P, *P*-Br). **^{1}H NMR** (C_6D_6, 500.1 MHz): δ = 0.91 (m, 3 H, P-CH$_2$-C*H*$_3$), 1.05 (m, 2 H, P-C*H*$_2$), 1.84 (s, 6 H, *o*/*p*-C*H*$_3$), 1.94 (s, 6 H, *o*/*p*-C*H*$_3$), 2.28 (s, 6 H, *o*/p-C*H*$_3$), 2.36 (s, 6 H, *o*/*p*-C*H*$_3$), 2.37 (s, 6 H, *o*/*p*-C*H*$_3$), 2.50 (s, 6 H, *o*/p-C*H*$_3$), 6.57-6.60 (m, 2 H, *m*-Ar*H*), 6.65 (br s, 2 H, *m*-Mes*H*), 6.69-6.72 (m, 2 H, *m*-Ar*H*), 6.71 (br s, 4 H, *m*-Mes*H*), 6.75-6.79 (m, 2 H, *p*-Ar*H*), 6.91 (br s, 2 H, *m*-Mes*H*). **^{13}C{^{1}H} NMR** (C_6D_6, 125.8 MHz) δ = 7.0 (br s, P-CH$_2$-CH$_3$), 21.1 (br s, *o*/*p*-CH$_3$), 21.9 (s, *o*/*p*-CH$_3$), 22.0 (s, *o*/*p*-CH$_3$), 22.1 (s, *o*/*p*-CH$_3$), 23.2-23.4 (m, *o*/*p*-CH$_3$), 29.3 (dd, P-CH$_2$, $^{1}J(^{13}C-^{31}P)$ = 49.6 Hz, $^{3}J(^{13}C-^{31}P)$ = 13.8 Hz), 123.3 (s, CH (arom.)), 128.3 (s, CH (arom.)), 128.7 (s, CH (arom.)), 128.8 (s, CH (arom.)) 130.2 (s, CH (arom.)), 132.1

(s, *C*H (arom.)), 132.2 (s, *C*H (arom.)), 135.8 (d, *C*, (arom.)), 136.5 (d, *C*, (arom.)), 136.8 (d, *C*, (arom.)), 137.4 (d, *C*, (arom.)), 138.1 (s, *C*, (arom.)), 138.7 (d, *C*, (arom.)), 139.3 (d, *C*, (arom.)), 141.8 (d, *C*, (arom.)). **IR** (ATR, 32 scans, cm^{-1}): $\tilde{\nu}$ = 2959 (w), 2914 (w), 2870 (w), 2852 (w), 2728 (vw), 1609 (w), 1576 (w), 1558 (vw), 1541 (vw), 1506 (vw), 1482 (w), 1455 (w), 1438 (w), 1403 (s), 1374 (w), 1265 (w), 1245 (w), 1216 (s), 1162 (w), 1082 (m), 1028 (w), 1006 (w), 983 (w), 964 (w), 946 (w), 900 (s), 880 (vs), 847 (s), 797 (s), 754 (s), 738 (w), 713 (m), 690 (w), 647 (w), 628 (w), 591 (w), 575 (w), 558 (w), 546 (m), 530 (m), 503 (w), 490 (w), 472 (w), 451 (m), 427 (w), 420 (w). **Raman** (633 nm, 15 s, 20 scans, cm^{-1}): $\tilde{\nu}$ = 3080 (3), 3073 (3), 3051 (3), 3039 (3), 3020 (3), 3004 (3), 2992 (3), 2988 (3), 2985 (3), 2978 (3), 2962 (3), 2954 (3), 2945 (3), 2920 (5), 2874 (3), 2860 (3), 2841 (3), 2834 (2), 2823 (2), 2818 (2), 2815 (2), 2798 (2), 2741 (2), 2733 (2), 1614 (4), 161 (3), 1582 (4), 1483 (2), 1419 (3), 1386 (2), 1306 (6), 1285 (3), 1277 (3), 1270 (3), 1244 (1), 1236 (1), 123 (6), 1191 (1), 1162 (1), 1105 (1), 1097 (1), 1031 (1), 1007 (2), 971 (1), 964 (1), 947 (1), 884 (2), 851 (1), 844 (1), 834 (1), 801 (1), 763 (1), 757 (1), 739 (3), 710 (1), 705 (1), 649 (1), 631 (2), 580 (7), 560 (3), 545 (3), 533 (3), 492 (1), 474 (1), 454 (4), 441 (1), 425 (1), 408 (1), 381 (1), 365 (1), 338 (2), 331 (2), 323 (2), 277 (2), 263 (2), 252 (4), 235 (4), 142 (6). **MS** (CI, pos., isobutane) *m/z*: 826 $[M]^+$, 745 $[(TerNP)_2Et]^+$, 716 $[(TerNP)_2]^+$, 388 $[TerNHPEt]^+$, 358 $[TerNP]^+$.

Single crystals suitable for X-ray diffraction can be grown from saturated benzene or toluene solution at ambient temperature (approx. 25°C).

Figure S1: NMR, IR and Raman spectra of **2a** (solvent signals are marked by an asterisk).

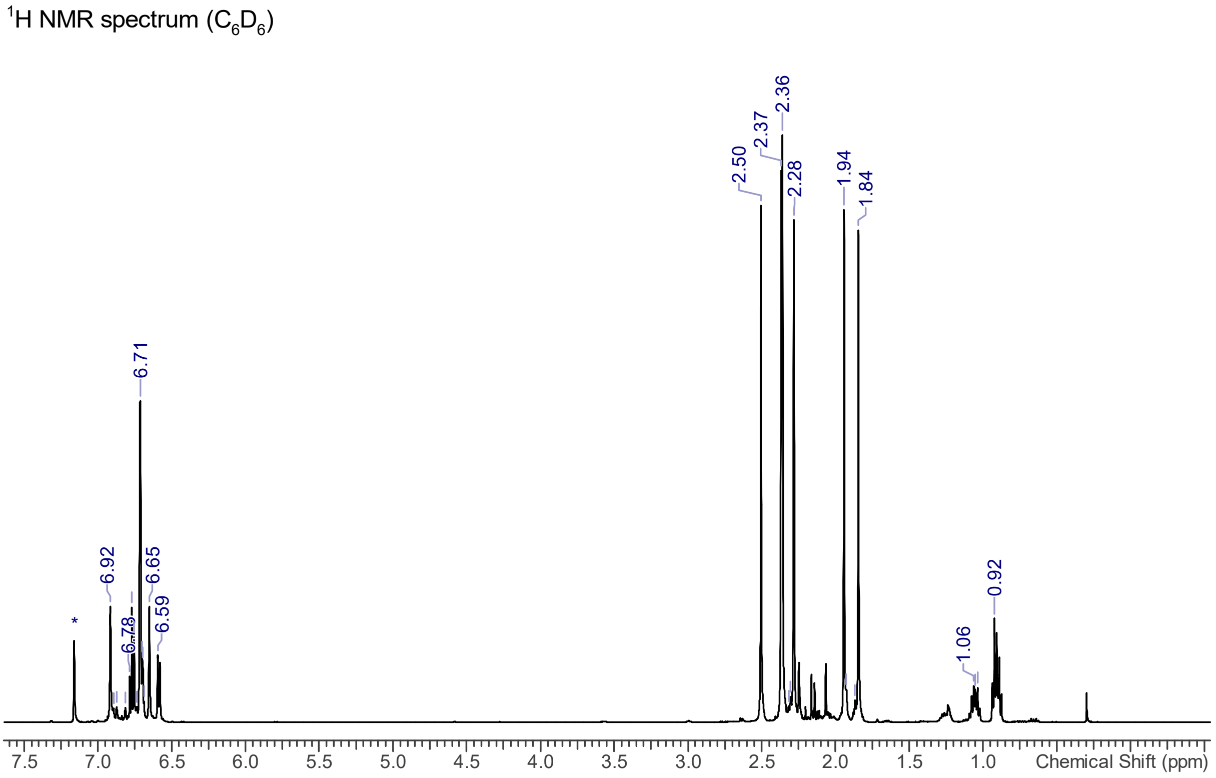

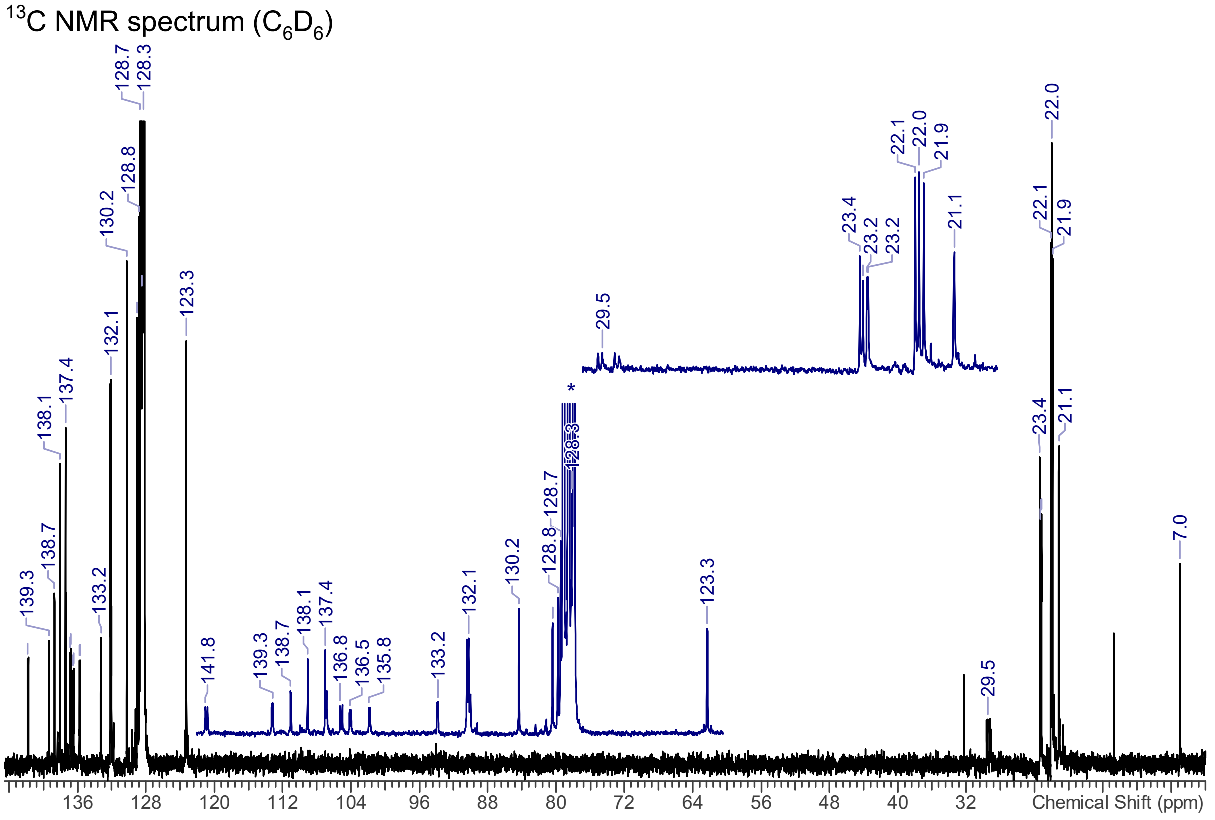

Figure S1 continued.

$^{31}P\{^{1}H\}$ NMR spectrum (C_6D_6)

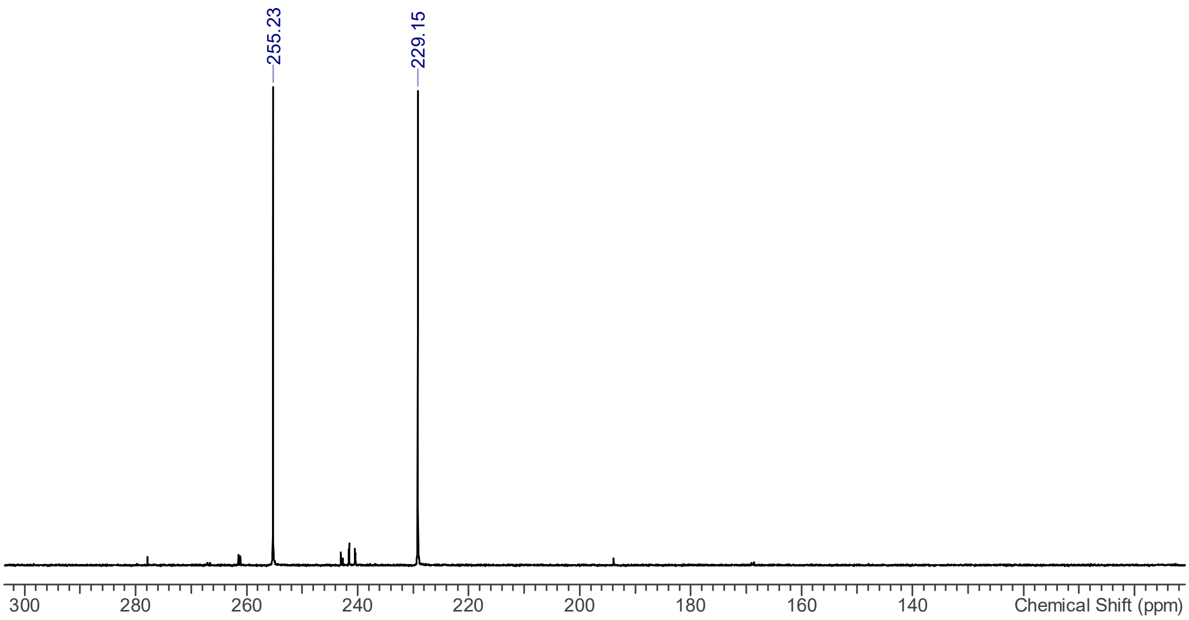

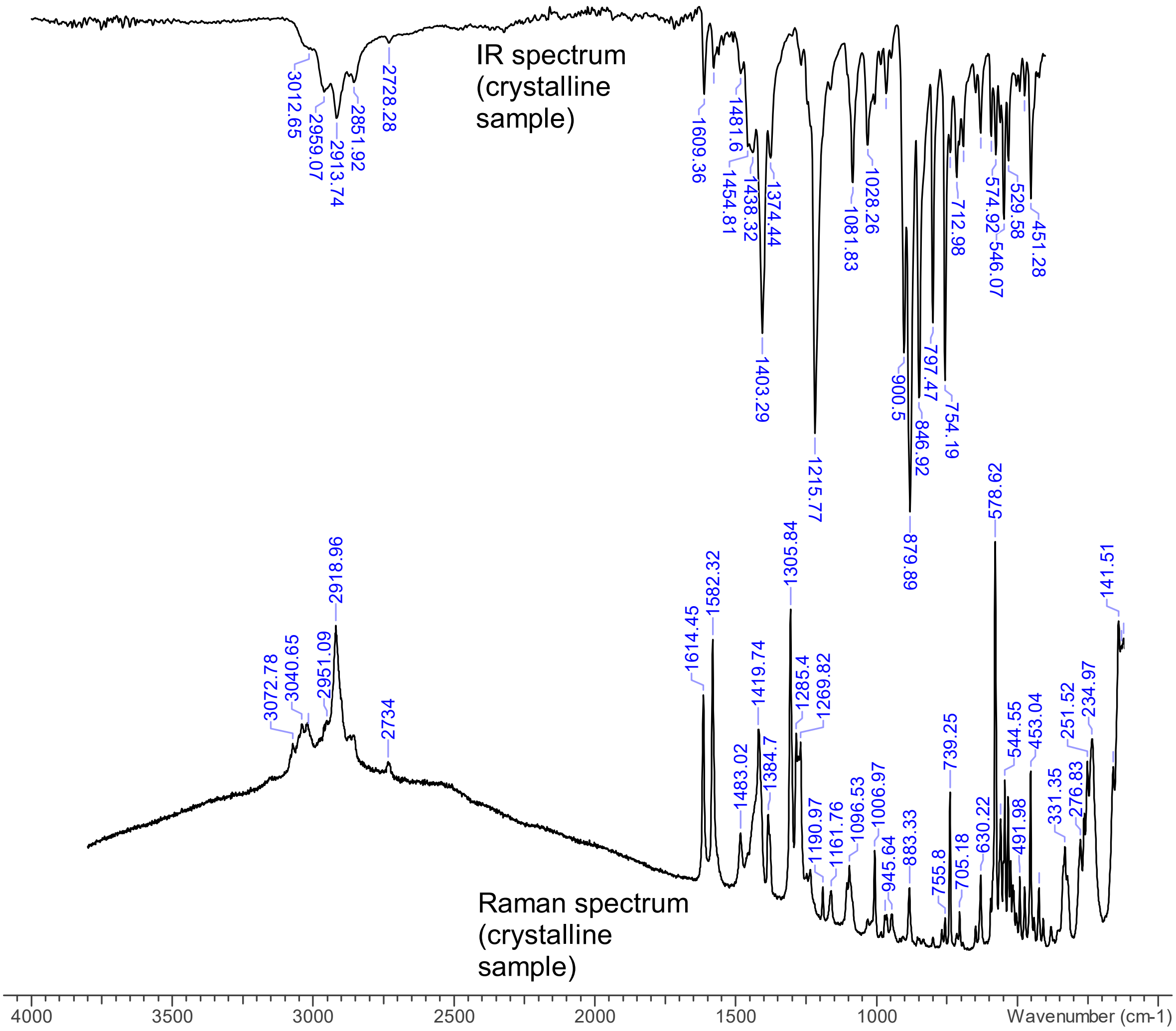

3.2 [BrP(μ-NTer)$_2$PCH$_2$Br] (2b)

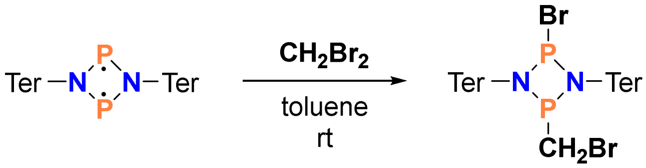

CH_2Br_2 (120 mg, 0.69 mmol) is added to a solution of [$^{\bullet}$P(μ-NTer)$_2$P$^{\bullet}$] (500 mg, 0.69 mmol) in toluene (10 mL) at rt with a microliter syringe. The reaction mixture is stirred for three hours, its colour slowly changing from orange to yellow over the course of the reaction. After the reaction time, all volatile components are removed *in vacuo* (1×10^{-3} mbar) at 50 °C (water bath). The residue is dissolved in fresh toluene and insoluble solids are removed by filtration over celite. The clear solution is concentrated *in vacuo* to incipient crystallization and stored overnight. After removal of the supernatant colourless crystals are obtained. Even after several recrystallizations small impurities remain in the final product. The purity is estimated based on NMR integrals (^{31}P NMR spectrum) and is ca. 91%. **Yield:** 403 mg (0.45 mmol, 65% (91% purity based on ^{31}P NMR spectrum).

Mp. 247-251 °C. **CHN** calcd. (found) in %: C 66.07 (66.66), H 5.88 (5.99), N 3.15 (3.16).**^{31}P{^{1}H} NMR** (C_6D_6, 202.5 MHz) δ = 195.0 (br s, 1 P, *P*-CH$_2$), 250.8 (s, 1 P, *P*-Br). **^{1}H NMR** (C_6D_6, 500.1 MHz): δ = 1.89 (s, 6 H, *o/p*-C*H*$_3$), 2.18 (s, 6 H, *o/p*-C*H*$_3$), 2.21 (s, 6 H, *o/p*-C*H*$_3$), 2.37 (s, 6 H, *o/p*-C*H*$_3$), 2.39 (s, 6 H, o/*p*-C*H*$_3$), 2.47 (s, 6 H, o/*p*-C*H*$_3$) 2.79 (d, 2 H, C*H*$_2$, ^{2}J (^{1}H-^{31}P) = 13 Hz), 6.50-6.54 (m, 2 H, *m*-Ar*H*), 6.59 (br s, 2 H, *m*-Mes*H*), 6.63 (br s, 2 H, *m*-Mes*H*), 6.70-6.74 (m, 2 H, *m*-Ar*H*), 6.73 (br s, 2 H, *m*-Mes*H*), 6.74-6.78 (m, 2 H, *p*-Ar*H*), 6.89 (br s, 2 H, *m*-Mes*H*). **^{13}C{^{1}H} NMR** (C_6D_6, 125.8 MHz) δ = 21.7 (s, *p/o*-CH$_3$), 22.0 (s, *p/o*-CH$_3$), 22.0 (s, *p/o*-CH$_3$), 22.1 (s, *p/o*-CH$_3$), 22.2-22.5 (m, *p/o*-CH$_3$), 23.0 (s, *p/o*-CH$_3$), 23.1 (s, *p/o*-CH$_3$), 23.3 (s (br.), *p/o*-CH$_3$), 34.5-35.2 (dd, CH$_2$, ^{1}J(^{13}C-^{31}P) = 68.0 Hz, ^{3}J(^{13}C-^{31}P) = 21.1 Hz), 123.4 (s, CH (arom.)), 128.6 (s, CH (arom.)), 129.0 (s, CH (arom.)), 129.0 (s, CH (arom.)), 130.0 (s, CH (arom.)), 131.8 (s, CH (arom.)), 132.5 (s, CH (arom.)), 135.7 (d, *C* (arom.)), 135.8 (d, *C* (arom.)), 137.7 (s, *C* (arom.)), 138.1 (s, *C* (arom.)), 138.5 (s, *C* (arom.)), 139.1 (d, *C* (arom.)), 141.9 (d, *C* (arom.)). **IR** (ATR, 32 scans,

cm^{-1}): $\tilde{\nu}$ = 3029 (vw), 3000 (w), 2971 (w), 2945 (w), 2914 (w), 2852 (w), 2728 (vw), 1609 (w), 1576 (w), 1558 (w), 1541 (vw), 1521 (vw), 1506 (vw), 1482 (w), 1455 (m), 1438 (m), 1403 (s), 1374 (m), 1339 (w), 1294 (vw), 1267 (w), 1245 (w), 1216 (s), 1162 (w), 1100 (w), 1082 (m), 1030 (w), 1016 (w), 1006 (w), 962 (w), 946 (vw), 900 (s), 882 (vs), 845 (s), 797 (s), 754 (s), 738 (m), 721 (m), 694 (w), 674 (w), 647 (w), 612 (w), 591 (w), 573 (m), 558 (w), 548 (m), 515 (m), 490 (w), 472 (w), 457 (w), 443 (m), 429 (m), 416 (w). **Raman** (633 nm, 15 s, 20 scans, cm^{-1}): $\tilde{\nu}$ = 3220 (1), 318 (2), 3149 (1), 3081 (1), 3071 (2), 3052 (1), 3040 (2), 3019 (1), 2945 (2), 2918 (4), 291 (9), 2856 (1), 2729 (1), 272 (3), 260 (6), 2543 (1), 2509 (1), 246 (3), 233 (5), 1612 (2), 1580 (10), 1561 (1), 1515 (1), 1507 (1), 1480 (2), 1417 (7), 1383 (2), 1361 (1), 1336 (1), 1302 (7), 1281 (7), 1265 (9), 1251 (2), 1233 (1), 1217 (1), 1188 (1), 1159 (1), 1101 (2), 1090 (3), 1063 (1), 1004 (1), 969 (1), 962 (1), 944 (1), 907 (1), 900 (1), 882 (1), 853 (1), 831 (1), 798 (1), 765 (1), 753 (1), 736 (3), 721 (1), 706 (1), 685 (2), 645 (1), 601 (5), 580 (3), 572 (5), 557 (4), 542 (2), 514 (9), 499 (1), 488 (2), 470 (2), 442 (2), 429 (1), 401 (1), 376 (1), 368 (1), 355 (1), 216 (6). **MS** (CI, pos., isobutane) *m/z*: 891 $[MH]^+$, 811, $[(TerNP)_2CH_2Br]^+$, 809 $[(TerNP)_2CH_2Br]^+$, 729 $[(TerNP)_2CH]^+$, 454 $[TerNPCH_2BrH]^+$, 453 $[TerNPCH_2Br]^+$, 452 $[TerNPCH_2BrH]^+$, 451 $[TerNPCH_2Br]^+$, 440 $[TerNPBrH]^+$, 438 $[TerNPBrH]^+$, 358 $[TerNP]^+$, 330 $[TerNH_3]^+$.

Single crystals suitable for X-ray diffraction can be grown from saturated benzene or toluene solution at rt.

Figure S2: NMR, IR and Raman spectra of **2b** (solvent signals are marked by an asterisk).

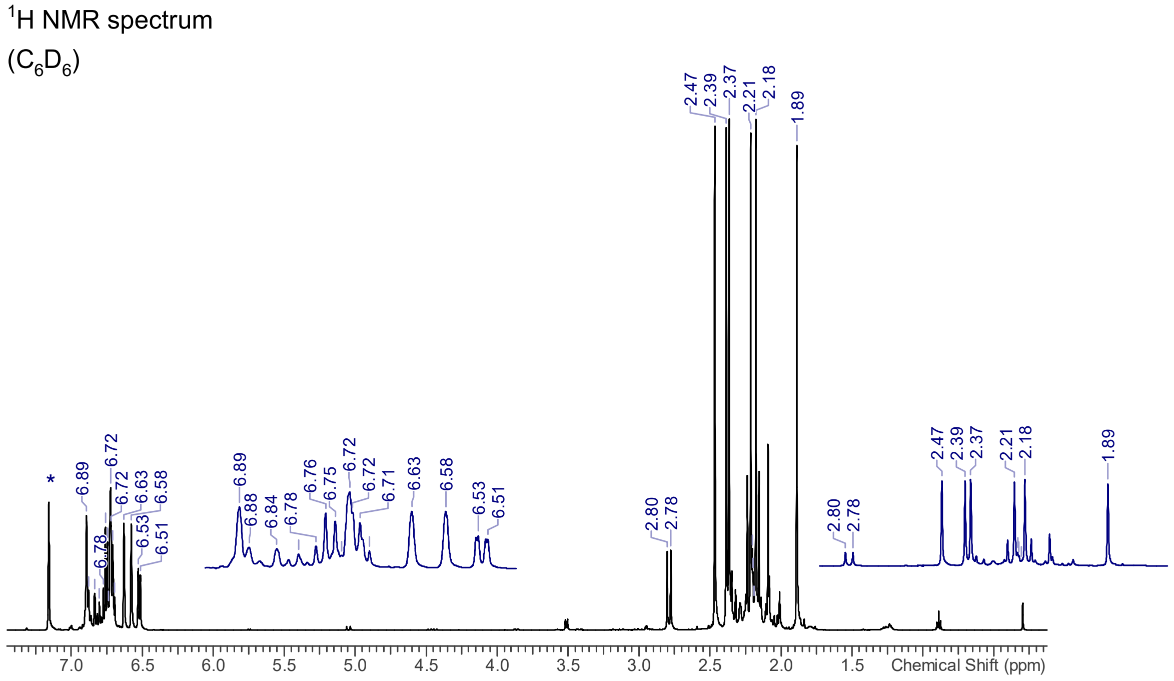

Figure S2 continued.

^{13}C NMR spectrum (C_6D_6)

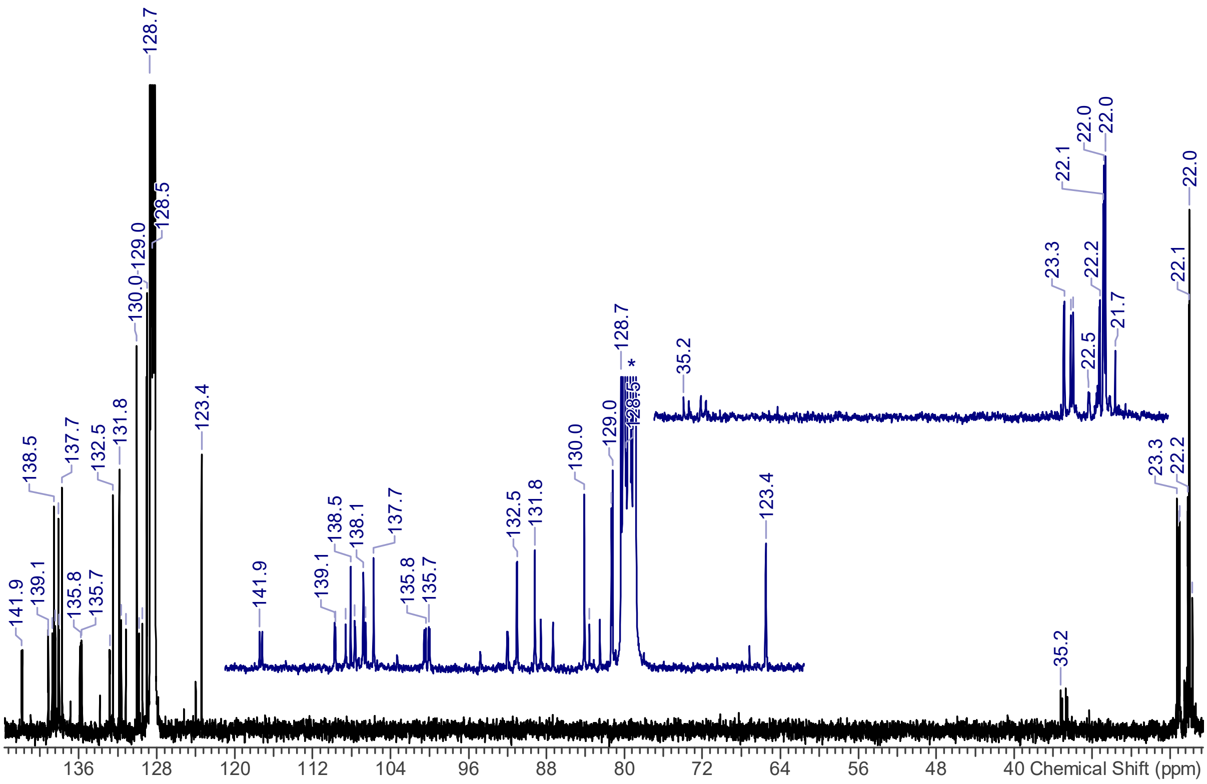

$^{31}P\{^1H\}$ NMR spectrum (C_6D_6)

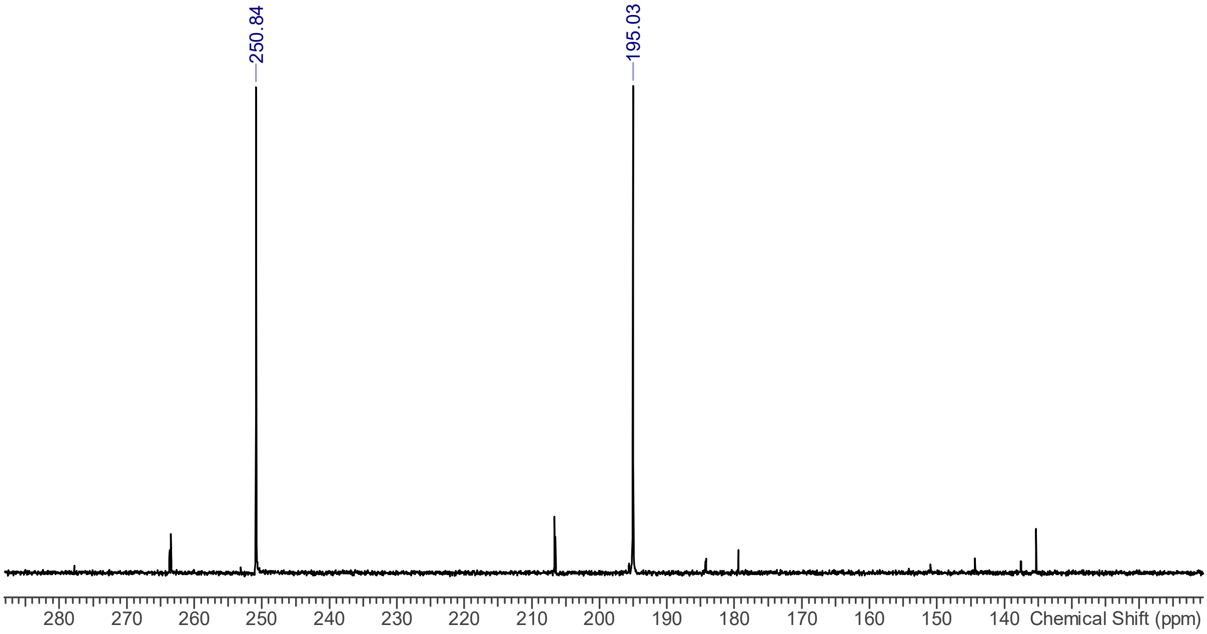

Figure S2 continued.

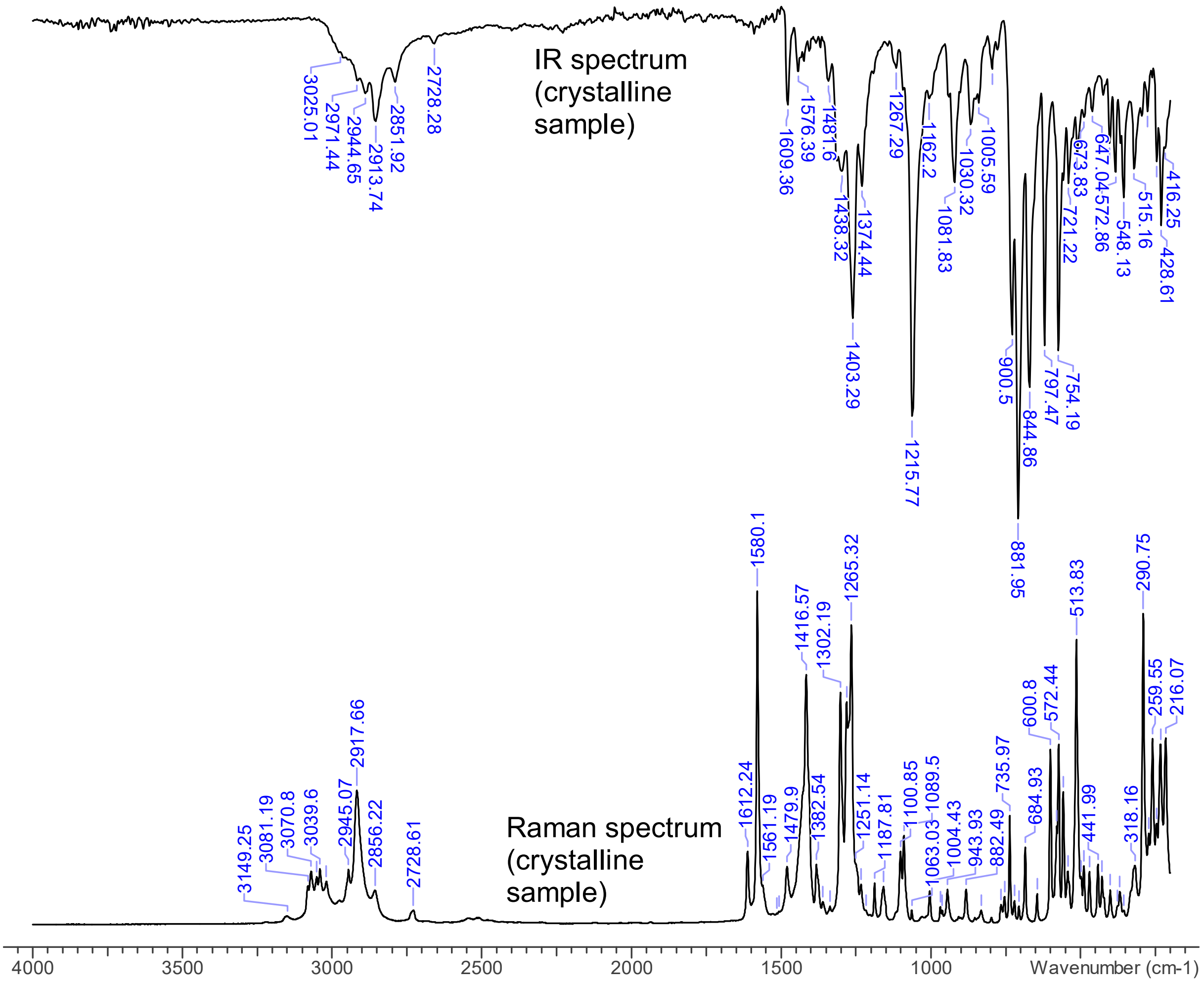

Figure S3: Molecular structure of **2b** in the crystal. Ellipsoids set at 50% probability (123 K). Selected bond lengths [Å] and dihedral angles [°]: N1–P1 = 1.722(5), N1–P1′ = 1.724(5), P1–Br1 = 2.435 (2), P1–C25 = 1.81(2), N1-P1-P2-N2 = 180.0(5).

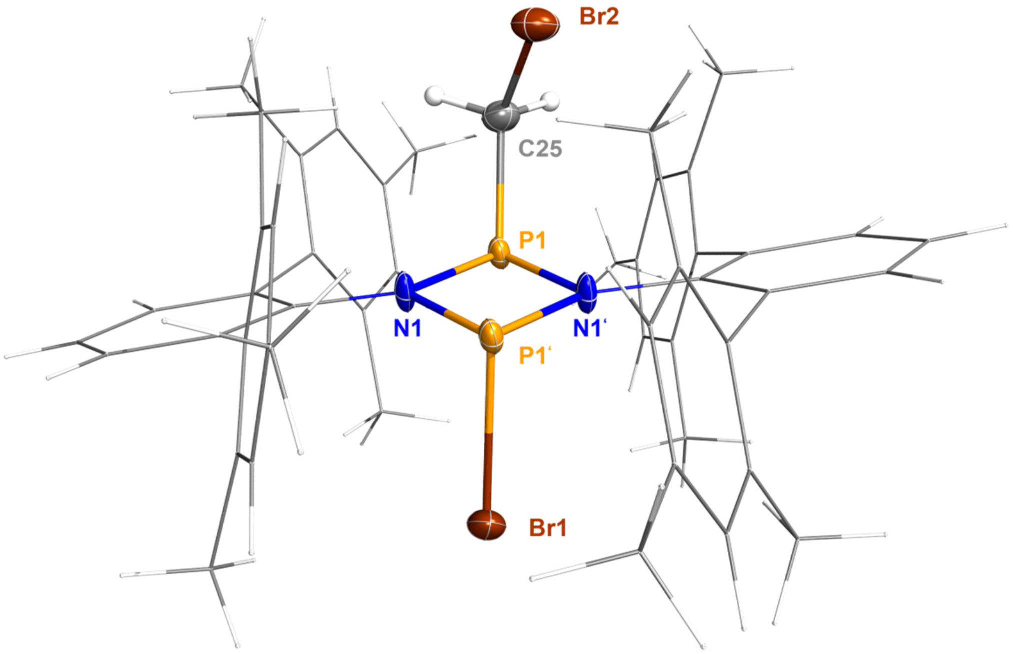

3.3 [BrP(μ-NTer)$_2$PCHBr$_2$] (2c)

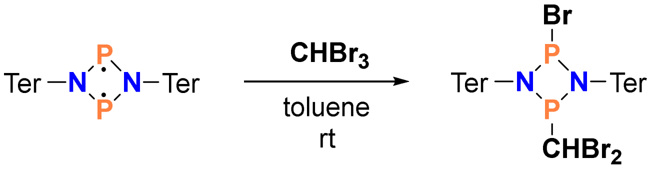

$CHBr_3$ (175 mg, 0.69 mmol) is added to a solution of [$^{\bullet}$P(μ-NTer)$_2$P$^{\bullet}$] (500 mg, 0.69 mmol) in toluene (10 mL) at rt with a microliter syringe. The reaction mixture is stirred for three hours, its colour slowly changing from orange to brown over the course of the reaction. After the reaction time, all volatile components are removed *in vacuo* (1×10^{-3} mbar) at 50 °C (water bath). The residue is dissolved in fresh toluene and insoluble solids are removed by filtration over celite. The clear solution is concentrated *in vacuo* (1×10^{-3} mbar) to incipient crystallization and stored overnight. After removal of the supernatant colourless crystals remain. Even after several recrystallizations small impurities remain in the final product. The purity is estimated based on NMR integrals (^{31}P NMR spectrum) and is ca. 93%. **Yield:** 466 mg (0.48 mmol, 69% (93% purity based on ^{31}P NMR spectrum)).

Mp. 185-190°C. **CHN** calcd. (found) in %: C 60.70 (60.87), H 5.30 (5.20), N 2.89 (2.77). **^{31}P{^{1}H} NMR** (C_6D_6, 101.3 MHz): δ = 210.7 (d, 1P, *P*-CHBr$_2$, ^{2}J(^{31}P-^{31}P) = 7.26 Hz), 278.0 (d, 1P, *P*-Br, ^{2}J(^{31}P-^{31}P) = 7.26 Hz). **^{1}H NMR** (C_6D_6, 250.1 MHz): δ = 1.96 (s, 6 H, *o*-C*H*$_3$), 2.12 (s, 6 H, *o*-C*H*$_3$), 2.26 (s, 6 H, *o*-C*H*$_3$), 2.28 (s, 6 H, *o*-C*H*$_3$), 2.34 (s, 6 H, *p*-C*H*$_3$), 2.47 (s, 6 H, *p*-C*H*$_3$), 5.58 (d, 1H, C*H*-Br$_2$, ^{2}J(^{1}H-^{31}P) = 15.3 Hz), 6.57-6.63 (m, 2 H, *m*-Ar*H*), 6.71-6.74 (m, 4 H, *m*-Mes*H/m*-Ar*H*), 6.75 (br s, 2 H, *m*-Mes*H*), 6.77-6.80 (m, 2 H, *p*-Ar*H*), 6.80-6.83 (m, 2 H, *m*-Mes*H*), 6.87-6.88 (m, 2 H, *m*-Mes*H*). **^{13}C{^{1}H} NMR** (C_6D_6, 62.9 MHz): δ = 21.9 (s, *o/p*-CH$_3$), 22.0 (s, *o/p*-CH$_3$), 22.0 (s, *o/p*-CH$_3$), 22.8 (s, *o/p*-CH$_3$), 23.0 (d, *o/p*-CH$_3$), 23.3 (s, *o/p*-CH$_3$), 23.3 (s, *o/p*-CH$_3$), 23.4 (s, *o/p*-CH$_3$), 49.8-51.2 (m, CHBr$_2$), 123.9 (s, CH (arom.)), 125.1 (s, *C* (arom.)), 128.9 (s, CH (arom.)), 129.1 (s, CH (arom.)), 129.4 (s, CH (arom.)), 129.6 (s, CH (arom.)), 129.8 (s, CH (arom.)) 132.0 (s, *C* (arom.)), 132.2 (s, *C* (arom.)), 132.3 (s, CH (arom.)), 132.6 (s, CH (arom.)), 133.7 (d, *C* (arom.)), 134.4 (s, *C* (arom.)), 135.3-135.8 (m, *C* (arom.)), 137.4-138.5 (m, *C* (arom.)), 139.4 (d, *C* (arom.)),

140.5 (s, *C* (arom.)), 140.7 (s, *C* (arom.)). **IR** (ATR, 32 scans, cm^{-1}): $\tilde{\nu}$ = 2992 (w), 2947 (w), 2912 (w), 2852 (w), 2726 (w), 2324 (vw), 1725 (vw), 1717 (vw), 1626 (vw), 1609 (w), 1591 (vw), 1480 (w), 1453 (m), 1438 (m), 1401 (s), 1374 (m), 1205 (s), 1160 (w), 1125 (w), 1086 (m), 1030 (m), 1006 (w), 946 (w), 900 (s), 878 (vs), 845 (vs), 795 (s), 767 (m), 750 (s), 740 (m), 694 (m), 645 (m), 596 (m), 575 (m), 558 (m), 550 (m), 540 (w), 509 (w), 495 (m), 482 (w), 447 (m), 441 (m), 427 (s). **Raman** (633 nm, 15 s, 20 scans, cm^{-1}): $\tilde{\nu}$ =3075 (1), 3072 (1), 3046 (1), 3018 (1), 3014 (1), 3006 (1), 2985 (1), 2972 (1), 2917 (2), 2872 (1), 2859 (1), 2731 (1), 268 (2), 232 (5), 204 (1), 1614 (4), 1582 (2), 1483 (1), 1412 (1), 1376 (2), 1306 (6), 1284 (2), 1272 (1), 1234 (1), 1193 (1), 1189 (1), 1159 (1), 1102 (1), 1007 (1), 947 (1), 889 (1), 882 (1), 852 (1), 800 (1), 741 (2), 710 (1), 698 (1), 660 (1), 652 (1), 593 (2), 582 (8), 566 (2), 552 (1), 541 (1), 526 (1), 511 (3), 488 (2), 478 (1), 436 (1), 423 (3), 399 (1), 376 (1), 359 (1), 337 (2), 319 (2), 306 (1), 178 (2), 175 (2), 158 (3). **MS** (CI, pos., isobutane) *m/z*: 887 $[(TerNP)_2CHBr_2]^+$, 807, 797 $[(TerNP)_2Br]^+$, 795, $[(TerNP)_2Br]^+$, 716 $[(TerNP)_2]^+$, 687, 450, 438 $[TerNPBrH]^+$, 386 $[TerNH_2C_4H_9]^+$, 358 $[TerNP]^+$, 330 $[TerNH_3]^+$.

Single crystals suitable for X-ray diffraction can be grown from saturated benzene or toluene solution at rt.

Figure S4: NMR, IR and Raman spectra of **2c** (solvent signals are marked by an asterisk).

^{1}H NMR spectrum (C_6D_6)

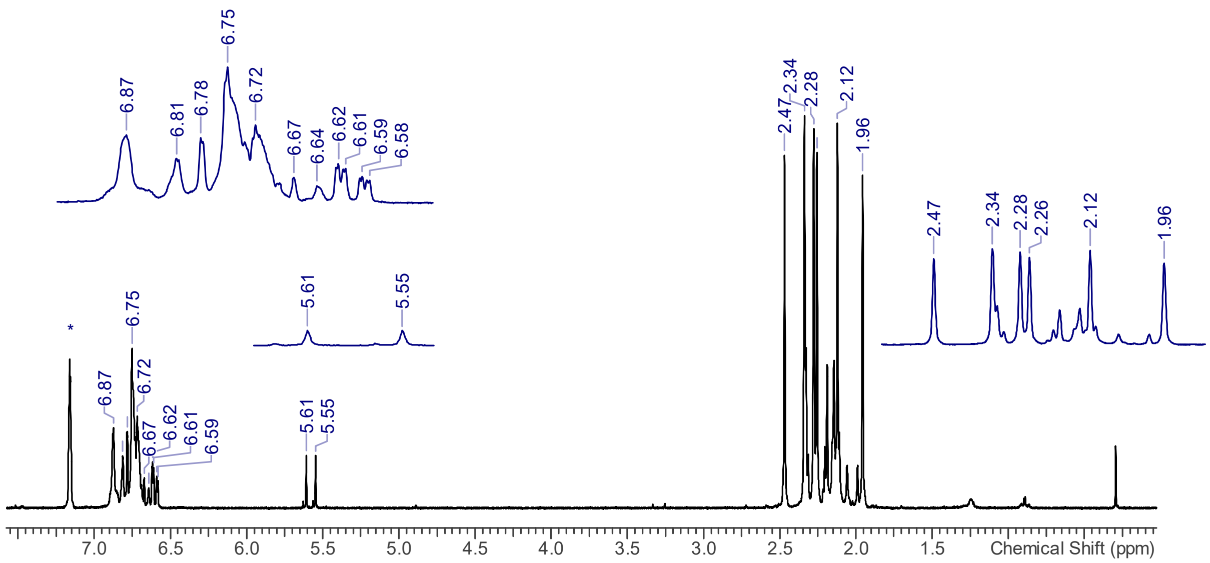

^{13}C NMR spectrum (C_6D_6)

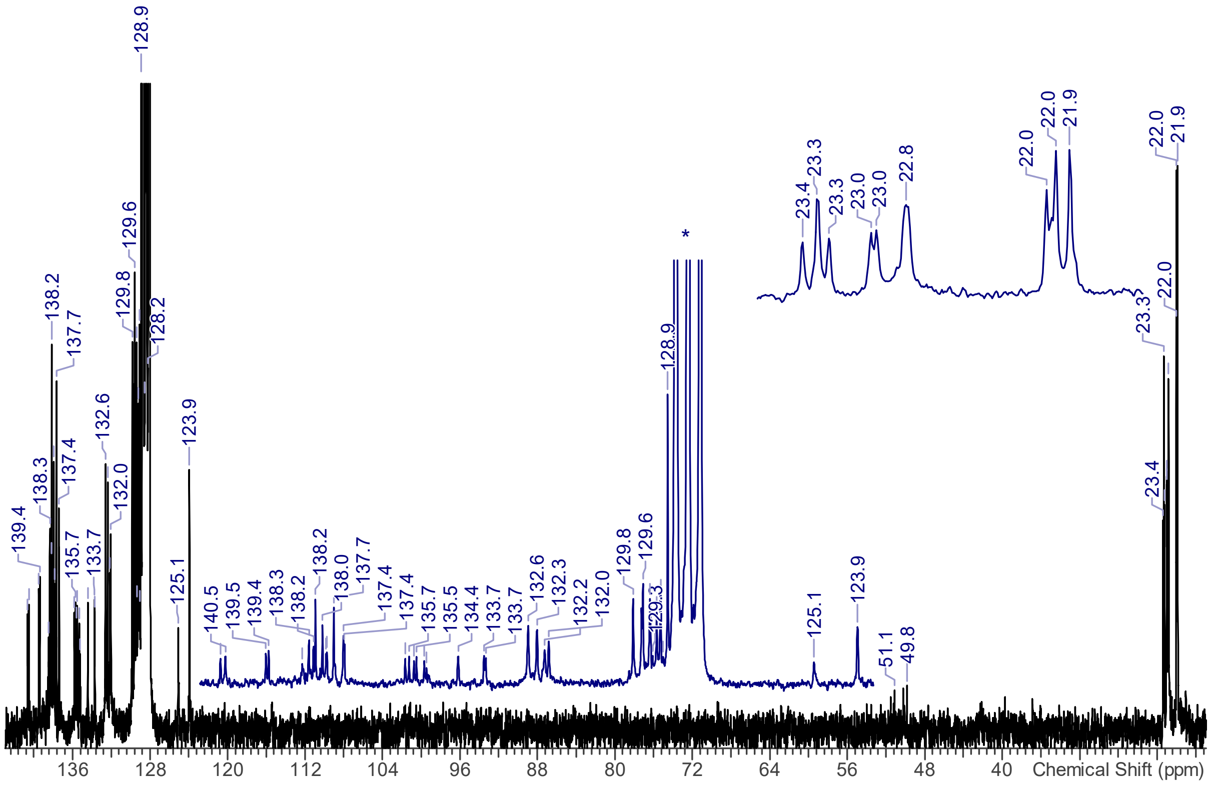

Figure S4 continued.

^{31}P NMR spectrum (C_6D_6)

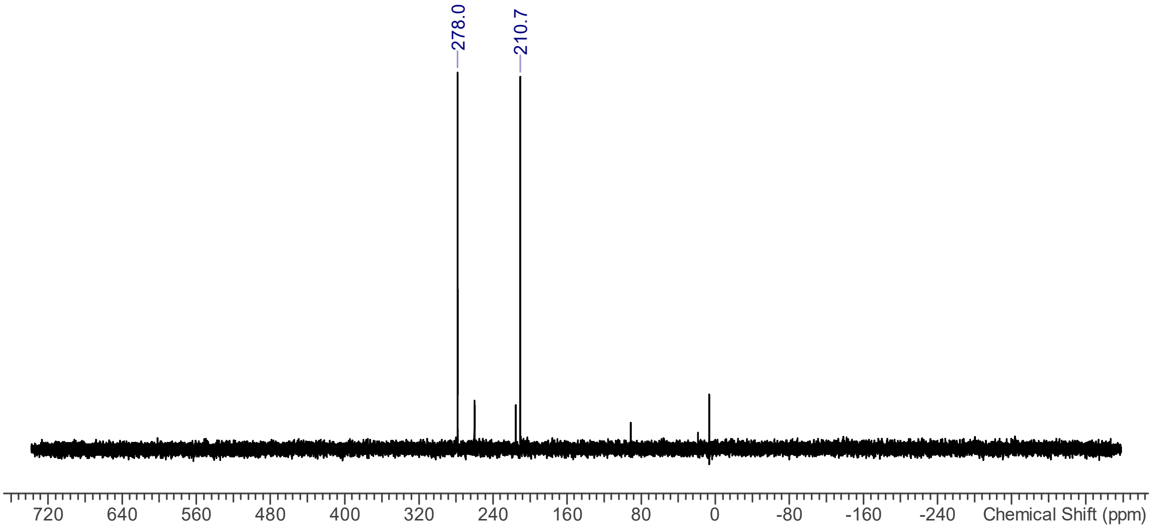

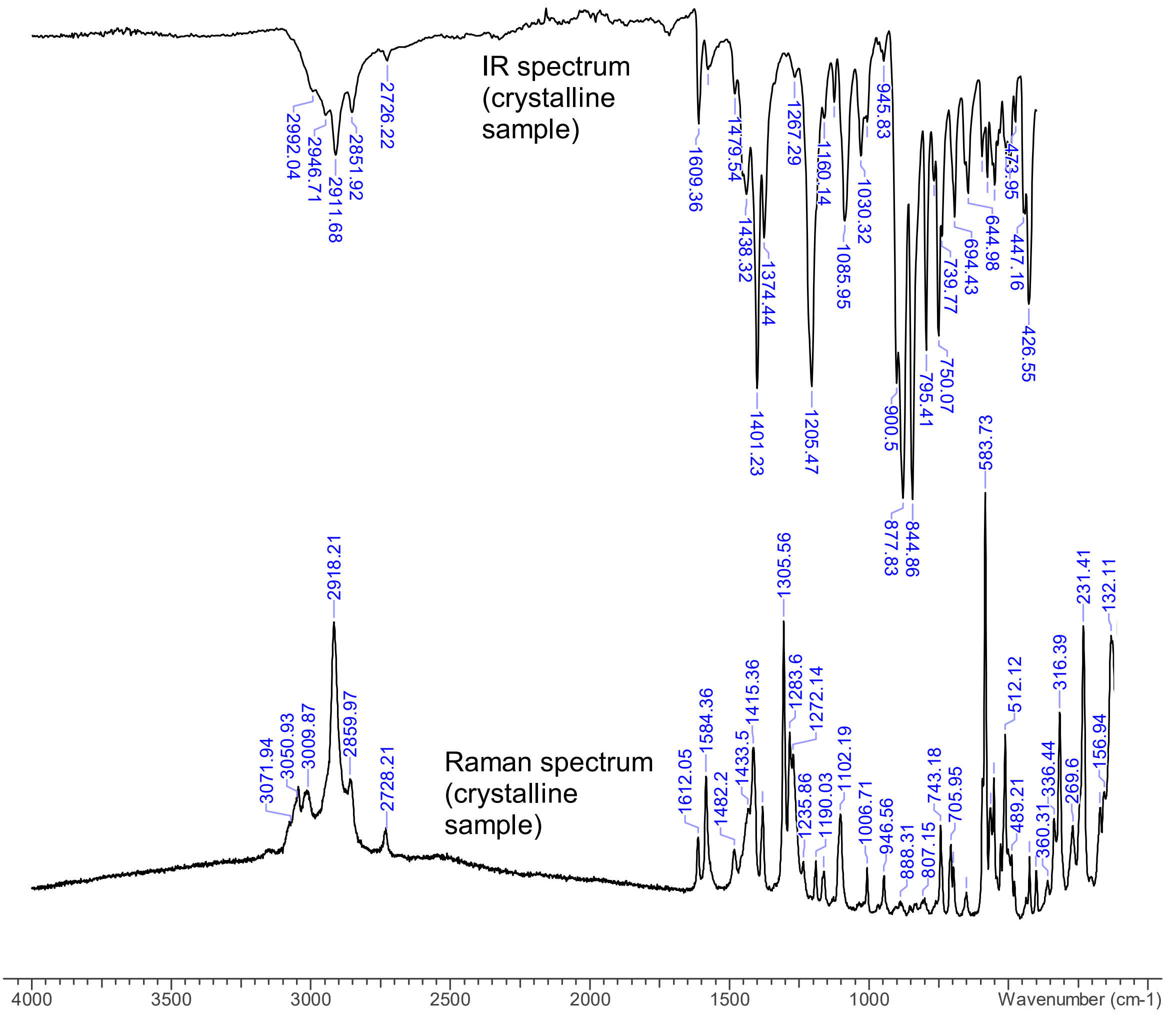

3.4 [BrP(μ-NTer)$_2$PBn] (2d)

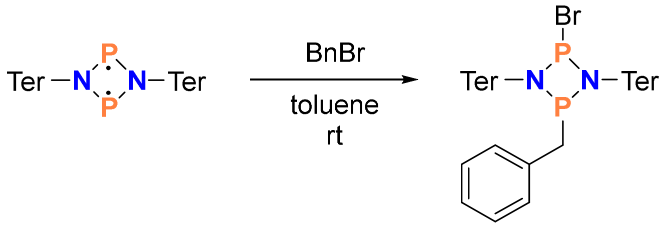

BnBr (120 mg, 0.69 mmol) is added to a solution of [$^{\bullet}$P(μ-NTer)$_2$P$^{\bullet}$] (500 mg, 0.69 mmol) in benzene (10 mL) at ambient temperature with a microliter syringe. Over one hour the reaction mixture is stirred, its colour slowly changing from orange to yellow after a few minutes. After the reaction time, all volatile components are removed *in vacuo* (1×10^{-3} mbar) at 50 °C (water bath). The residue is dissolved in fresh toluene and any insoluble solids are removed by filtration over celite. The clear solution is concentrated *in vacuo* (1×10^{-3} mbar) to incipient crystallization. Unfortunately the high amount of chemically very similar by-products made crystallisation very difficult. Only very few crystals suitable for X-ray single crystal diffractometry could be obtained after several weeks of storage at 5 °C.

It was possible to use this crystalline sample for the Raman/IR/MS measurements, however it was insufficient for further NMR measurements.

Therefore, NMR spectra were obtained from the reaction mixture and only the ^{31}P NMR signals could be assigned. **Yield:** 340 mg (~80 % purity, 0.38 mmol, 55%), 20 mg (0.022 mmol, pure)

Mp. 210-215 °C **^{31}P{^{1}H} NMR** (C_6D_6, 202.5 MHz) δ = 242.5 (s, 1 P, *P*-CH), 274.3 (s, 1 P, *P*-Br). **IR** (ATR, 32 scans, cm^{-1}): $\tilde{\nu}$ = 2943 (w), 2914 (w), 2853 (w), 1610 (w), 1480 (w), 1439 (m), 1402 (s), 1375 (m), 1206 (s), 1086 (m), 1031 (w), 901 (s), 878 (vs), 845 (vs), 796 (s), 752 (s), 740 (m), 676 (m), 647 (w), 427 (m). **^{1}H NMR** (C_6D_6, 300.1 MHz): δ = 1.48 (s), 1.81 (s), 2.10-2.15 (m), 2.25 (s), 2.28 (s), 2.34-2.38 (m), 2.59 (s), 3.13 (s), 6.55-6.84 (m), 6.90 (s, br), 7.05-7.07 (m) **Raman** (633 nm, 15 s, 20 scans, cm^{-1}): $\tilde{\nu}$ =3039 (1), 2918 (3), 2858 (1), 2732 (1), 1611 (4), 1579 (1), 1481 (1), 1437 (1), 1408 (1), 1380 (2), 1304 (6),

1282 (1), 1270 (1), 1256 (1), 1239 (1), 1186 (1), 1164 (1), 1157 (1), 1099 (1), 1094 (1), 1077 (1), 1029 (1), 1003 (3), 991 (1), 953 (1), 943 (1), 819 (1), 738 (2), 624 (1), 618 (1), 598 (1), 577 (6), 558 (2), 549 ((1), 520 (3), 498 (1), 488 (1), 474 (1), 459 (1), 439 (1), 421 (1), 406 (1), 395 (1), 373 (1), 355 (1), 331 (2), 321 (1), 270 (1), 245 (1), 227 (2). **MS** (CI, pos., isobutane) *m/z*: 887 $[M]^+$, 807, $[(TerNP)_2CH_2Ph]^+$, 797 $[(TerNP)_2Br]^+$, 795 $[(TerNP)_2Br]^+$, 716 $[(TerNP)_2]^+$, 450 $[TerNP\ CH_2PhH]^+$, 358 $[TerNP]^+$, 91 $[PhCH_2]^+$.

Figure S5: NMR, IR and Raman spectra of **2d** (solvent signals are marked by an asterisk).

^{1}H NMR spectrum (C_6D_6)

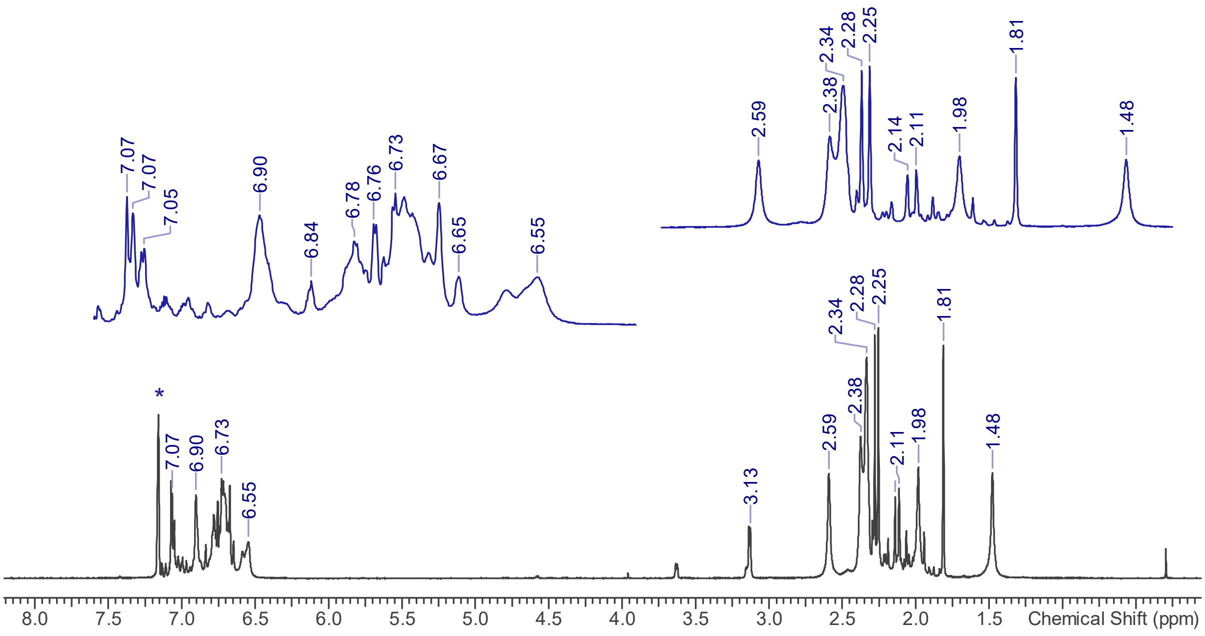

Figure S5 continued.

^{31}P NMR spectrum (C_6D_6)

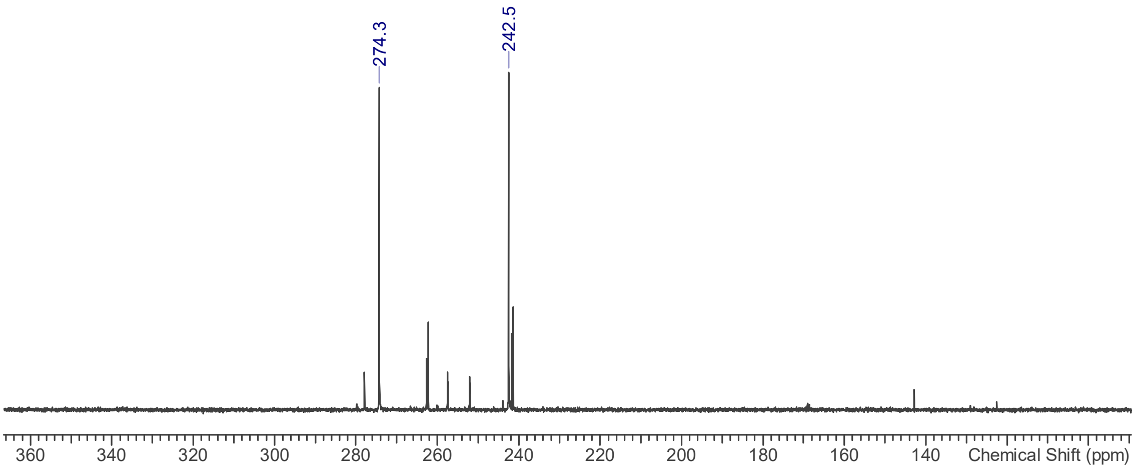

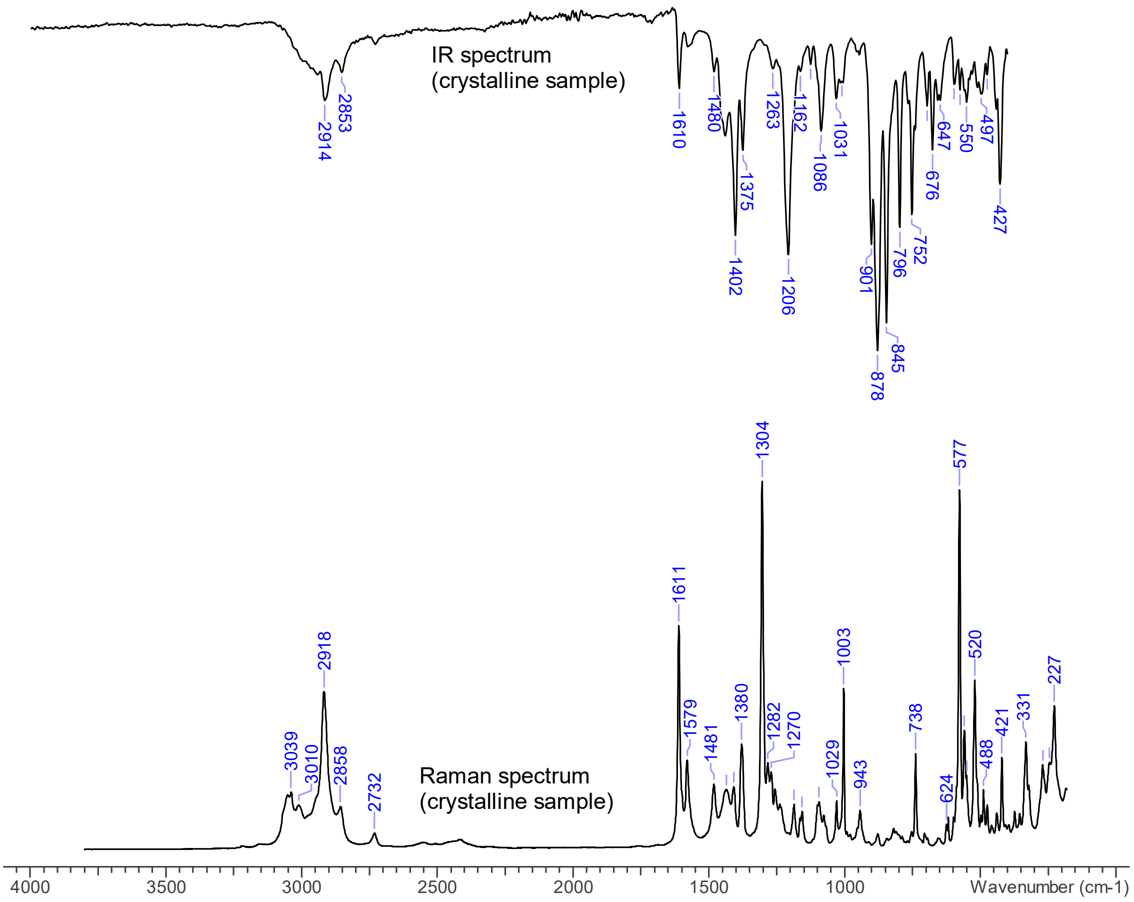

Figure S6: Molecular structure of **2d** in the crystal. Ellipsoids set at 50% probability (123 K). Selected bond lengths [Å] and dihedral angles [°]: N1–P1 = 1.783(6), N1–P2 = 1.703(6), N2–P2 = 1.710(6), N2–P1 = 1.745(6), P1–Br1 = 2.379 (3), N1-P1-P2-N2 = 159.0(4).

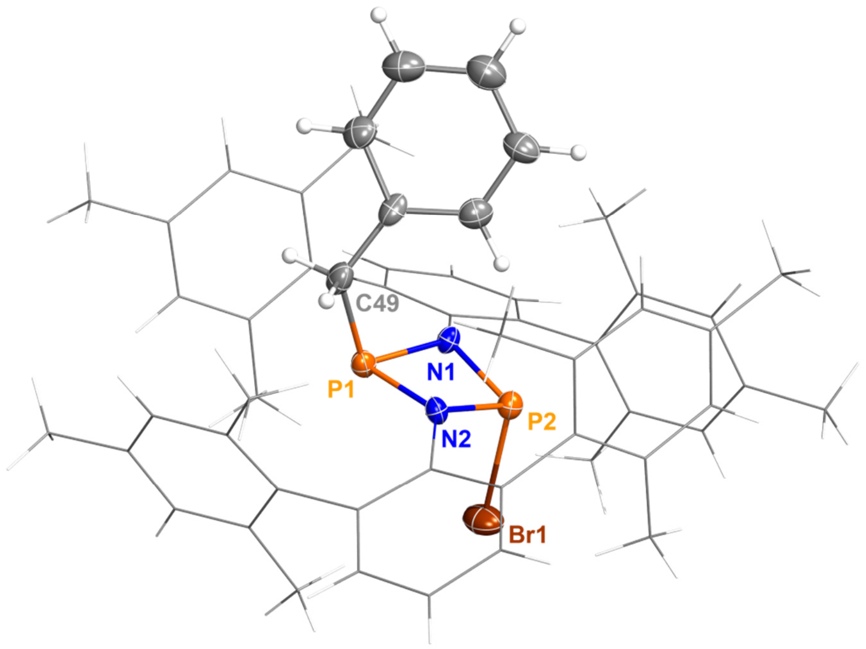

3.5 [$^{\bullet}$P(μ-NTer)$_2$PEt] (3Et$^{\bullet}$)

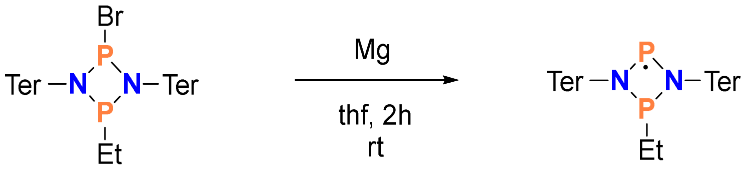

Mg (44 mg, 1.8 mmol) and [BrP(μ-NTer)$_2$PEt] (**2a**) (200 mg, 0.242 mmol) are combined and THF (8 mL) is added at ambient temperature and the mixture is stirred. As the reaction progresses, the colour of the solution changes from yellow to dark red. After the beginning of the colour change the solution is stirred for two hours and then the remaining magnesium is removed by filtration. The solvent is removed from the filtrate *in vacuo* (1×10^{-3} mbar) and the remaining solid is dissolved in toluene and then filtrated again to remove $MgBr_2$. The solvent is once again removed in vacuo, the solid dissolved in THF and *in vacuo* (1×10^{-3} mbar) to incipient crystallization and stored at ambient temperature overnight. After removal of the supernatant dark red crystals remain. **Yield:** 151 mg (0.203 mmol, 83%).

As species **3Et$^{\bullet}$** is paramagnetic, no signals were observed in the NMR spectra. Signals visible in the ^{31}P spectrum are impurities in very low concentrations (e.g. starting material) that were only visible due to increased number of scans (2048). Signals in ^{1}H NMR spectrum are also very low intensity (note the solvent residual signal for comparison) and can also be attributed to impurities.

Mp. 195°C. **Raman** (633 nm, 10 s, 10 scans, cm^{-1}): $\tilde{\nu}$ =3050 (1), 3046 (1), 3010 (1), 2999 (1), 2987 (1), 2981 (1), 2958 (2), 2918 (4), 2875 (2), 2860 (2), 2733 (1), 1613 (3), 1581 (3), 1484 (1), 1438 (2), 1419 (2), 1381 (2), 1304 (7), 1283 (2), 1235 (1), 1188 (1), 1165 (1), 1098 (1), 1093 (1), 1005 (1), 943 (1), 913 (1), 880 (1), 740 (1), 704 (1), 577 (5), 559 (2), 523 (1), 512 (1), 487 (1), 421 (1), 334 (1), 294 (1), 268 (1), 229 (1). **IR** (ATR, 32 scans, cm^{-1}): $\tilde{\nu}$ = 2943 (w), 2914 (w), 2855 (w), 1721 (vw), 1610 (w), 1577 (w), 1482 (vw), 1453 (w), 1437 (w), 1400 (s), 1373 (m), 1282 (vw), 1233 (vs), 1187 (w), 1158 (w), 1119 (w), 1078 (m), 1029 (m), 1004 (w), 983 (w), 944 (vw), 899 (s), 878 (m), 868 (s), 845 (s), 791 (m), 763 (w), 746 (s), 711 (w), 690 (w), 645 (vw), 622 (w), 585 (w), 573 (w), 548 (w), 540 (w), 526

(w), 497 (w), 439 (w). **MS** (ESI, THF, pos.) *m/z*: 745 $[M]^+$. **UV-vis** (toluene, 0.2 mmol/L): λ = 399 (0.93). **EPR** (800 G): g = 2.003 (d, A = 59 G).

Figure S7: NMR, IR, Raman and mass spectra of **3Et˙** (solvent signals are marked by an asterisk).

^{31}P NMR spectrum (C_6D_6)

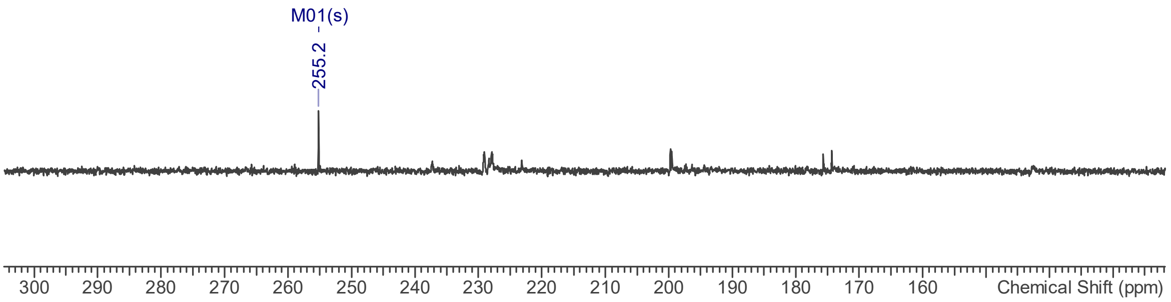

Figure S7 continued.

^{1}H NMR spectrum (C_6D_6)

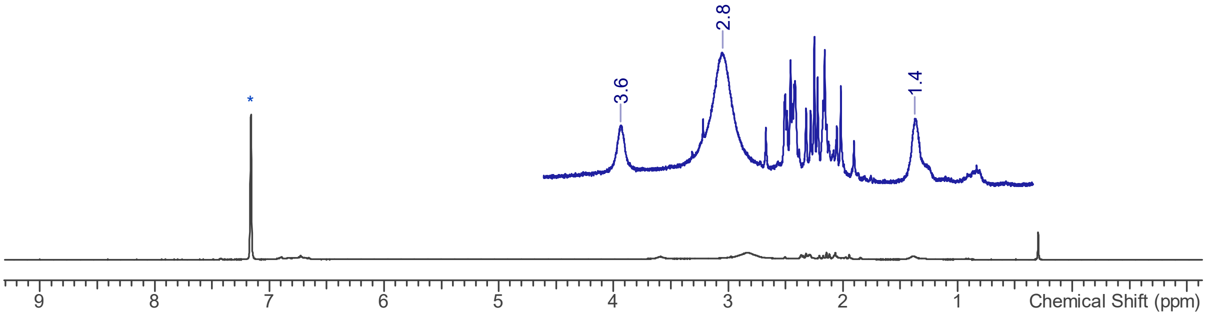

Figure S7 continued.

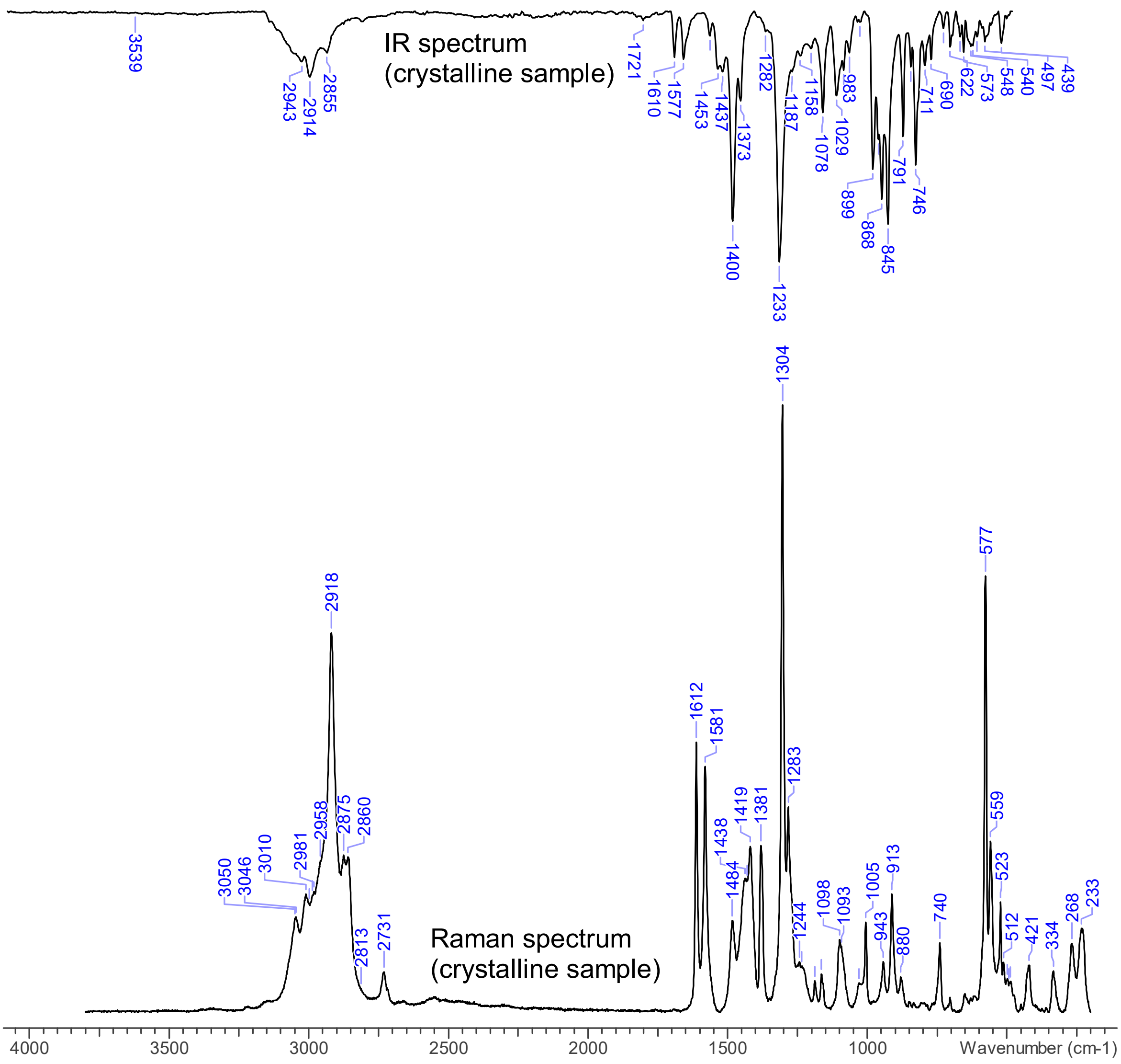

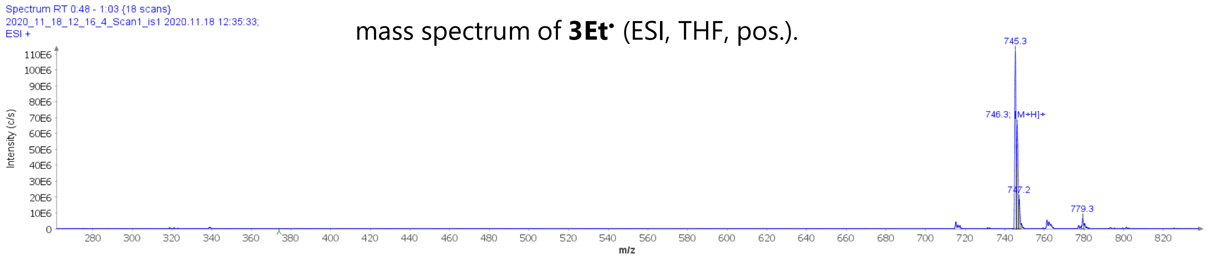

Figure S8: EPR and UV-vis spectrum of isolated **3Et˙** (EPR spectrum in THF @rt, UV-vis spectrum in toluene @rt).

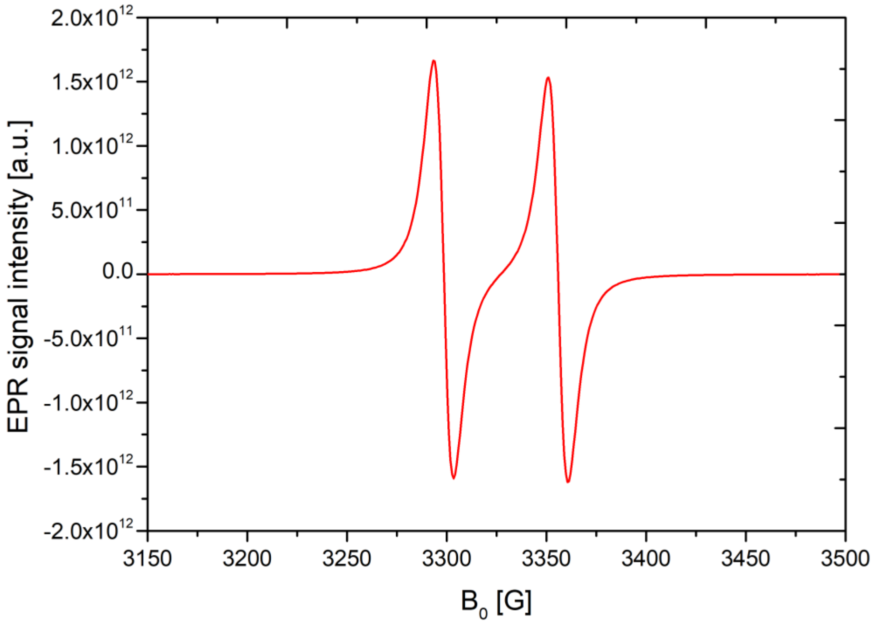

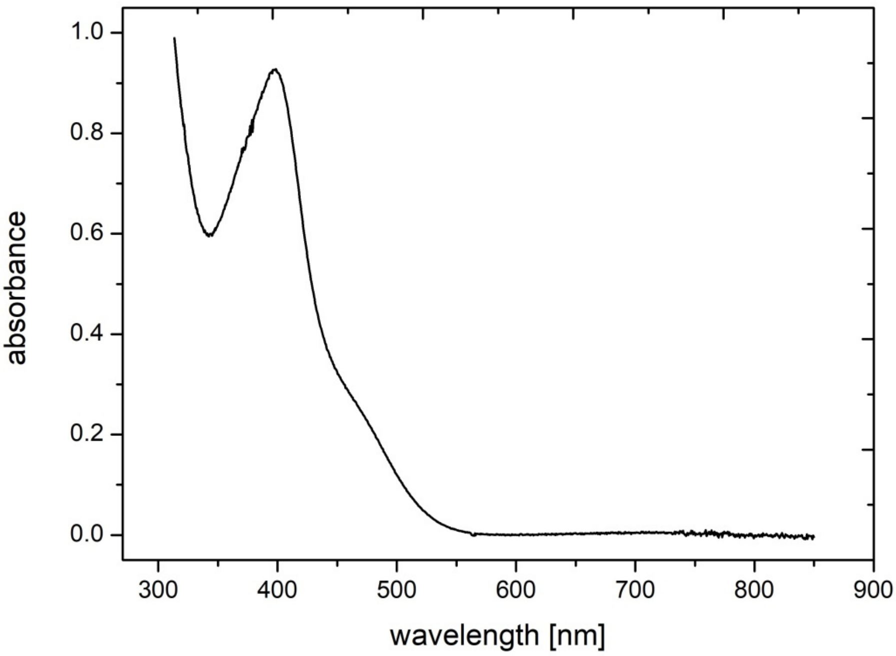

3.6 [BrP(μ-NTer)$_2$PBr] (4)

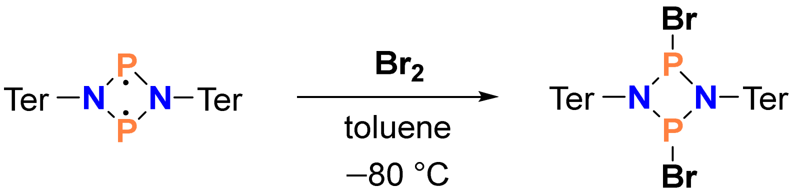

Br_2 (89 mg, 0.56 mmol) is slowly added to a solution of [$^{•}$P(μ-NTer)$_2$P$^{•}$] (400 mg, 0.56 mmol) in toluene (10 mL) at -80 °C with a microliter syringe. While heating to rt over the course of one hour, the the reaction mixture is stirred, and its colour slowly changes from orange to light yellow over the course of 10 minutes. Afterwards, the reaction mixture is stirred for another hour and then all volatile components are removed *in vacuo* (1×10^{-3} mbar) at 50 °C (water bath). The residue is dissolved in fresh toluene. The clear solution is concentrated *in vacuo* (1×10^{-3} mbar) to incipient crystallization and stored overnight at ambient temperature. After removal of the supernatant colourless crystals are obtained. Crystallization results in almost pure *trans* product (97% *trans* isomer, 3% *cis* isomer). In the reaction *cis* and *trans* product are formed in a ratio of 2:7. **Yield** (97% pure *trans,* 2 fractions): 289 mg (0.33 mmol, 59 %). It was so far not possible to also crystallize the *cis* isomer in pure form.

All data for almost pure *trans* isomer, except for ^{31}P NMR shifts also for *cis* isomer.

Mp. 256 °C. **CHN** calcd. (found) in %: C 65.76 (65.28), H 5.75 (5.48), N 3.20 (3.02). **^{31}P{^{1}H} NMR** (C_6D_6, 202.5 MHz): δ = 243.9 (s, 2 P, *P*-Br, *cis*), 277.9 (s, 2 P, *P*-Br, *trans*). **^{1}H NMR** (C_6D_6, 250.1 MHz): δ = 2.15 (s, 12 H, *p/o*-C*H*$_3$), 2.19 (s, 12 H, p/*o*-C*H*$_3$), 2.33 (s, 12 H, p/*o*-C*H*$_3$), 6.55-6.85 (m, 14H, H-arom.). **^{13}C{^{1}H}** NMR (C_6D_6, 63.0 MHz): δ = 22.0 (br s, *p/o*-CH$_3$), 22.8 (t, *p/o*-CH$_3$, *J* (^{13}C-^{31}P) = 2.75 Hz), 125.1 (s, CH (arom.)), 129.2 (s, CH (arom.)), 129.6 (s, CH (arom.)), 132.1 (s, CH (arom.)), 134.4 (s, *C* (arom.)), 135.3 (m, *C* (arom.)), 138.0 (s, *C* (arom.)), 138.5 (m, *C* (arom.)). **IR** (ATR, 32 scans, cm^{-1}): $\tilde{\nu}$ = 2853 (w), 2728 (vw), 1610 (w), 1569 (w), 1482 (w), 1406 (s), 1377 (m), 1220 (s), 1189 (w), 1101 (w), 1084 (m), 1031 (w), 1004 (w), 954 (vw), 905 (s), 882 (vs), 843 (vs), 796 (s), 765 (w), 750 (s), 701 (m), 645 (w), 589 (w), 573 (w), 540 (m), 501 (w), 489 (w), 474 (w), 455 (w), 439 (m), 427

(s). **Raman** (633 nm, 15 s, 20 scans, cm^{-1}): $\tilde{\nu}$ =3014 (1), 2917 (1), 2857 (1), 2731 (1),1612 (1), 1577 (1), 1479 (1), 1427 (1),1379 (1),1373 (1),1302 (3),1281 (1),1274 (1), 1243 (1), 1162 (1), 1102 (1), 1090 (1), 1003 (1), 943 (1), 845 (1), 818 (1), 734 (1), 704 (1), 587 (1), 573 (4), 558 (2), 538 (1), 526 (1), 521 (2), 511 (1), 485 (1), 468 (1), 368 (1), 340 (10), 319 (2), 298 (1), 276 (1), 260 (1), 233 (1). **MS** (CI, pos., isobutane) *m/z*: 875 $[MH]^+$, 807 $[(TerNP)_2CH_2Ph]^+$, 797 $[(TerNP)_2Br]^+$, 795 $[(TerNP)_2Br]^+$, 716 $[(TerNP)_2]^+$, 358 $[TerNP]^+$.

Figure S9: NMR, IR and RAMAN spectra of the *trans* isomer of **4,** apart from ^{31}P NMR spectrum also including *cis* isomer (solvent signals are marked by an asterisk).

^{1}H NMR spectrum (*trans* isomer, C_6D_6)

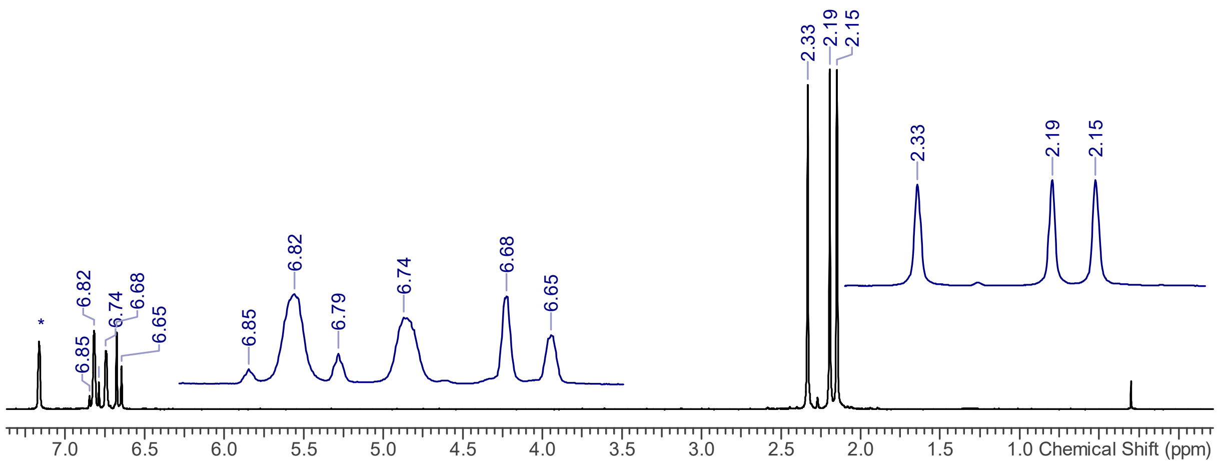

Figure S9 continued.

^{13}C NMR spectrum (*trans* isomer, C_6D_6)

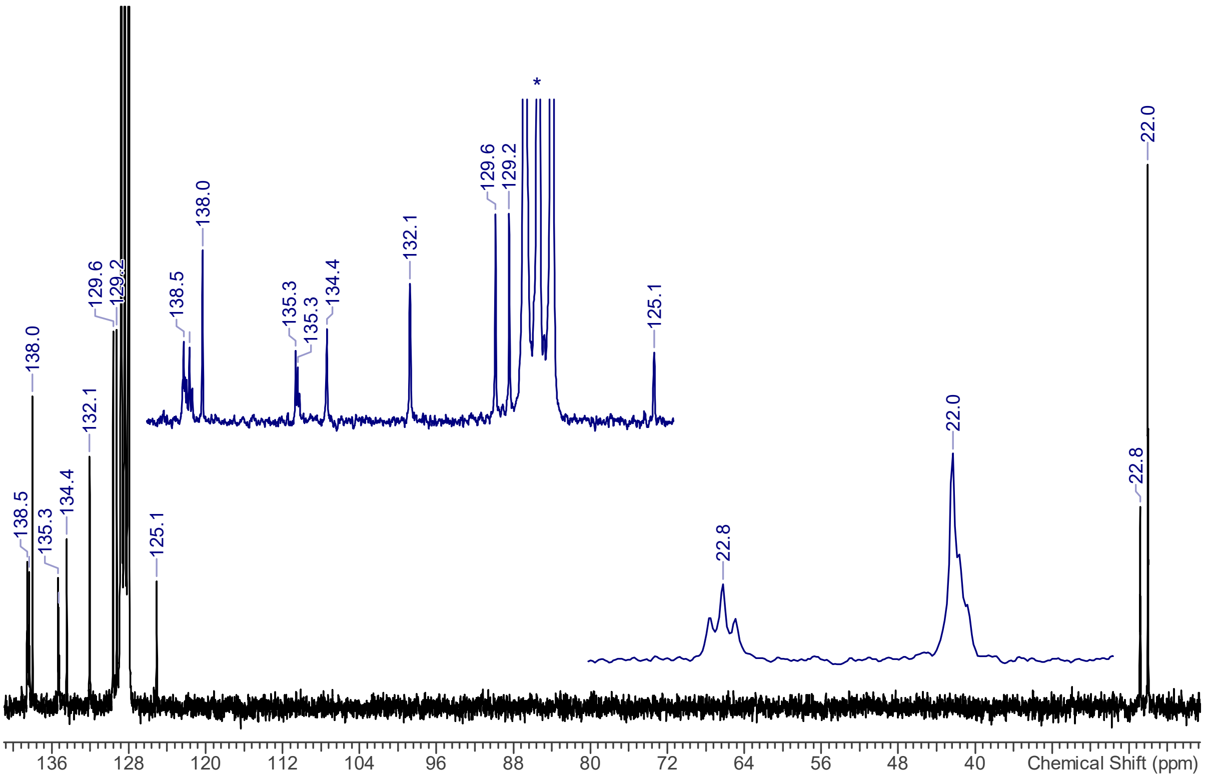

^{31}P{^{1}H} NMR spectrum before crystallization (mixture, C_6D_6)

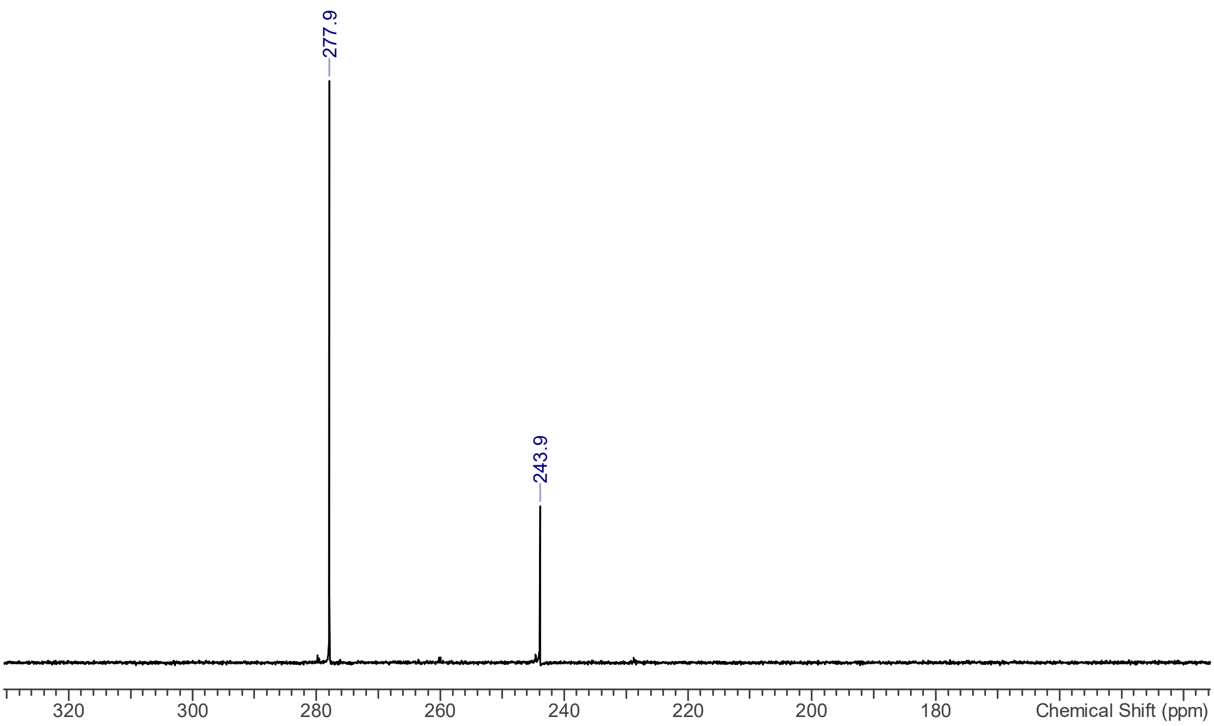

Figure S9 continued.

$^{31}P\{^1H\}$ NMR spectrum after crystallization, *trans* isomer (C_6D_6)

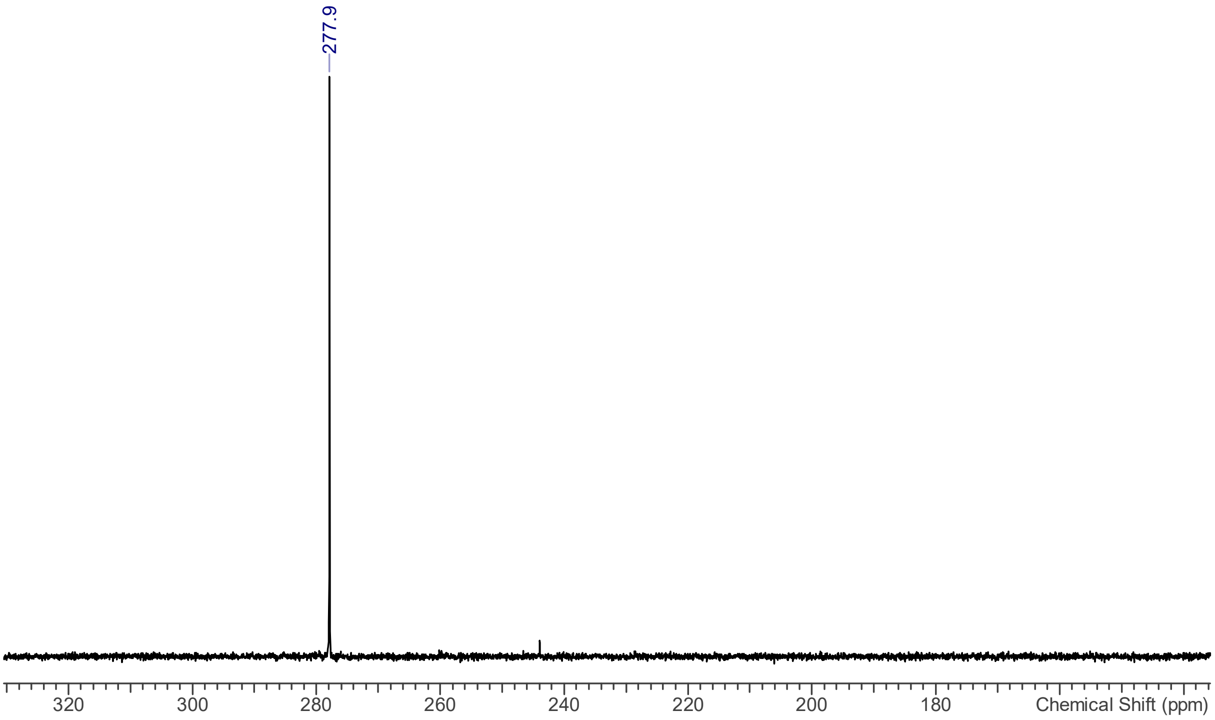

Figure S9 continued.

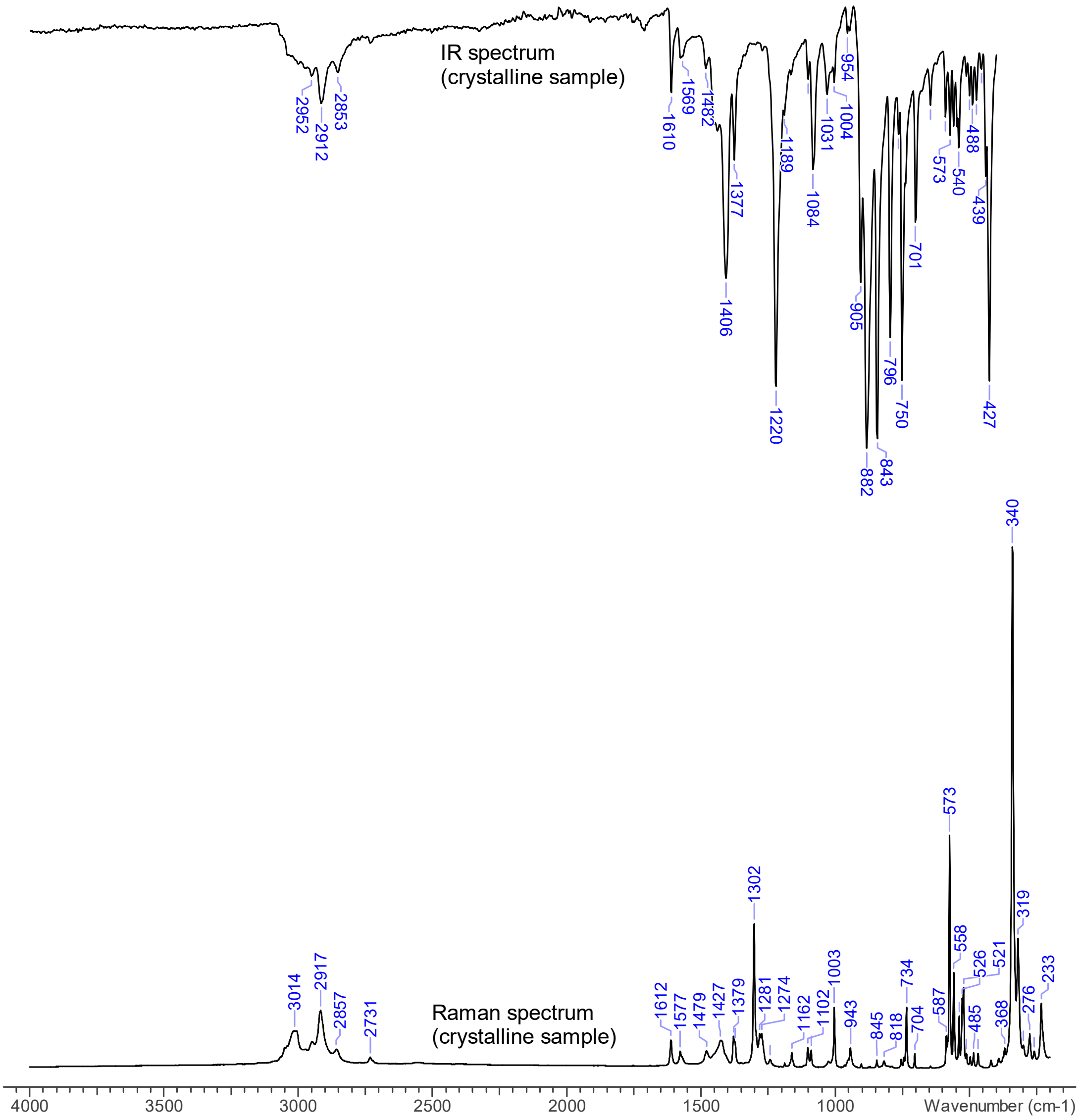

4 Additional spectroscopic details

4.1 Reaction kinetics bromoalkane addition (EtBr)

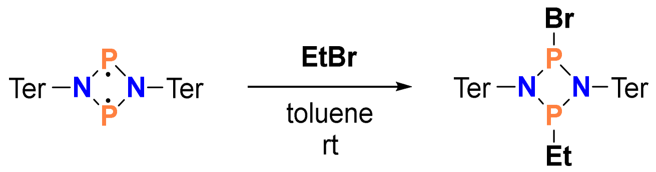

The reaction above was traced by ^{31}P NMR spectroscopy. Spectra were recorded over several days in shorter intervals at the beginning and longer ones towards the end of the experiment. The reaction rate was determined by modelling second order kinetics to the experimental data using Origin.

Figure S10: Kinetics of the reaction modelled to ^{31}P-NMR spectroscopic data as second order kinetics.

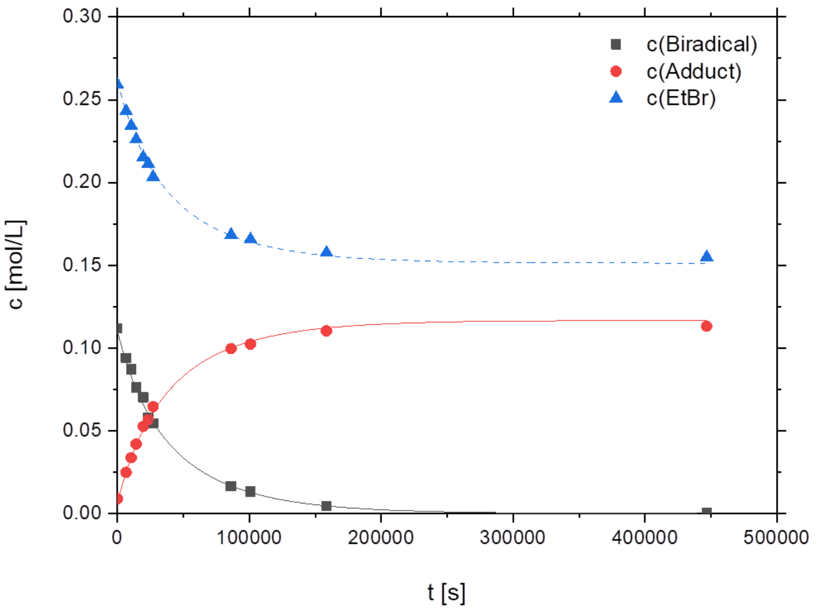

$[Bir]_0$ = 0.110(1) mol/L

$[EtBr]_0$ = 0.261(1) mol/L

$[Add]_0$ = 0.007(1) mol/L

$k = 1.11(3) \times 10^{-4}$ L/(mol s)

During the addition of bromoethane to **1** an *in situ* EPR spectrum was recorded indicating the presence of a phosphorus-centred radical intermediate.

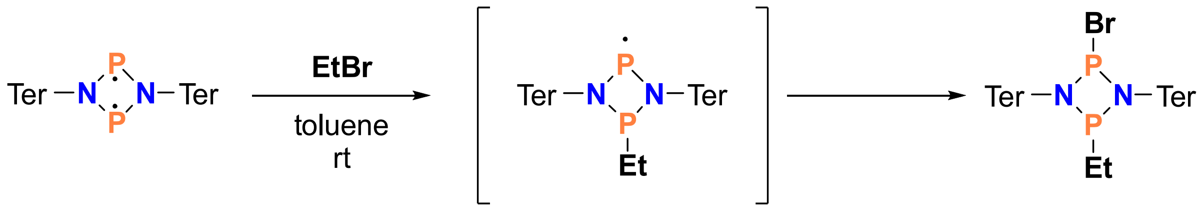

Figure S11: In situ EPR spectrum during formation of **2a** (toluene, rt).

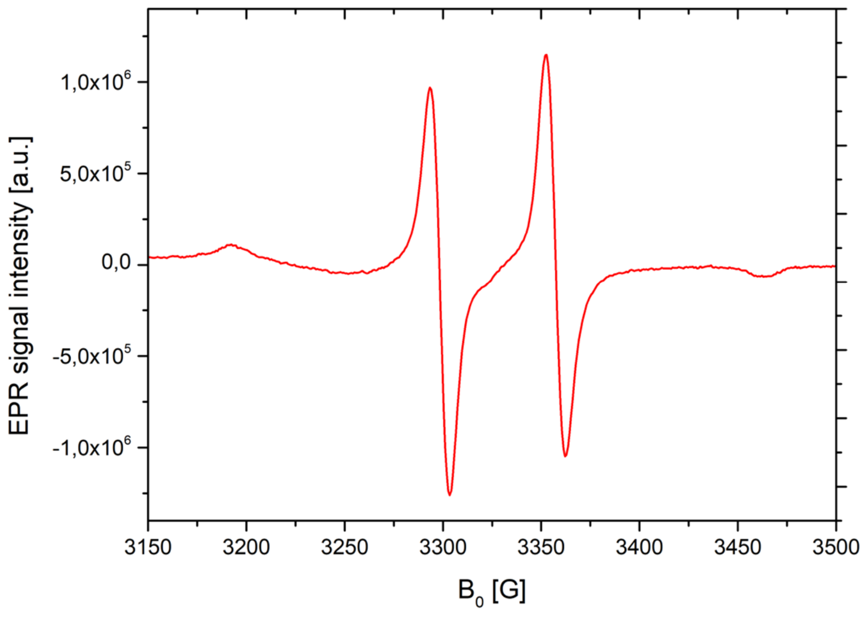

4.2 Addition of AIBN to the EtBr addition reaction

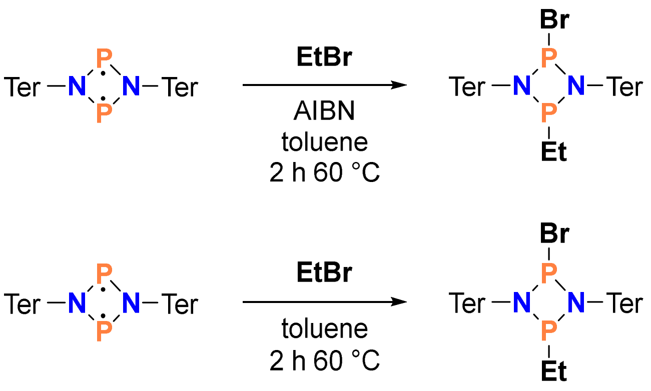

Figure S12: ^{31}P NMR spectra of the addition reaction with (top) and without (bottom) the addition of AIBN at the same relative time, ^{31}P{^{1}H} NMR (toluene-*d8*, 121.5 MHz) δ = 276.2 ([$^{\bullet}$P(μ-NTer)$_2$P$^{\bullet}$]), 255.1 ([BrP(μ-NTer)$_2$PEt]), 229.7 ([BrP(μ-NTer)$_2$PEt]), reaction @60°C, measurement @rt).

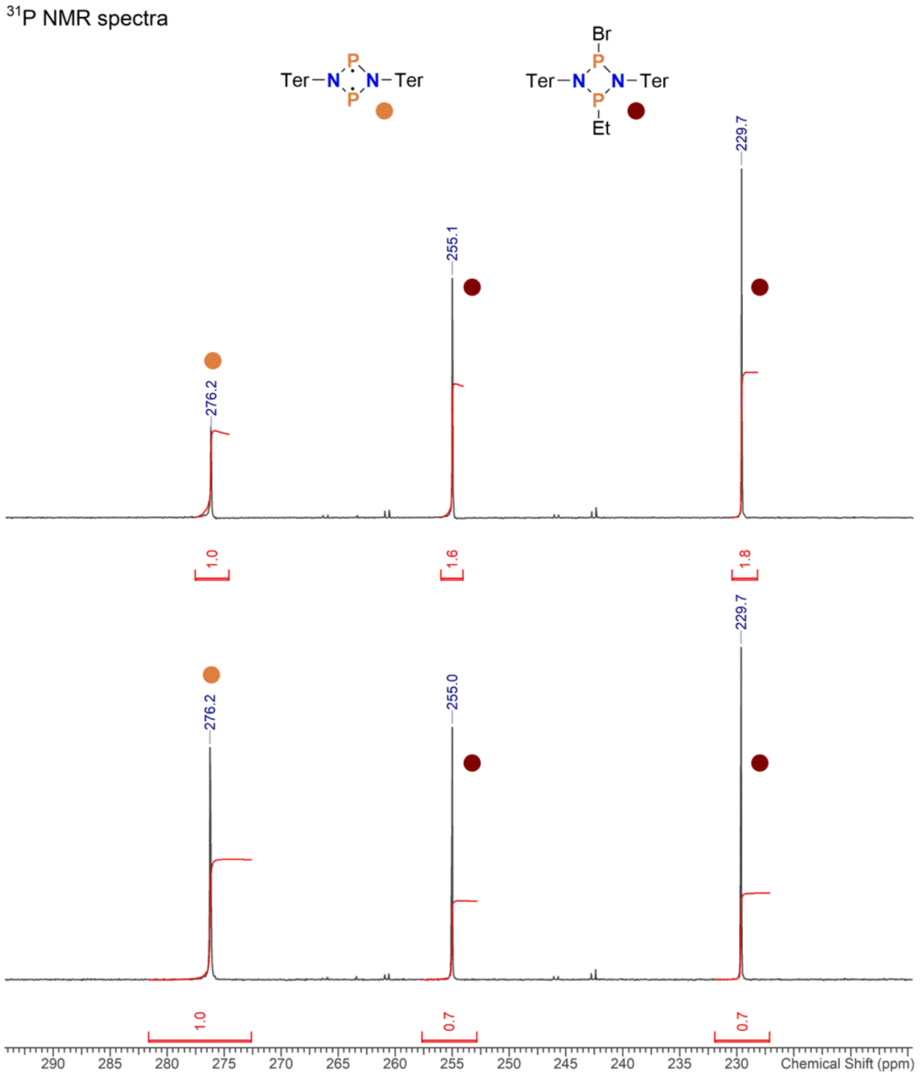

The reaction was repeated with and without the addition of AIBN. Reaction temperature was 60°C to activate the AIBN. After 2 h ^{31}P NMR spectra were recorded. The ratio of biradical (starting material) to addition product was 2:7 with AIBN and 1:1.4 without AIBN (Figure S12). With the additional radical starter the reaction was accelerated considerably indicating a radical reaction mechanism.

In order to determine the molecular pathway by which AIBN accelerates the reaction it was tried to react **1** (^{31}P NMR shift: 276.2 ppm) directly with AIBN under thermal activation (60°C) (Figure S13). No reaction was observed, therefore the accelerative effect of AIBN is attributed to increased formation of $Et^{\bullet}/Br^{\bullet}$ radicals.

Figure S13: $^{31}P\{^{1}H\}$ NMR spectrum of the reaction of **1** with AIBN (toluene-*d8*, reaction @60°C, measurement @rt).

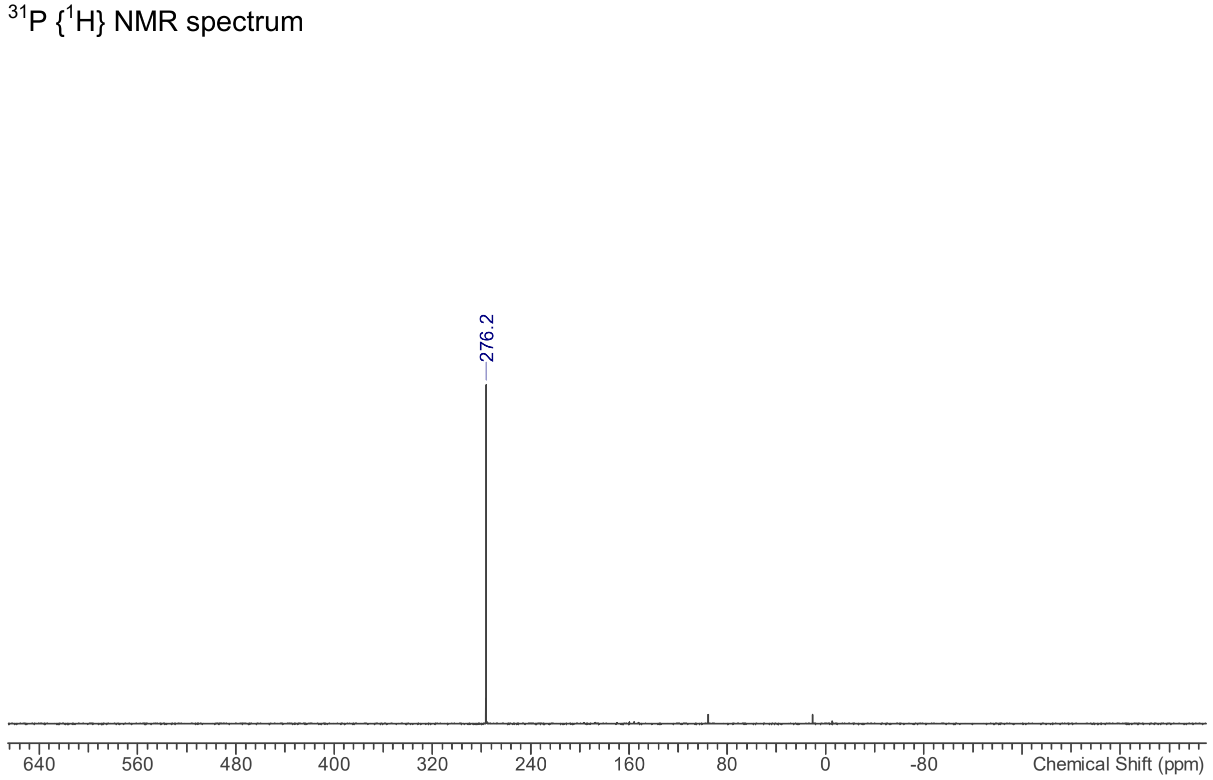

4.3 Reaction of 3Et• with EtBr

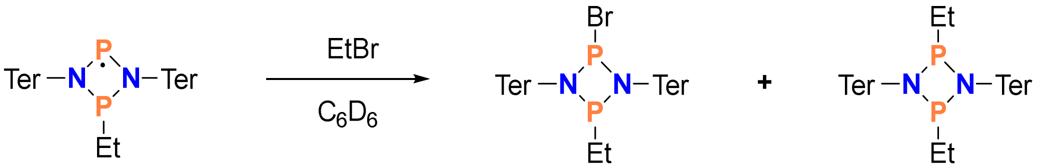

To better understand the mechanism of the EtBr addition to **1**, EtBr (5.8 mg, 0.054 mmol) was added to a solution of **3Et•** (40 mg, 0.054 mmol) in C_6D_6 in an NMR tube with a microliter syringe at ambient temperature. The colour of the solution changed from dark red to light yellow upon mixing. During the reaction a 1:1 mixture of *trans*-**2a** (229.2 ppm and 255.2 ppm in the ^{31}P NMR spectrum) and two other species (225.2 ppm and 266.7 ppm) were formed. We assigned the additional two signals to *cis*- and *trans*- [EtP(μ-NTer)$_2$PEt] (**5a**) as a coupling/broadening is visible in the ^{31}P ^{1}H-coupled NMR spectra which disappears in the ^{31}P{^{1}H} spectrum. Also, the formation of a 1:1 mixture of [BrP(μ-NTer)$_2$PEt] (**2a**) and [EtP(μ-NTer)$_2$PEt] (**5a**) when a stoichiometric amount of EtBr is used is in line with our computational results on the radical reaction mechanism (see Table S6 for reaction energies). For every equivalent of [BrP(μ-NTer)$_2$PEt] that is formed when **3Et•** reacts with EtBr, one equivalent of Et• is formed that can react with another equivalent of **3Et•** forming one equivalent of [EtP(μ-NTer)$_2$PEt] (**5a**).

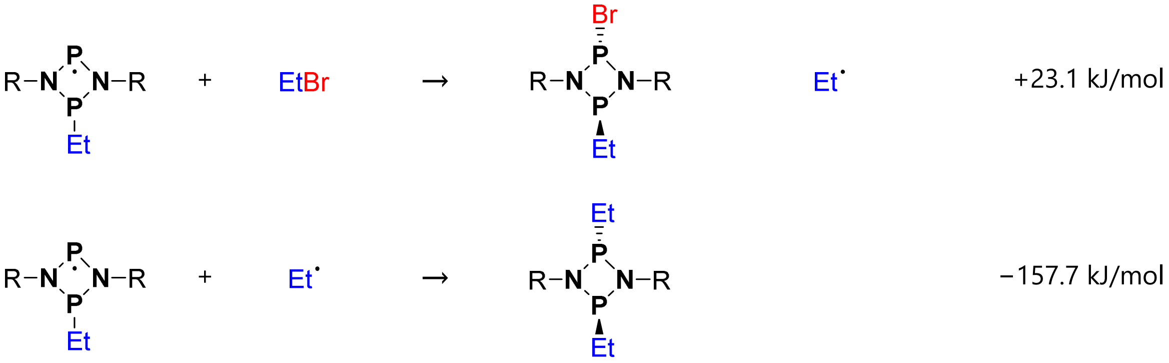

When an excess of EtBr is used (Figure S15), the integral ratio of the signals is no longer close to 1:1. That can be explained by the reaction of Et• radicals with EtBr (forming Br•)

becomes more likely than a reaction of Et• with **3Et•** when a large excess of EtBr is present. Br• radicals then can react with **3Et•** forming **2a.**

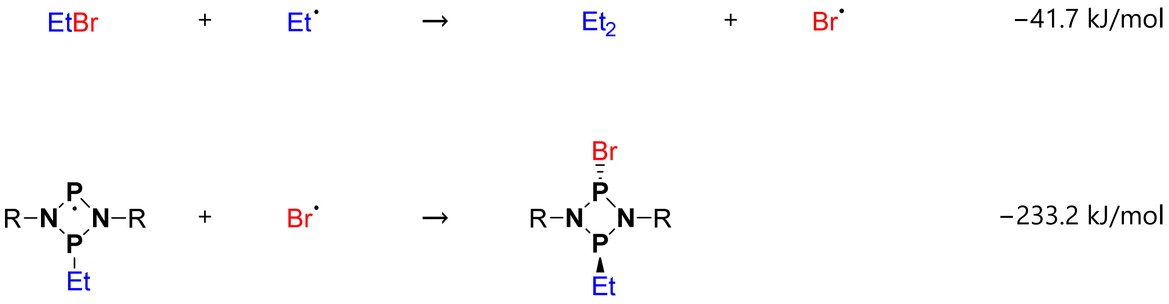

Furthermore, after 3 days reaction time with a large excess of EtBr, a decrease in concentration (relative to **2a**) of the two [EtP(µ-NTer)$_2$PEt] (**5a**) signals is observed (Figure S16). This is also in accordance with the calculated radical pathways (see Table S6). When there are radicals and a large excess of EtBr present, a stepwise reaction of [EtP(µ-NTer)$_2$PEt] (**5a**) with EtBr to form **2a** is thermodynamically favoured and overtime the thermodynamic reaction product is formed, leading to the relative decrease of the [EtP(µ-NTer)$_2$PEt] (**5a**) signal in the ^{31}P NMR spectrum.

Figure S14: ^{1}H and ^{31}P NMR data of the reaction of **3Et˙** with EtBr.

^{1}H NMR spectrum (C_6D_6)

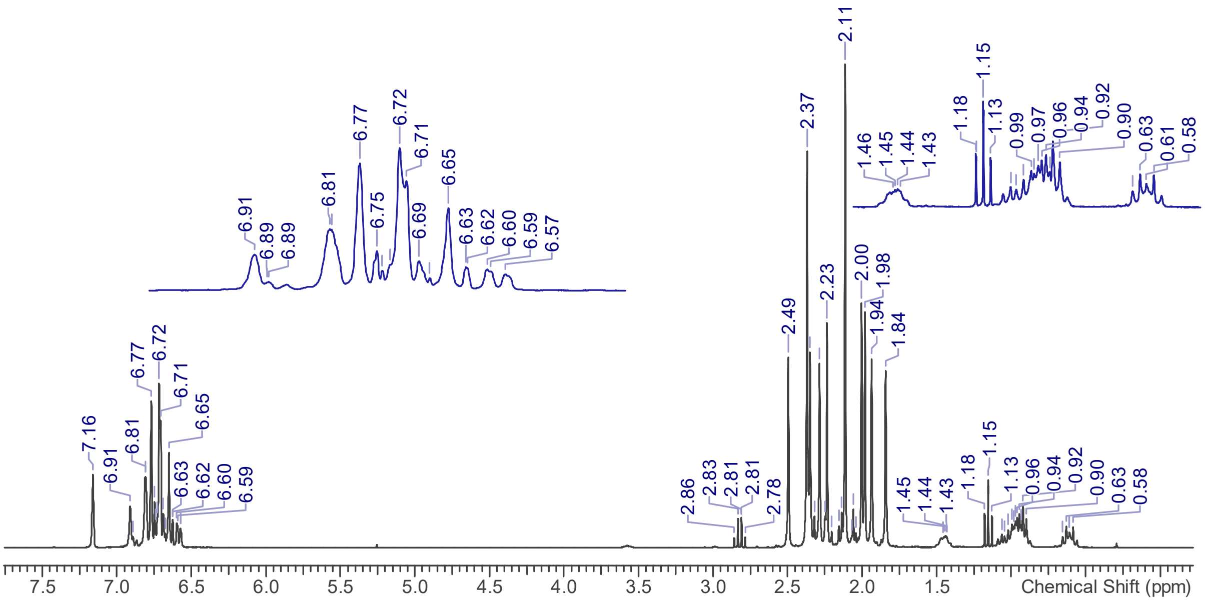

^{31}P NMR spectrum (C_6D_6)

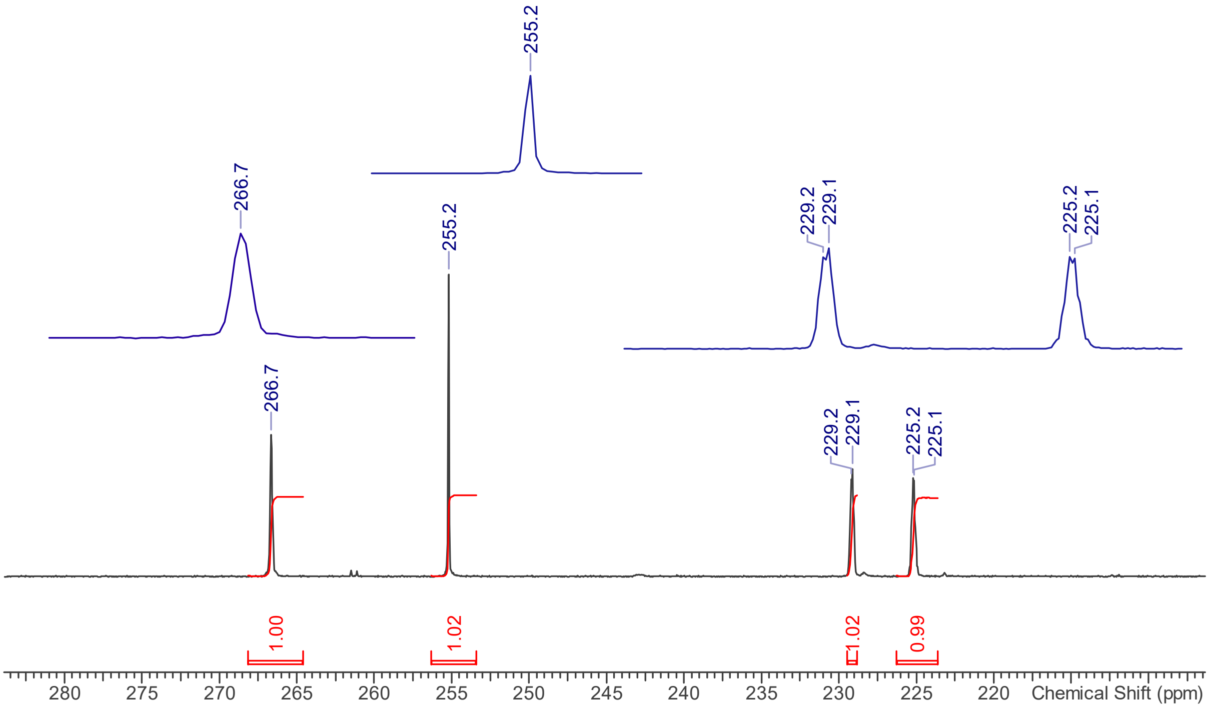

Figure S14 continued.

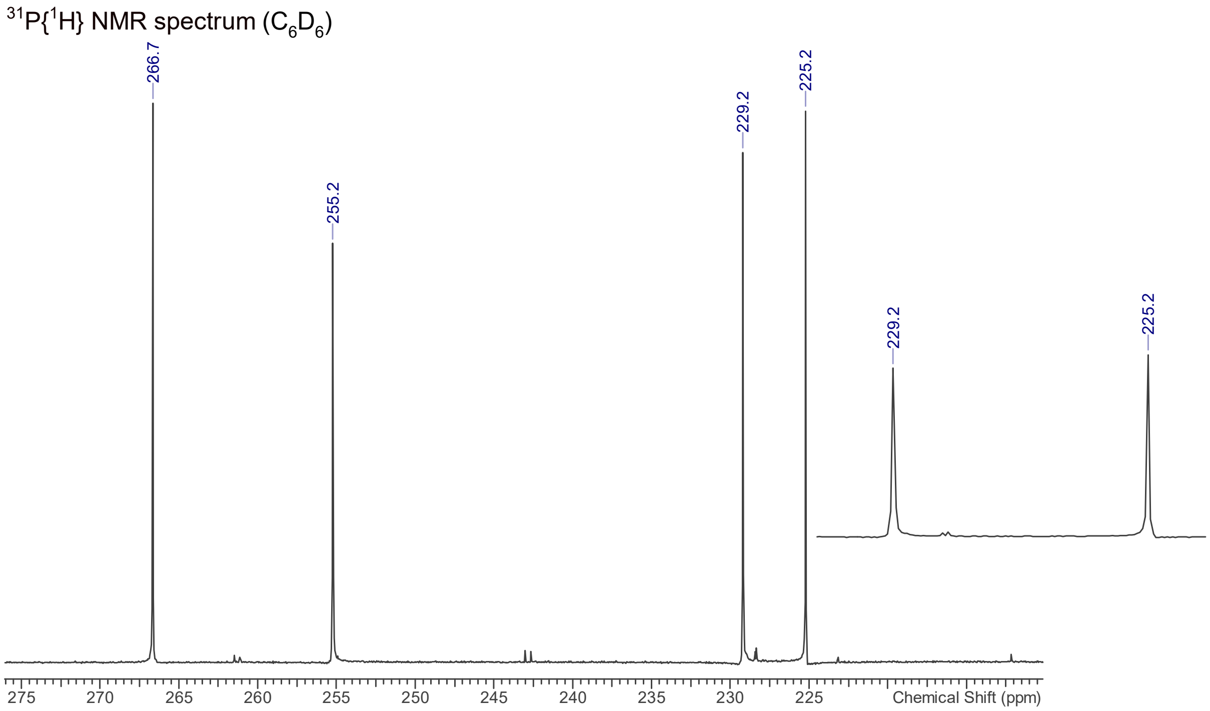

Figure S15: ^{31}P NMR spectrum of the reaction of **3Et**$^{\bullet}$ with EtBr (excess).

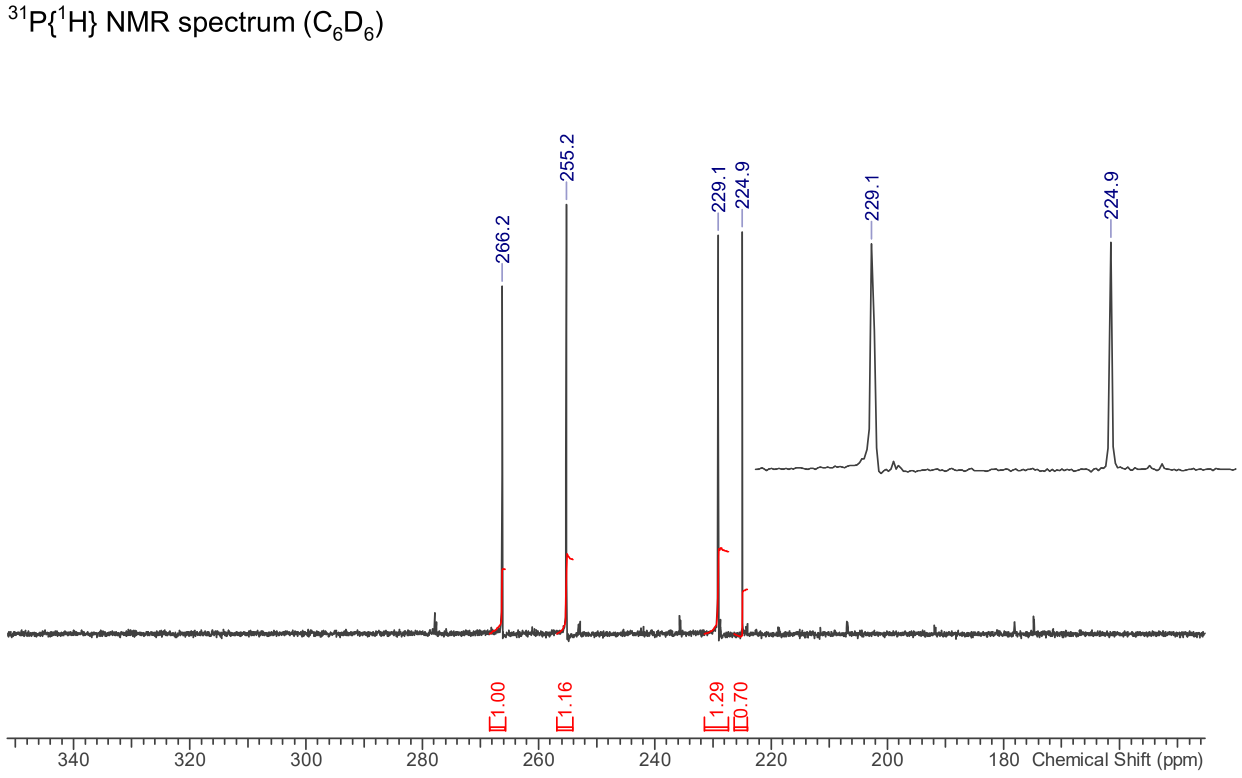

Figure S16: ^{31}P NMR spectrum of the reaction of **3Et**$^{\bullet}$ with EtBr (excess) after 3 days reaction time.

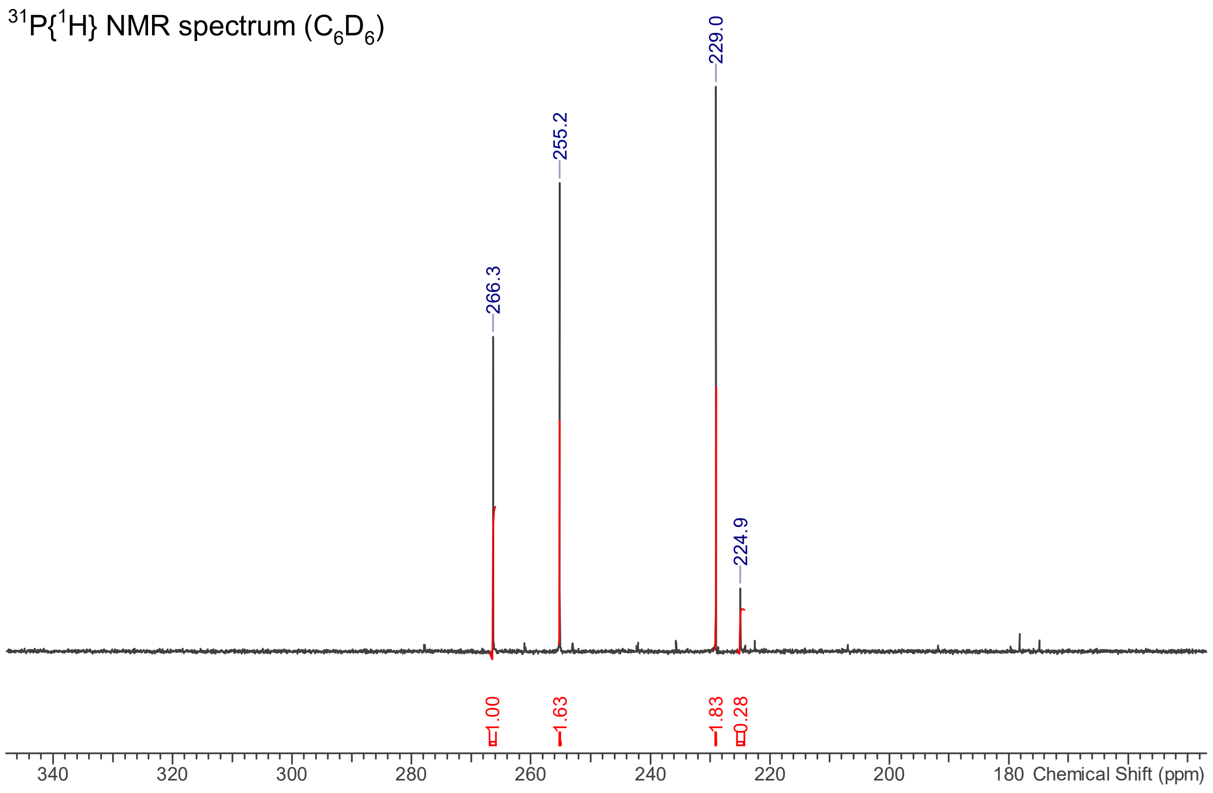

4.4 Reaction of 3Et˙ with Br_2

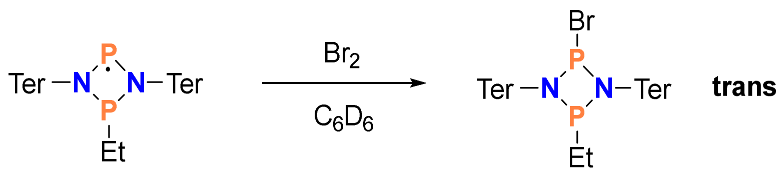

Dry bromine (8.6 mg, 0.054 mmol) is added to a solution of **3Et˙** (40 mg, 0.054 mmol) in C_6D_6 in an NMR tube with a microliter syringe at ambient temperature. The colour of the solution changes from dark red to yellow upon mixing. During the reaction *trans*-**2a** (229.2 ppm and 255.2 ppm in the ^{31}P NMR spectrum) is formed. *cis*-**2a** is not detected. Other signals indicate a partial decomposition of **3Et˙** due to oxidation by an excess of bromine; however, the decomposition products could not be identified so far.

Figure S17: NMR spectra of the reaction of **3Et˙** with Br_2.

$^{31}P\{^1H\}$ NMR spectrum (C_6D_6)

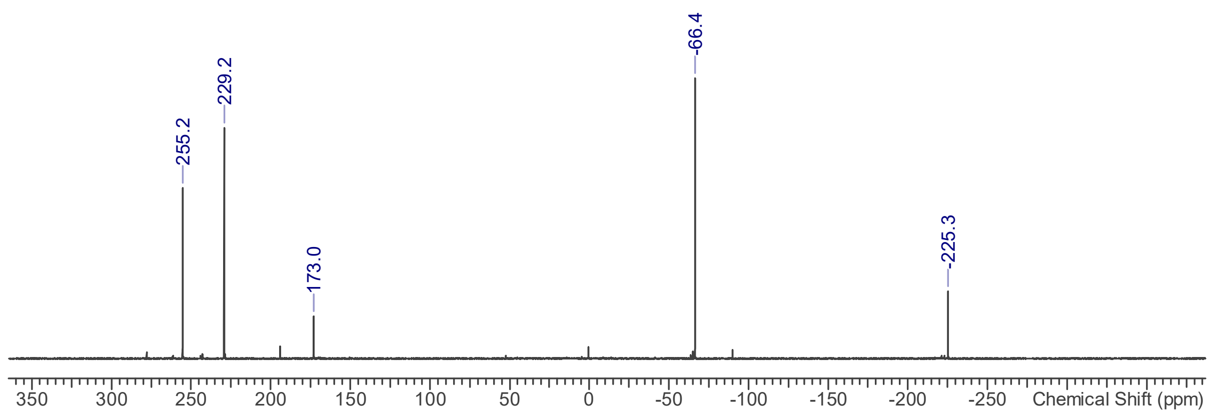

^{31}P NMR spectrum (C_6D_6)

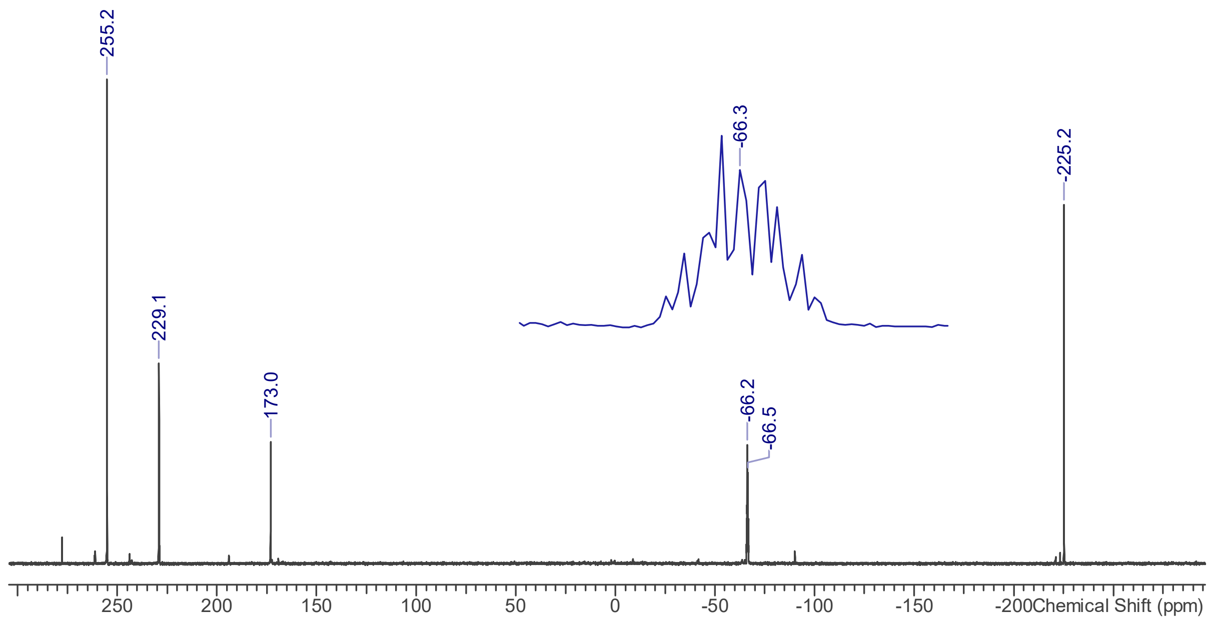

5 Computational details

5.1 General remarks

Computations were carried out using Gaussian09,[6] ORCA 4.2.1,[7,8] Multiwfn 3.7[9] and the standalone version of NBO 6.0.[10–13]

Unless otherwise noted, **structure optimizations** were performed using Gaussian09 and employed the pure exchange-correlation functional PBE[14,15] in conjunction with Grimme's dispersion correction D3(BJ)[16,17] and the def2-TZVP basis set[18] (notation PBE-D3/def2-TZVP). The resolution-of-identity (RI) approximation was applied, using Weigend's accurate Coulomb-fitting basis set.[19] Numerical integration of the exchange-correlation energy was done on Gaussian's "ultrafine" grid. The stability of the all Kohn-Sham wavefunctions was analysed, and the unrestricted "broken

symmetry" solution was used where appropriate. All structures were fully optimized and confirmed as minima by frequency analyses.

Chemical shifts and **coupling constants** were derived by the GIAO method.[20–24] The calculated absolute shifts ($\sigma_{calc,X}$) were referenced to the experimental absolute shift of 85% H_3PO_4 in the gas phase ($\sigma_{ref,1}$ = 328.35 ppm),[25] using PH_3 ($\sigma_{ref,2}$ = 594.45 ppm) as a secondary standard:[26]

$$\delta_{calc,X} = (\sigma_{ref,1} - \sigma_{ref,2}) - (\sigma_{calc,X} - \sigma_{calc,PH_3})$$

$$= \sigma_{calc,PH_3} - \sigma_{calc,X} - 266.1 \text{ ppm}$$

At the PBE-D3/def2-TZVP level of theory, σ_{calc,PH_3} amounts to + 563.04 ppm.

EPR data were calculated[27–30] using ORCA 4.2.1 at the RI-SOMF(1X)[29,31]/PBE0[14,15,32]-D3/def2-TZVP level of theory, using optimized structures at the PBE-D3/def2-TZVP level of theory (vide supra). The Coulomb terms of the hybrid functional as well as the spin-orbit coupling operator were approximated using the RI approximation, while the HF exchange term of the hybrid functional was treated using the Chain of Spheres (COSX) approximation (i.e., RIJCOSX).[33]

UV-vis absorption spectra were calculated with Gaussian09 using the TD-DFT method[34–36] at the PBE0-D3/def2-TZVP level of theory, using the optimized structures at the PBE-D3/def2-TZVP level of theory.

More **accurate electronic energies** for the optimized structures (vide supra) were computed using ORCA 4.2.1 by single-point DLPNO-CCSD(T)[37–40] calculations employing the def2-TZVP basis set[18] and def2-TZVP/C correlation fitting basis[41] (notation: DLPNO-CCSD(T)/def2-TZVP//PBE-D3/def2-TZVP). Thermodynamic quantities at this level of theory were calculated using the DLPNO-CCSD(T) single point energy and the thermal corrections at the PBE-D3/def2-TZVP level of theory. The T_1 diagnostic was evaluated in each case to ensure reliable results (empirically, CCSD(T) results are considered reliable if $T_1 < 0.02$).[42,43]

Ab-initio calculations using **multiconfigurational wavefunctions** were performed using ORCA 4.2.1. The Complete Active Space SCF (CASSCF) method was employed,[44–52] which correctly describes the multireference character of the investigated systems (i.e. treatment of non-dynamic correlation). To account for dynamic correlation, the CASSCF reference wavefunctions were subjected to multireference perturbation calculations, using the Fully Internally Contracted *N*-Electron Valence State Perturbation Theory (FIC-NEVPT2).[53–55]

Please note that all computations were carried out for single, isolated molecules in the gas phase (ideal gas approximation). There may well be significant differences between gas phase and condensed phase.

5.2 Mechanism of the Reaction [$^{\bullet}$P(μ-NTer)$_2$P$^{\bullet}$] + EtBr

5.2.1 Model system

To obtain an initial idea of possible reaction pathways, the model reaction

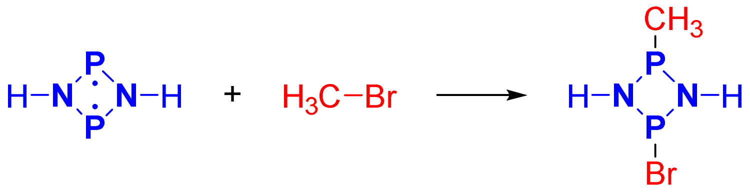

was investigated. Therefore, a variety of Nudged Elastic Band (NEB)[56–60] and relaxed potential energy surface (PES) scans were performed at the UPBE-D3/def2-SVP[18] level of theory using ORCA 4.2.1 or Gaussian09, respectively. Different orientations of the starting materials and configurations of the product were considered. It was not possible to locate a transition state for a concerted mechanism (e.g., analogous to the addition of H_2 to the singlet biradical [$^{\bullet}$P(μ-NTer)$_2$P$^{\bullet}$]).[61–63] All results pointed towards a stepwise (i.e. radical) mechanism of the reaction, in agreement with experimental observations.

In particular, our model computations implied that the formal abstraction of a Br radical (Br$^{\bullet}$) from the bromoalkane by the singlet biradical initiated the radical chain reaction (see also section 5.2.2). It was therefore of special interest to investigate this first reaction step in more detail. Thus, the **Minimum Energy Path** (MEP) on the singlet PES of the reaction

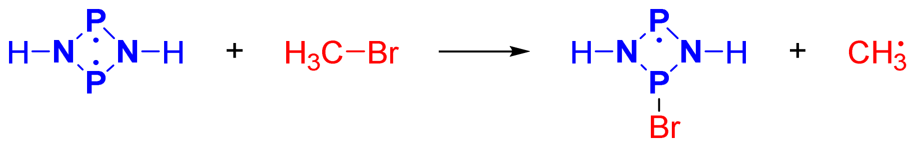

was computed at the FIC-NEVPT2/def2-TZVP//UPBE-D3/def2-TZVP as well as FIC-NEVPT2/def2-TZVP//CASSCF(4,4)/def2-TZVP levels of theory (Figure S18). The results of both approaches are similar. The biradical character increases smoothly as the reaction progresses, ultimately leading to two separate radical species (Figure S19).

Thus, this initiation reaction (resulting in a radical chain reaction, see below) is an intrinsically bi-radical process. Note that the tetraradical character, although low throughout the process, reaches a maximum at about d(C–Br) = 2.5 Å, where the C–Br and P–Br bond orders are roughly equal (Figure 9 in the manuscript), indicating a small admixture of a Lewis-type structure of the type $^{\bullet}$P(μ-NTer)$_2$P$^{\bullet}$···Br$^{\bullet}$···Me$^{\bullet}$.

Figure S18: Minimum Energy Path (MEP) of the reaction [$^{\bullet}$P(μ-NTer)$_2$P$^{\bullet}$] + MeBr → [P(μ-NTer)$_2$PBr]$^{\bullet}$ + Me$^{\bullet}$ plotted along the P–Br and C–Br distances. Blue curve: FIC-NEVPT2/def2-TZVP//UPBE-D3/def2-TZVP, grey curve: FIC-NEVPT2/def2-TZVP//CASSCF(4,4)/def2-TZVP.

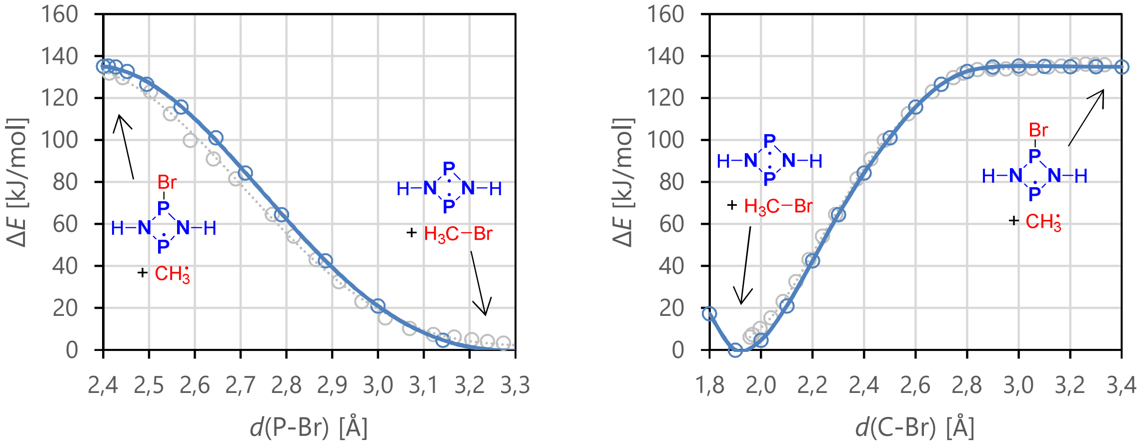

Figure S19: LUNO and LUNO+1 occupancy (bi- and tetraradical character, resp.) along the MEP in Figure S18, projected along the P–Br and C–Br distances. Blue curves: FIC-NEVPT2/def2-TZVP//UPBE-D3/def2-TZVP, grey curves: FIC-NEVPT2/def2-TZVP//CASSCF(4,4)/def2-TZVP. Natural orbitals computed using the unrelaxed FIC-NEVPT2 density.

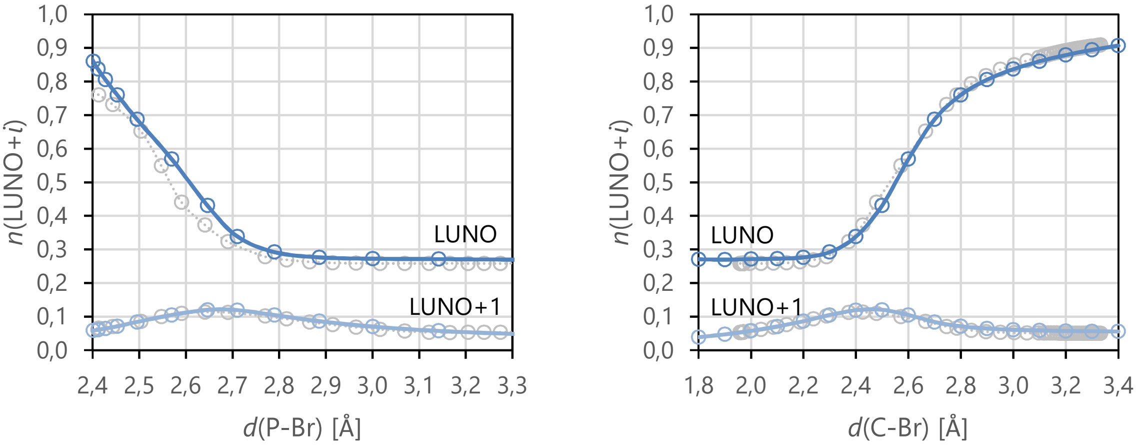

Figure S20: Minimum Energy Path (MEP) of the reaction [•P(μ-NTer)$_2$P•] + MeBr → [P(μ-NTer)$_2$PBr]• + Me• on the singlet (S_0, blue) and triplet (T_1, red) PES at the FIC-NEVPT2/def2-TZVP//UPBE-D3/def2-TZVP level of theory. The near degeneracy of singlet and triplet at the end of the reaction indicates two separate radicals.

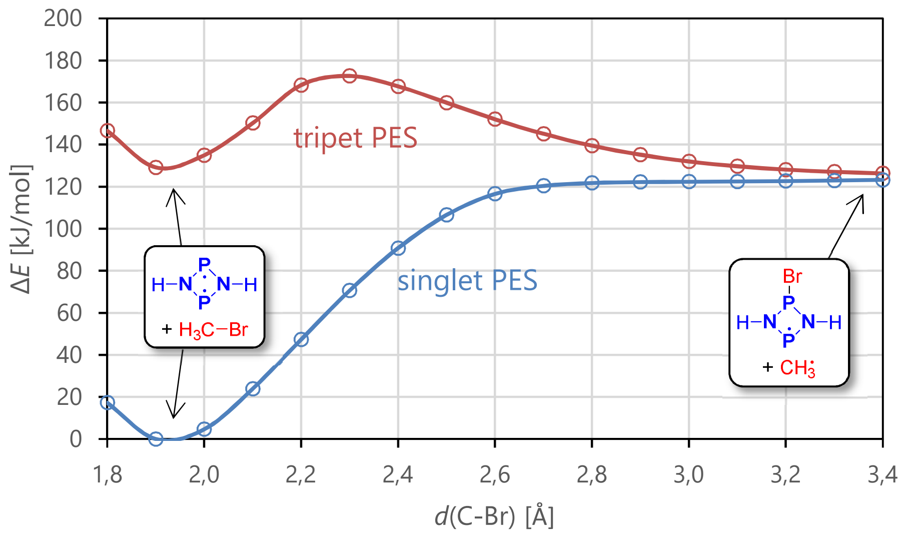

Bond orders along the MEP (Figure 9) were estimated using the CASSCF density matrices after Foster-Boys localization[64,65] of the orbitals in the active space.

The **local nondynamic correlation function**[66,67] (Figure 8) was also derived from the CASSCF wavefunction, however using the natural orbitals (i.e., after diagonalization of the CASSCF density matrix). The grid data for the local nondynamic correlation function was calculated using Multiwfn, and the isosurface was rendered using Avogadro.[68] Computation of the Fractional Occupation Number Weighted Electron Density (FOD)[69] in ORCA 4.2.1 using default settings (TPSS/def2-TZVP with a Fermi distribution at 5000 K) led to similar results.

Table S5: Active orbitals of the CASSCF(4,4)/def2-TZVP reference wavefunction, plotted at selected points along the MEP in Figure S18. Orbital occupation numbers given in brackets. Configurations listed for c_i^2 > 0.003.

d(C–Br)	HONO−1	HONO	LUNO	LUNO+1	[config.] c_i^2
1.8 Å	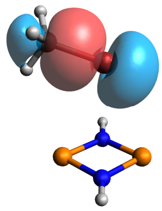<br>(1.98)	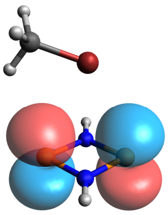<br>(1.76)	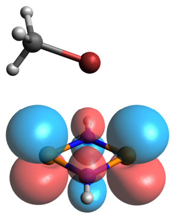<br>(0.24)	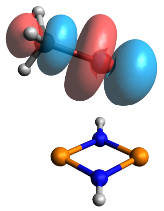<br>(0.02)	[2200] 0.869<br>[2020] 0.119<br>[0202] 0.010
2.5 Å	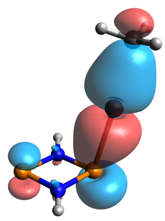<br>(1.91)	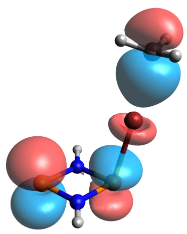<br>(1.58)	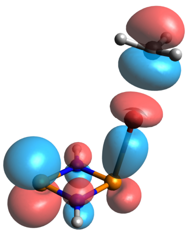<br>(0.42)	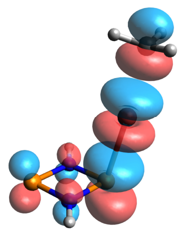<br>(0.09)	[2200] 0.751<br>[2020] 0.173<br>[1111] 0.058<br>[0202] 0.010<br>[0022] 0.004
3.4 Å	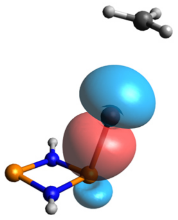<br>(1.97)	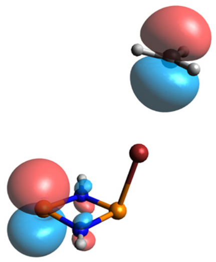<br>(1.09)	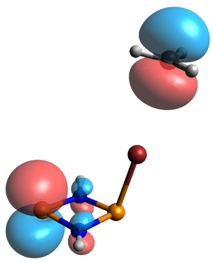<br>(0.91)	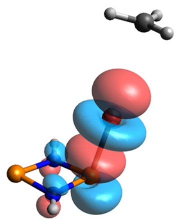<br>(0.03)	[2200] 0.536<br>[2020] 0.448<br>[0202] 0.008<br>[0022] 0.007

5.2.2 Actual molecules

Possible radical reaction steps were computed using the actual molecular structures (i.e., including the Ter and Et substituents, Table S6). In agreement with our computations on the model system (cf. section 5.2.1), we identified the abstraction of Br• from EtBr by the biradical [•P(μ-NTer)$_2$P•] as the probable initiation reaction. The actual reaction turnover proceeds via addition of an Et radical (which is generated by the initiation reaction) to the biradical [•P(μ-NTer)$_2$P•], leading to the intermediate [•P(μ-NTer)$_2$PEt], which was also observed spectroscopically. This then reacts with EtBr to the product [BrP(μ-NTer)$_2$PEt] and a new Et radical (Scheme S1).

Table S6. Computed radical reaction steps at the DLPNO-CCSD(T)/def2-TZVP//PBE-D3/def2-TZVP level of theory. The most likely reactions (based on $\Delta_R G°$ and concentrations) in each group are highlighted.

Reaction	$\Delta_R G°$ [kJ/mol]
Initiation reactions	
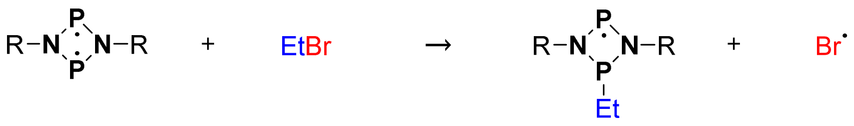	+149.7
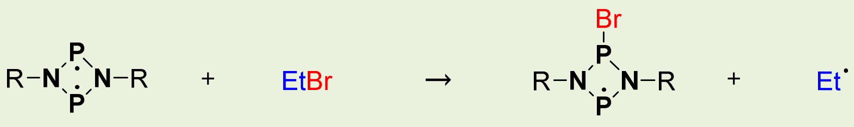	+103.0
EtBr → Et• + Br•	+256.4
Chain propagation	
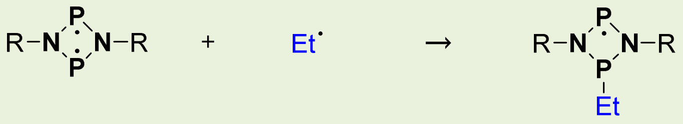	−106.6
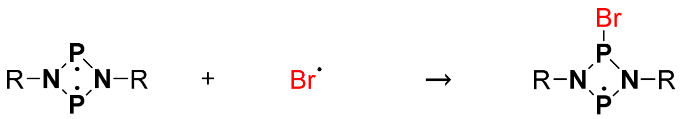	−153.3
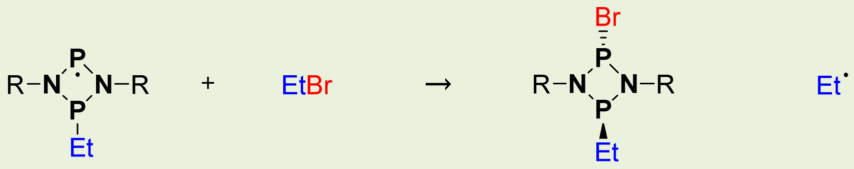	+23.1
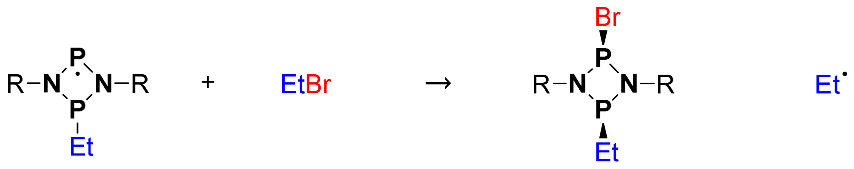	+47.4
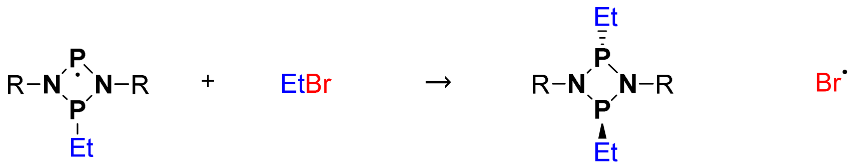	+98.6

Table S6 continued.

Reaction							$\Delta_R G°$ [kJ/mol]
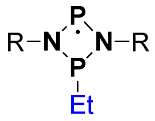	+	Et_2	→	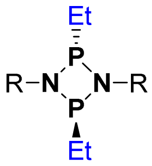		$Et^{\bullet}$	+140.3
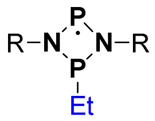	+	Br_2	→	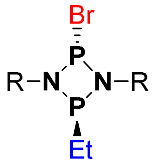		$Br^{\bullet}$	−67.1
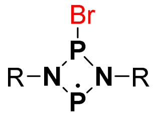	+	EtBr	→	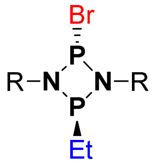	+	$Br^{\bullet}$	+69.9
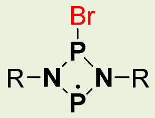	+	EtBr	→	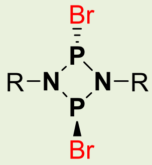	+	$Et^{\bullet}$	+31.9
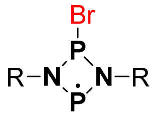	+	Et_2	→	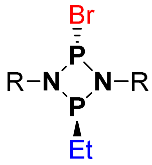	+	$Et^{\bullet}$	+111.6
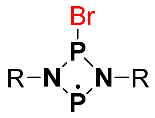	+	Br_2	→	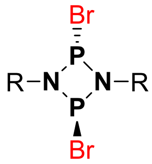	+	$Br^{\bullet}$	−58.3
EtBr	+	$Et^{\bullet}$	→	Et_2	+	$Br^{\bullet}$	−41.7
EtBr	+	$Br^{\bullet}$	→	Br_2	+	$Et^{\bullet}$	+90.2

Table S6 continued.

Reaction					$\Delta_R G°$ [kJ/mol]
Termination reactions					
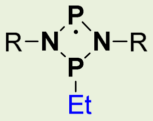	+	$Et^{\bullet}$	→	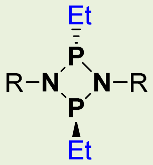	−157.7
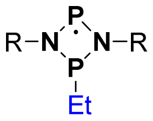	+	$Br^{\bullet}$	→	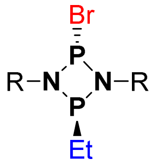	−233.2
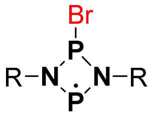	+	$Et^{\bullet}$	→	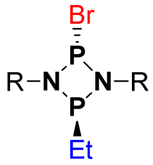	−186.5
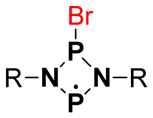	+	$Br^{\bullet}$	→	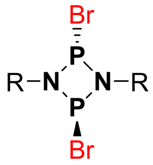	−224.4
$Et^{\bullet}$	+	$Et^{\bullet}$	→	Et_2	−298.0
$Br^{\bullet}$	+	$Br^{\bullet}$	→	Br_2	−166.1
Overall reaction					
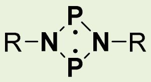	+	EtBr	→	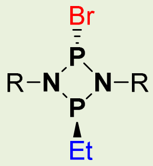	−83.5
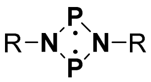	+	EtBr	→	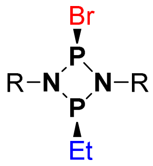	−59.3

Table S6 continued.

Reaction					$\Delta_R G°$ [kJ/mol]
Further possible side reactions					
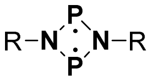	+	Et_2	→	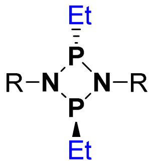	+33.7
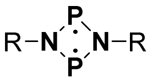	+	Br_2	→	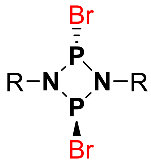	−211.7

Scheme S1. Schematic depiction of the radical reaction mechanism. Free reaction energies ($\Delta_R G°$, $p°$ = 1 atm) in kJ/mol (DLPNO-CCSD(T)/def2-TZVP//PBE-D3/def2-TZVP).

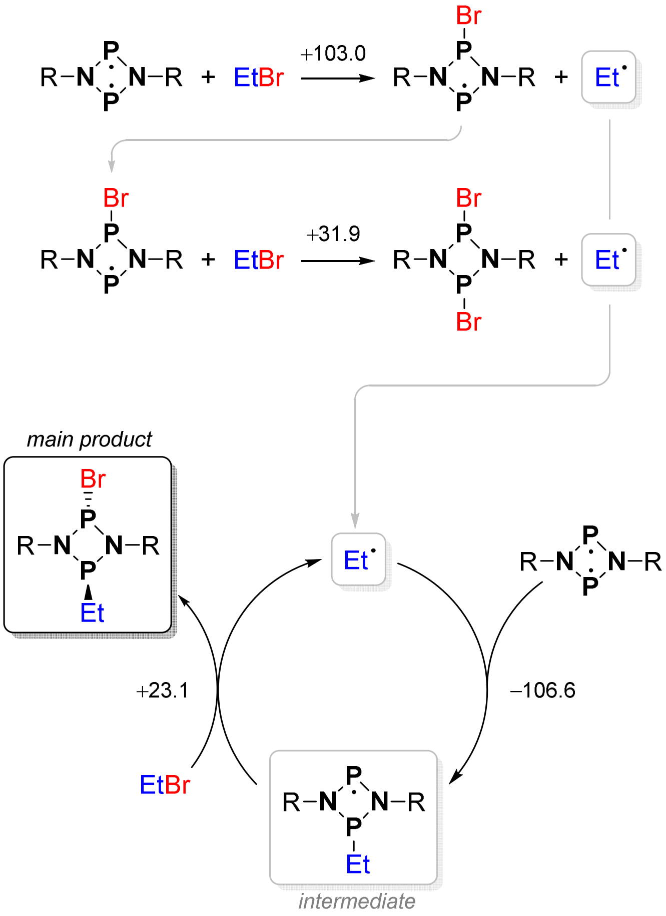

5.3 Summary of calculated data

Table S7. Summary of calculated data. All energies in atomic units.

Compd.	PG	PBE-D3			DLPNO-CCSD(T)		
		⟨S^2⟩	E_{tot}	ΔG[a]	⟨S^2⟩	$E_{CCSD(T)}$[c]	T_1
[$^{\bullet}$P(μ-NTer)$_2$PEt] *endo*	C_1	0.754	−2728.6479	+0.7921	0.750	−2725.9261	0.010
[$^{\bullet}$P(μ-NTer)$_2$PEt] *exo*	C_1	0.754	−2728.6454	+0.7912	0.750	−2725.9217	0.010
[$^{\bullet}$P(μ-NTer)$_2$PBr]	C_1	0.754	−5223.3180	+0.7278	0.750	−5219.6007	0.010
[$^{\bullet}$P(μ-NTer)$_2$P$^{\bullet}$]	D_2		−2649.5088	+0.7310		−2646.8678	0.010
Br$^{\bullet}$	O_h	0.752	−2573.7206	−0.0168	0.750	−2572.6609	0.009
Br_2	$D_{\infty h}$		−5147.5340	−0.0234		−5145.3954	0.006
Butane (Et_2)	C_{2h}		−158.2803	+0.1007		−158.1274	0.008
EtBr	C_S		−2652.9130	+0.0366		−2651.7686	0.007
Et$^{\bullet}$	C_S	0.754	−79.0673	+0.0334	0.750	−78.9900	0.014
[BrP(μ-NTer)$_2$PBr] *trans*	C_i		−7797.1514	+0.7310		−7792.3671	0.010
[BrP(μ-NTer)$_2$PBr] *cis*	C_1		−7797.1449	+0.7311		−7792.3610	0.010
[EtP(μ-NTer)$_2$PEt] *trans*	C_i		−2807.8075	+0.8596		−2805.0102	0.010
[EtP(μ-NTer)$_2$PEt] *cis*	C_1		−2807.8008	+0.8582		−2805.0036	0.010
[BrP(μ-NTer)$_2$PEt] *cis endo*	C_1		−5302.4760	+0.7937		−5298.6844	0.010
[BrP(μ-NTer)$_2$PEt] *cis exo*	C_1		−5302.4771	+0.7949		−5298.6862	0.010
[BrP(μ-NTer)$_2$PEt] *trans exo*	C_1		−5302.4811	+0.7932		−5298.6896	0.010
[BrP(μ-NTer)$_2$PEt] *trans endo*	C_1		−5302.4850	+0.7936		−5298.6941	0.010

[a] thermal correction to Gibbs energy in a.u. (298 K, 1 atm).

5.4 Calculated spectra

Table S8: Calculated ^{31}P NMR chemical shifts (PBE-D3/def2-TZVP), relative to PH_3.

Compound	calculated ^{31}P chemical shift	
	P1	P2
[P(μ-NTer)$_2$P]Et$_2$ (*trans*, *endo*)	289.1	289.1
[P(μ-NTer)$_2$P]Et$_2$ (*cis*, *exo*)	267.7	267.7
[P(μ-NTer)$_2$P]EtBr (*trans*, *endo*) (**2a**)	286.6 (P-Br)	256.3 (P-Et)
[P(μ-NTer)$_2$P]EtBr (cis, exo)	290.2 (P-Br)	270.7 (P-Et)
[P(μ-NTer)$_2$P]Br$_2$ (*cis*)	271.2	271.2
[P(μ-NTer)$_2$P]Br$_2$ (*trans*) (**4**)	329.0	329.0

EPR spectra of two possible intermediates during the addition of EtBr to [$^{\bullet}$P(μ-NTer)$_2$P$^{\bullet}$] were calculated. The spectra were simulated using EasySpin[70] and the online EPR simulation tool at https://www.eprsimulator.org/.[71]

Figure S21: Computed EPR spectrum (RI-SOMF(1X)/PBE0-D3/def2-TZVP) of [P(μ-NTer)$_2$PBr]$^{\bullet}$.

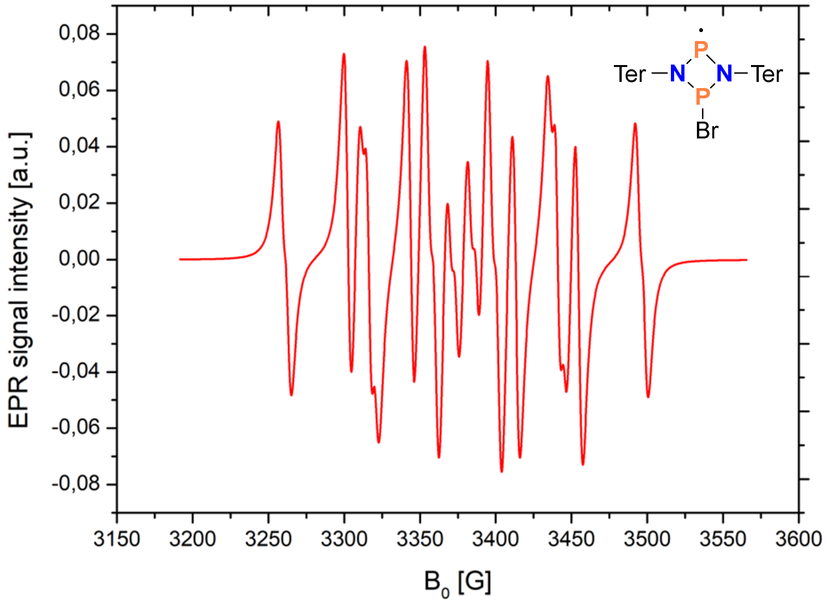

Figure S22: Computed EPR spectrum (RI-SOMF(1X)/PBE0-D3/def2-TZVP) of [P(μ-NTer)$_2$PEt]$^{\bullet}$.

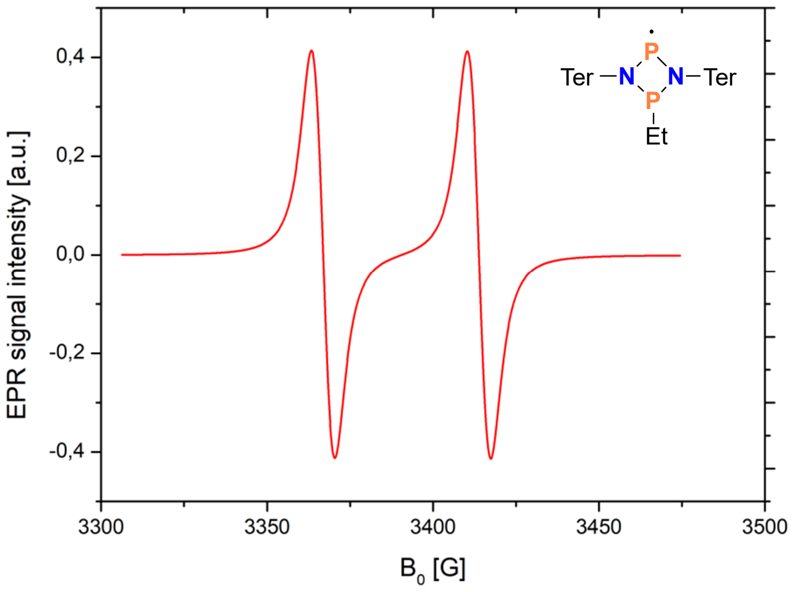

Table S9. Summary of calculated EPR data.

Compound	g_{iso}	A_{iso} [MHz]				
		N	N	P$^{\bullet}$	P(-R)	Br
[P(μ-NTer)$_2$PBr]$^{\bullet}$	2.009	0.6	1.9	148.0	161.9	113.6
[P(μ-NTer)$_2$PEt]$^{\bullet}$	2.002	4.0	4.6	131.7	−2.9	—

Figure S23: Experimental and calculated (PBE0-D3/def2-TZVP) UV-vis spectrum of [P(μ-NTer)$_2$PEt]$^{\bullet}$.

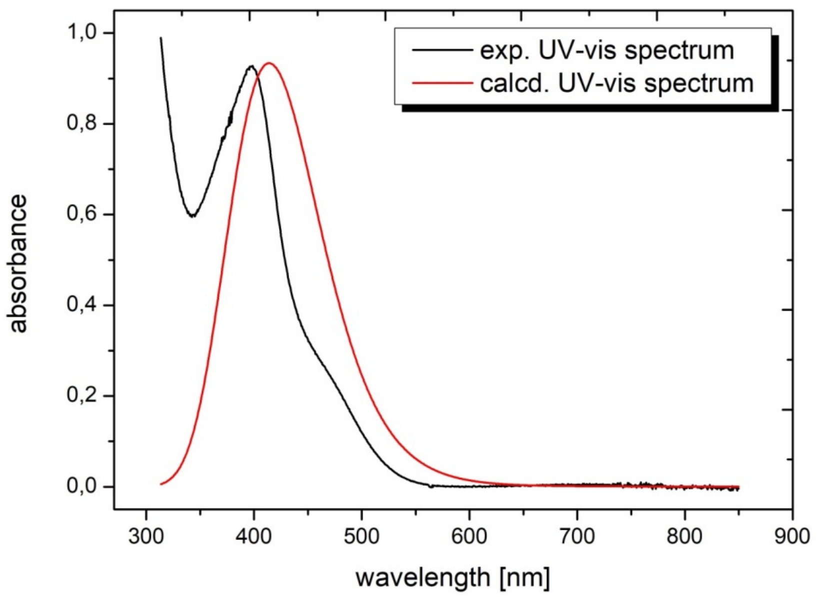

Figure S24: Natural transition orbitals (NTOs) of state #6 (414 nm), which is the main contribution to the UV-vis absorption band shown in Figure S23.

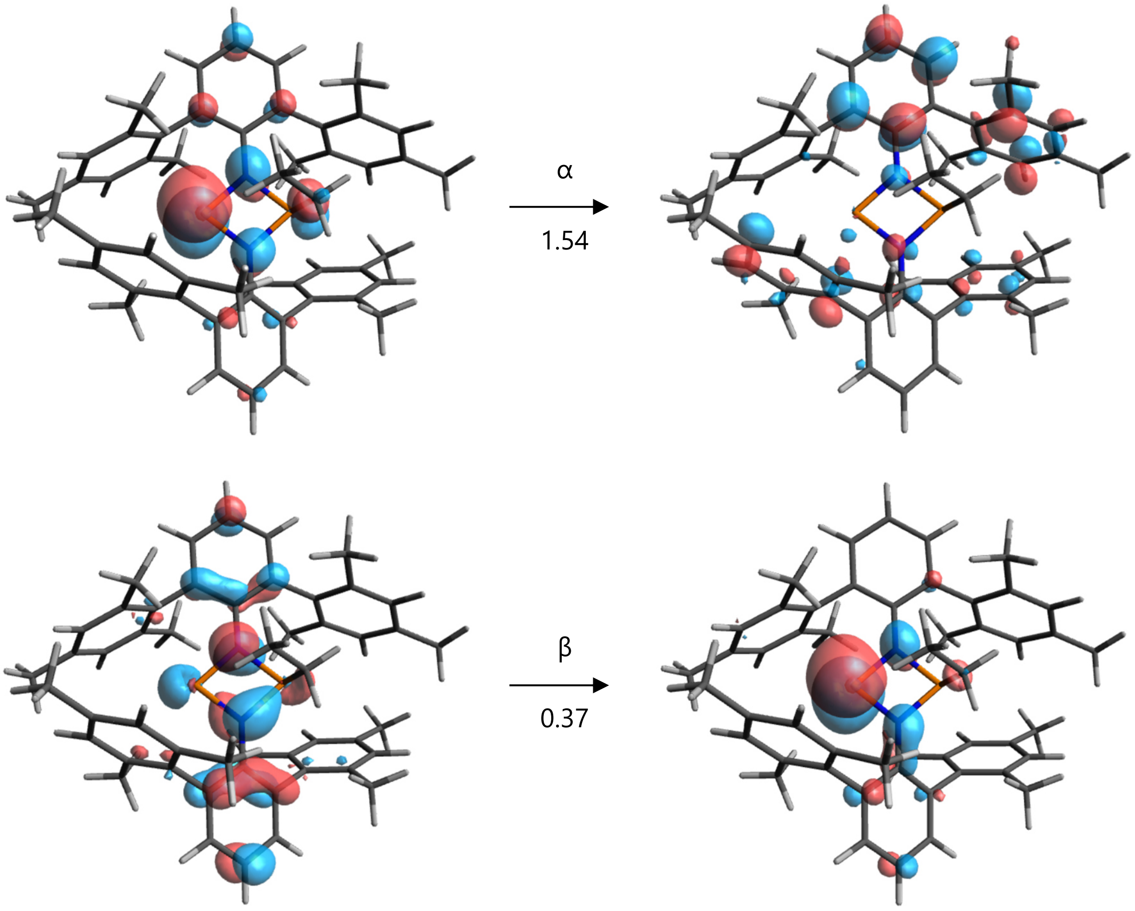

5.5 Optimized structures (.xyz-files)

5.5.1 [$^{\bullet}$P(μ-NTer)$_2$P$^{\bullet}$] (1)

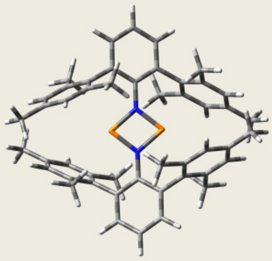

```
102
[•P(μ-NTer)2P•] @ PBE-PBE/def2-TZVP
N          -0.00000          0.00000         -1.11373
N          -0.00000          0.00000          1.11373
P           1.34195         -0.00000          0.00000
P          -1.34195          0.00000          0.00000
C           0.00000         -0.00000          2.51579
C           1.15163          0.41324          3.22973
C          -1.15163         -0.41324          3.22973
C           1.13077          0.40397          4.62874
C          -1.13077         -0.40397          4.62874
C           0.00000         -0.00000          5.33560
H           2.02606          0.73377          5.15966
H          -2.02606         -0.73377          5.15966
H           0.00000         -0.00000          6.42632
C           0.00000         -0.00000         -2.51579
C           1.15163         -0.41324         -3.22973
C          -1.15163          0.41324         -3.22973
C           1.13077         -0.40397         -4.62874
C          -1.13077          0.40397         -4.62874
C           0.00000         -0.00000         -5.33560
H           2.02606         -0.73377         -5.15966
H          -2.02606          0.73377         -5.15966
H           0.00000         -0.00000         -6.42632
C          -2.38047          0.88753         -2.53313
C          -3.49608          0.03544         -2.39727
C          -2.43121          2.20977         -2.03338
C          -4.64223          0.52090         -1.75802
C          -3.59631          2.65199         -1.40490
C          -4.71164          1.82010         -1.25104
H          -5.50022         -0.14530         -1.63830
H          -3.62928          3.67224         -1.01242
C           2.38047         -0.88753         -2.53313
C           3.49608         -0.03544         -2.39727
C           2.43121         -2.20977         -2.03338
C           4.64223         -0.52090         -1.75802
C           3.59631         -2.65199         -1.40490
C           4.71164         -1.82010         -1.25104
H           5.50022          0.14530         -1.63830
H           3.62928         -3.67224         -1.01242
C          -2.38047         -0.88753          2.53313
C          -3.49608         -0.03544          2.39727
C          -2.43121         -2.20977          2.03338
C          -4.64223         -0.52090          1.75802
C          -3.59631         -2.65199          1.40490
C          -4.71164         -1.82010          1.25104
H          -5.50022          0.14530          1.63830
H          -3.62928         -3.67224          1.01242
C           2.38047          0.88753          2.53313
C           2.43121          2.20977          2.03338
C           3.49608          0.03544          2.39727
C           3.59631          2.65199          1.40490
C           4.64223          0.52090          1.75802
```

```
C           4.71164         1.82010         1.25104
H           3.62928         3.67224         1.01242
H           5.50022        -0.14530         1.63830
C          -3.44308         1.39457         2.86433
H          -4.42258         1.87762         2.75186
H          -3.13262         1.47615         3.91534
H          -2.71204         1.96531         2.26893
C          -1.23308        -3.11288         2.13905
H          -0.84812        -3.15778         3.16829
H          -1.47900        -4.13121         1.81134
H          -0.40954        -2.74129         1.50731
C          -5.94791        -2.31721         0.55159
H          -5.71638        -2.65276        -0.47111
H          -6.38857        -3.17686         1.07962
H          -6.71366        -1.53261         0.48677
C           3.44308        -1.39457         2.86433
H           4.42258        -1.87762         2.75186
H           3.13262        -1.47615         3.91534
H           2.71204        -1.96531         2.26893
C           1.23308         3.11288         2.13905
H           0.40954         2.74129         1.50731
H           0.84812         3.15778         3.16829
H           1.47900         4.13121         1.81134
C           5.94791         2.31721         0.55159
H           5.71638         2.65276        -0.47111
H           6.38857         3.17686         1.07962
H           6.71366         1.53261         0.48677
C           5.94791        -2.31721        -0.55159
H           5.71638        -2.65276         0.47111
H           6.38857        -3.17686        -1.07962
H           6.71366        -1.53261        -0.48677
C          -5.94791         2.31721        -0.55159
H          -5.71638         2.65276         0.47111
H          -6.38857         3.17686        -1.07962
H          -6.71366         1.53261        -0.48677
C          -1.23308         3.11288        -2.13905
H          -0.84812         3.15778        -3.16829
H          -1.47900         4.13121        -1.81134
H          -0.40954         2.74129        -1.50731
C           1.23308        -3.11288        -2.13905
H           0.84812        -3.15778        -3.16829
H           1.47900        -4.13121        -1.81134
H           0.40954        -2.74129        -1.50731
C           3.44308         1.39457        -2.86433
H           4.42258         1.87762        -2.75186
H           3.13262         1.47615        -3.91534
H           2.71204         1.96531        -2.26893
C          -3.44308        -1.39457        -2.86433
H          -4.42258        -1.87762        -2.75186
H          -3.13262        -1.47615        -3.91534
H          -2.71204        -1.96531        -2.26893
```

5.5.2 [BrP(μ-NTer)$_2$PEt] *trans endo* (2a)

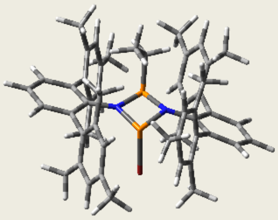

```
110
[BrP(μ-NTer)₂PEt] @ PBE-PBE/def2-TZVP
C                  -3.30136200    2.21570700    1.09711700
 C                  -2.43124800    2.71721700    0.10755700
 C                  -4.58939400    1.81620300    0.72692700
 C                  -2.85535300    2.04696300    2.52278400
 C                  -2.87448200    2.83165300   -1.23384500
 C                  -1.09098200    3.27809400    0.44966100
 C                  -5.03360800    1.88416900   -0.59493700
 H                  -5.26296200    1.43457200    1.49727500
 H                  -3.70042500    2.15437500    3.21653600
 H                  -2.07611700    2.76839500    2.80010700
 H                  -2.43987400    1.03628600    2.67179700
 C                  -4.15835500    2.39283500   -1.56044100
 C                  -1.99744100    3.44620100   -2.29032600
 C                   0.11100200    2.52332600    0.38578800
 C                  -1.03528900    4.64029300    0.76030600
 C                  -6.41618000    1.43376800   -0.98120200
 H                  -4.49026600    2.46881400   -2.59937700
 H                  -1.04159700    2.91221000   -2.38217400
 H                  -1.76096700    4.49393700   -2.04925000
 H                  -2.49242300    3.41611700   -3.26913900
 C                   1.35269000    3.20470300    0.51245000
 N                   0.07212800    1.13185400    0.22551200
 C                   0.17515100    5.29219600    0.98331100
 H                  -1.97762500    5.18938600    0.81169600
 H                  -6.37457300    0.53792200   -1.62041400
 H                  -6.94495600    2.21125800   -1.55251900
 H                  -7.02013300    1.18939400   -0.09753100
 C                   1.35333400    4.57095300    0.82950600
 C                   2.68323900    2.59665400    0.21086400
 P                  -1.21153600    0.06623000   -0.20989700
 P                   1.38071700   -0.06979400    0.34107900
 H                   0.19981400    6.35396400    1.23046600
 H                   2.31867000    5.07220100    0.91939000
 C                   3.01246400    2.28945400   -1.13614600
 C                   3.68315600    2.51720700    1.20560300
 Br                 -0.99036000   -0.05829700   -2.63699600
 N                  -0.04138200   -1.14268500    0.22928000
 C                   1.67773600   -0.11737200    2.18210200
 C                   4.31595400    1.89968000   -1.44492200
 C                   1.99985900    2.41718000   -2.23662700
 C                   4.97332400    2.10390500    0.84910500
 C                   3.41524400    2.90276800    2.63879500
 C                  -0.10510900   -2.49628100   -0.16097300
 H                   1.90240600   -1.17622300    2.37905200
 H                   2.62055100    0.42158800    2.34615400
 C                   0.56412000    0.39257100    3.08695800
 H                   4.56215700    1.68053000   -2.48756800
 C                   5.31646600    1.80919900   -0.47153900
 H                   2.48580800    2.36755100   -3.21952200
 H                   1.44798400    3.36566700   -2.16483900
 H                   1.25189000    1.60911500   -2.19851000
 H                   5.73924600    2.04017900    1.62755800
 H                   2.36043500    2.77956300    2.91254200
 H                   3.66665800    3.96053400    2.81695700
 H                   4.03217500    2.30527700    3.32494900
```

C	-1.37090900	-3.14322000	-0.21543400
C	1.05798000	-3.24370500	-0.47172500
H	-0.39612300	-0.08529100	2.85020800
H	0.42335000	1.47651100	2.98417400
H	0.79182400	0.17790000	4.14162600
C	6.72864100	1.46494800	-0.85846000
C	-1.45403800	-4.44883900	-0.71240000
C	-2.61577000	-2.54106300	0.34589000
C	0.92350400	-4.55088300	-0.95903800
C	2.45132300	-2.77621200	-0.20180500
H	7.35760800	1.28270500	0.02257200
H	7.18546600	2.28742400	-1.43116500
H	6.76575000	0.57040100	-1.49688100
H	-2.43687000	-4.92171200	-0.75007400
C	-0.32183600	-5.15077000	-1.11533700
C	-2.71961600	-2.38171600	1.75139200
C	-3.73553400	-2.27146300	-0.47026400
H	1.83538000	-5.09949300	-1.20401400
C	3.02704300	-3.10203700	1.04791000
C	3.24011000	-2.17115600	-1.20475800
H	-0.40562600	-6.16542600	-1.50612800
C	-3.91203000	-1.90822800	2.29908400
C	-1.59340800	-2.78103900	2.66568500
C	-4.91574700	-1.81083100	0.12778800
C	-3.71021800	-2.48630100	-1.96028200
C	4.36013900	-2.76278900	1.29221800
C	2.23704600	-3.83715700	2.09870600
C	4.57481500	-1.86571600	-0.92226200
C	2.64681700	-1.83680300	-2.54157100
H	-3.98262000	-1.79153300	3.38442400
C	-5.02803400	-1.61969700	1.50426100
H	-1.31109100	-3.83262100	2.50453100
H	-1.88259900	-2.65727100	3.71744100
H	-0.69184500	-2.17977400	2.48499200
H	-5.77591400	-1.59577400	-0.51146900
H	-2.70575600	-2.35604600	-2.37904700
H	-4.38405900	-1.77851700	-2.46210600
H	-4.04947200	-3.50308300	-2.21685500
H	4.79875600	-3.01045400	2.26300500
C	5.15224300	-2.14297800	0.32079900
H	1.26658500	-3.36071000	2.29884000
H	2.79798300	-3.89436600	3.04081100
H	2.00675900	-4.86336300	1.77290900
H	5.17918300	-1.39169300	-1.69981400
H	3.40504500	-1.41955200	-3.21675500
H	1.83494900	-1.09831100	-2.43702800
H	2.19797800	-2.72088500	-3.01780100
C	-6.31861300	-1.16617000	2.13116300
C	6.58264900	-1.78734400	0.62142700
H	-7.01440200	-0.77306000	1.37820700
H	-6.14796900	-0.38383400	2.88575600
H	-6.82190000	-2.00112400	2.64433500
H	7.05842100	-2.54981000	1.25433600
H	6.64109000	-0.82989200	1.16286400
H	7.17495100	-1.68275300	-0.29712800

5.5.3 [BrP(*μ*-NTer)$_2$PEt] *trans exo*

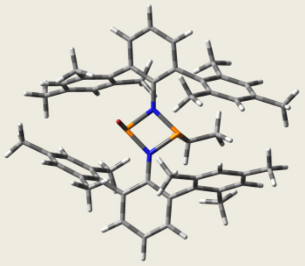

```
110
[BrP(μ-NTer)₂PEt] @ PBE-PBE/def2-TZVP
C          -3.22384         2.17534         1.20852
C          -2.46995         2.73927         0.15862
C          -4.51715         1.71968         0.94184
C          -2.63494         2.03656         2.58575
C          -3.02171         2.84577        -1.13915
C          -1.12877         3.33468         0.41659
C          -5.07876         1.79975        -0.33591
H          -5.09932         1.28156         1.75513
H          -3.36607         1.60950         3.28411
H          -2.29358         3.00480         2.98108
H          -1.75476         1.37325         2.57257
C          -4.31109         2.35759        -1.36294
C          -2.25000         3.48875        -2.25945
C           0.07266         2.58293         0.34589
C          -1.07605         4.70512         0.68640
C          -6.47917         1.32138        -0.60760
H          -4.73135         2.42676        -2.36998
H          -1.26888         3.01289        -2.39771
H          -2.06574         4.55469        -2.05547
H          -2.79934         3.40582        -3.20567
C           1.31439         3.26622         0.46412
N           0.02970         1.19082         0.18221
C           0.13521         5.36532         0.87832
H          -2.01981         5.25165         0.73987
H          -6.49213         0.56512        -1.40694
H          -7.12263         2.15093        -0.93931
H          -6.93283         0.87851         0.28785
C           1.31370         4.64009         0.74733
C           2.64514         2.64253         0.20279
P          -1.23697         0.12234        -0.29382
P           1.32237        -0.00669         0.44436
H           0.16007         6.43374         1.09504
H           2.27906         5.14240         0.83126
C           2.99422         2.29571        -1.12959
C           3.62383         2.57305         1.21923
Br         -0.97642         0.01028        -2.71574
N          -0.08197        -1.08144         0.19822
C           1.36561         0.15267         2.30760
C           4.29305         1.86780        -1.40126
C           2.00535         2.42330        -2.25164
C           4.90956         2.11534         0.89995
C           3.35377         3.02481         2.63202
C          -0.14729        -2.43334        -0.19677
H           0.42475        -0.25816         2.70290
H           1.31913         1.24105         2.46584
C           2.59478        -0.42523         3.00137
H           4.55590         1.61788        -2.43284
C           5.27068         1.77557        -0.40395
H           2.50513         2.32262        -3.22360
H           1.48824         3.39364        -2.22120
H           1.22508         1.64773        -2.20187
H           5.65917         2.05725         1.69462
H           2.28317         3.05381         2.86775
H           3.73920         4.04407         2.79497
H           3.85905         2.36935         3.35509
```

C	-1.41805	-3.07145	-0.24637
C	1.00746	-3.18158	-0.53174
H	3.51694	-0.13603	2.47764
H	2.56735	-1.51972	3.03528
H	2.65941	-0.05790	4.03615
C	6.68286	1.39183	-0.75074
C	-1.51390	-4.36749	-0.76752
C	-2.66305	-2.49044	0.34046
C	0.85877	-4.47844	-1.04299
C	2.41100	-2.74455	-0.26428
H	7.26898	1.15053	0.14559
H	7.19295	2.21919	-1.26933
H	6.71197	0.52374	-1.42430
H	-2.49921	-4.83532	-0.79940
C	-0.39141	-5.06761	-1.19795
C	-2.75872	-2.35158	1.74896
C	-3.80339	-2.25856	-0.45908
H	1.76483	-5.02756	-1.30758
C	3.00510	-3.16506	0.94748
C	3.19478	-2.10570	-1.24912
H	-0.48583	-6.07388	-1.60761
C	-3.97358	-1.96701	2.31730
C	-1.59863	-2.68001	2.64839
C	-5.00440	-1.88815	0.15947
C	-3.77719	-2.41981	-1.95628
C	4.35930	-2.90170	1.16877
C	2.20974	-3.92374	1.97793
C	4.54742	-1.87006	-0.98778
C	2.58277	-1.67489	-2.54894
H	-4.03857	-1.87850	3.40572
C	-5.11696	-1.74255	1.54149
H	-1.21209	-3.68978	2.44357
H	-1.89719	-2.63005	3.70350
H	-0.76130	-1.98456	2.49445
H	-5.88296	-1.71545	-0.46748
H	-2.78623	-2.21073	-2.37635
H	-4.49895	-1.73799	-2.42676
H	-4.05295	-3.44504	-2.25159
H	4.81343	-3.22626	2.10934
C	5.14926	-2.25433	0.21478
H	1.27769	-3.40393	2.24295
H	2.79765	-4.07299	2.89331
H	1.90935	-4.91270	1.59906
H	5.14956	-1.37515	-1.75400
H	3.34148	-1.26016	-3.22521
H	1.81062	-0.90544	-2.38719
H	2.07696	-2.51022	-3.05514
C	-6.42597	-1.38321	2.19134
C	6.60368	-1.98378	0.48932
H	-7.23366	-1.30844	1.45136
H	-6.36420	-0.42048	2.72328
H	-6.71896	-2.13869	2.93627
H	7.06820	-2.81811	1.03394
H	6.72503	-1.08198	1.11024
H	7.16713	-1.82228	-0.43916

5.5.4 [BrP(μ-NTer)$_2$PEt] *cis endo*

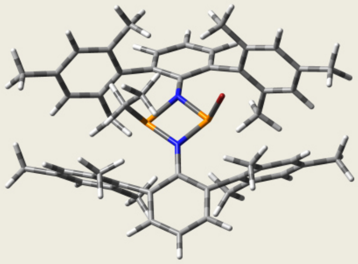

```
110
[BrP(μ-NTer)₂PEt] @ PBE-PBE/def2-TZVP
C         -3.52327      -2.67648      -0.57196
C         -2.50377      -2.58331       0.40250
C         -4.81032      -2.23308      -0.25359
C         -3.26348      -3.27623      -1.92759
C         -2.81218      -2.08912       1.69331
C         -1.17467      -3.22611       0.17008
C         -5.13387      -1.74406       1.01464
H         -5.58785      -2.28693      -1.02091
H         -3.96681      -2.87360      -2.66827
H         -3.39088      -4.37076      -1.90485
H         -2.24319      -3.07318      -2.27427
C         -4.11768      -1.67848       1.97198
C         -1.76476      -2.02380       2.76905
C          0.07046      -2.53880       0.06961
C         -1.19541      -4.62649       0.16794
C         -6.54509      -1.34602       1.35192
H         -4.34882      -1.30600       2.97342
H         -1.00699      -1.25487       2.54283
H         -1.22511      -2.97696       2.86556
H         -2.21359      -1.77255       3.73855
C          1.26501      -3.31567       0.11704
N          0.11633      -1.14122      -0.05928
C         -0.03187      -5.38543       0.11909
H         -2.16775      -5.11665       0.24271
H         -6.58396      -0.73453       2.26378
H         -7.17289      -2.23488       1.52465
H         -7.00893      -0.77592       0.53496
C          1.18467      -4.71498       0.12400
C          2.64561      -2.76611       0.25938
P         -1.17755       0.02041      -0.04383
P          1.48616      -0.00520      -0.01397
H         -0.07224      -6.47499       0.12480
H          2.12067      -5.27533       0.16416
C          3.07085      -2.24973       1.50461
C          3.58324      -2.92522      -0.78803
Br        -2.02385      -0.04943      -2.25100
N          0.14510       1.16359      -0.11328
C          2.21679      -0.04880      -1.71396
C          4.40564      -1.85709       1.66005
C          2.12394      -2.14944       2.66710
C          4.90418      -2.51589      -0.58948
C          3.17551      -3.50815      -2.11650
C          0.13852       2.41828       0.54386
H          2.78477       0.89209      -1.76122
H          2.98355      -0.83152      -1.63426
C          1.35833      -0.23622      -2.95545
H          4.72477      -1.45412       2.62473
C          5.33687      -1.97467       0.62736
H          2.66498      -1.92052       3.59416
H          1.56106      -3.08352       2.80612
H          1.38260      -1.35051       2.50509
H          5.61714      -2.62049      -1.41244
H          2.18634      -3.14895      -2.43133
H          3.10734      -4.60590      -2.07019
H          3.90697      -3.25221      -2.89467
```

C	-1.08162	3.06380	0.88057
C	1.35752	3.07294	0.87067
H	0.65822	0.59607	-3.09603
H	0.75617	-1.15278	-2.90425
H	1.99908	-0.29894	-3.84849
C	6.76904	-1.55402	0.81522
C	-1.05488	4.24455	1.63421
C	-2.42929	2.63125	0.40337
C	1.33068	4.25037	1.63054
C	2.70049	2.67801	0.34802
H	7.07233	-0.82109	0.05296
H	7.45118	-2.41358	0.72557
H	6.92682	-1.10169	1.80343
H	-2.01166	4.71195	1.87578
C	0.13735	4.83269	2.04252
C	-2.83298	2.97867	-0.90508
C	-3.36365	2.06616	1.30201
H	2.28591	4.72412	1.86634
C	3.05878	3.09916	-0.95360
C	3.66705	2.06882	1.17922
H	0.13626	5.75099	2.63054
C	-4.14682	2.71479	-1.30153
C	-1.87101	3.61524	-1.86850
C	-4.66366	1.81623	0.86112
C	-2.95530	1.70405	2.70209
C	4.35625	2.85785	-1.41525
C	2.05588	3.77813	-1.84574
C	4.94888	1.83407	0.67130
C	3.32416	1.65476	2.58151
H	-4.44986	2.97342	-2.31956
C	-5.07409	2.12415	-0.44098
H	-1.33064	4.45479	-1.40808
H	-2.39570	3.97913	-2.76123
H	-1.11734	2.88366	-2.20060
H	-5.37713	1.36479	1.55418
H	-2.13292	0.97019	2.68576
H	-3.79485	1.26254	3.25373
H	-2.58791	2.57536	3.26310
H	4.62543	3.18256	-2.42427
C	5.31318	2.21217	-0.62431
H	1.23084	3.09657	-2.10548
H	2.52541	4.11501	-2.77887
H	1.59702	4.64519	-1.34873
H	5.68557	1.34274	1.31177
H	4.20042	1.23652	3.09319
H	2.53301	0.88815	2.56471
H	2.94099	2.49800	3.17346
C	-6.46422	1.79932	-0.91481
C	6.68761	1.92091	-1.16323
H	-7.18476	1.79784	-0.08524
H	-6.48979	0.79954	-1.37879
H	-6.80903	2.51735	-1.67193
H	7.02971	2.71480	-1.84200
H	6.69187	0.97989	-1.73756
H	7.42375	1.81738	-0.35442

5.5.5 [BrP(*μ*-NTer)$_2$PEt] *cis exo*

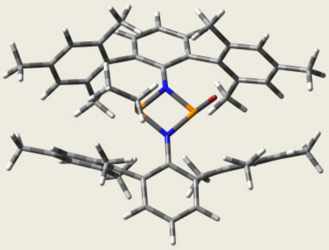

```
110
[BrP(μ-NTer)₂PEt] @ PBE-PBE/def2-TZVP
C          -3.61817        -2.64199        -0.44504
C          -2.57624        -2.56166         0.50866
C          -4.88327        -2.14969        -0.11216
C          -3.40898        -3.27441        -1.79486
C          -2.84554        -2.03254         1.79464
C          -1.26178        -3.21909         0.24287
C          -5.16732        -1.62403         1.15104
H          -5.67630        -2.19365        -0.86412
H          -4.11063        -2.85544        -2.52801
H          -3.58169        -4.36202        -1.75049
H          -2.38902        -3.11887        -2.16540
C          -4.13261        -1.57512         2.08861
C          -1.77861        -1.96797         2.85130
C          -0.01956        -2.53216         0.12072
C          -1.28126        -4.61849         0.20342
C          -6.55779        -1.16975         1.50318
H          -4.33250        -1.17534         3.08628
H          -1.04653        -1.17304         2.62951
H          -1.21275        -2.90807         2.91482
H          -2.21500        -1.75103         3.83473
C           1.17793        -3.30294         0.08375
N           0.02892        -1.13429         0.06836
C          -0.11849        -5.37147         0.07917
H          -2.24843        -5.11474         0.30072
H          -6.55915        -0.53655         2.40097
H          -7.21254        -2.03209         1.70741
H          -7.01639        -0.60313         0.68063
C           1.09872        -4.70162         0.04448
C           2.55691        -2.73974         0.19208
P          -1.24579         0.03664        -0.00072
P           1.41378        -0.03737        -0.18420
H          -0.15820        -6.46078         0.05226
H           2.03463        -5.26270         0.01557
C           3.00759        -2.24707         1.44438
C           3.47612        -2.87937        -0.87056
Br         -1.93678        -0.15304        -2.27209
N           0.08783         1.15218        -0.10544
C           1.61825        -0.27508        -2.03260
C           4.34467        -1.87900         1.59223
C           2.07158        -2.15071         2.61533
C           4.80584        -2.48141        -0.67984
C           3.07892        -3.46996        -2.19833
C           0.11553         2.43062         0.48956
H           0.80488         0.26510        -2.53448
H           1.40183        -1.34295        -2.18073
C           2.98627         0.07373        -2.61228
H           4.68118        -1.50269         2.56198
C           5.26404        -1.98838         0.54255
H           2.61483        -1.88166         3.53002
H           1.54382        -3.10084         2.78408
H           1.29664        -1.38691         2.44441
H           5.50667        -2.57487        -1.51450
H           1.99899        -3.40358        -2.37824
H           3.34207        -4.53880        -2.24619
H           3.60803        -2.96845        -3.02065
```

C	-1.09615	3.12401	0.75826
C	1.34131	3.06237	0.82349
H	3.80514	-0.27822	-1.96952
H	3.10976	1.15386	-2.73978
H	3.10547	-0.39468	-3.60060
C	6.71239	-1.63668	0.74486
C	-1.06047	4.34312	1.44541
C	-2.43667	2.68459	0.26885
C	1.32382	4.28393	1.51235
C	2.69912	2.59304	0.41119
H	7.21098	-1.41793	-0.20891
H	7.25493	-2.47122	1.21749
H	6.82712	-0.76278	1.40077
H	-2.01067	4.84691	1.63428
C	0.13693	4.92307	1.85171
C	-2.79407	2.95037	-1.07176
C	-3.40323	2.18010	1.16983
H	2.28535	4.73895	1.75941
C	3.18749	2.98733	-0.85437
C	3.57009	1.98613	1.34315
H	0.14635	5.87328	2.38655
C	-4.09534	2.66486	-1.49535
C	-1.79582	3.52563	-2.03721
C	-4.68952	1.90981	0.70254
C	-3.04065	1.89026	2.59915
C	4.52797	2.74608	-1.17055
C	2.28687	3.67599	-1.84470
C	4.89833	1.75500	0.98086
C	3.06579	1.57017	2.69442
H	-4.36238	2.85743	-2.53776
C	-5.05391	2.13383	-0.63033
H	-1.28437	4.40325	-1.61588
H	-2.28374	3.81706	-2.97613
H	-1.02101	2.78181	-2.28127
H	-5.42827	1.50591	1.39836
H	-2.25087	1.12174	2.64593
H	-3.90956	1.51726	3.15608
H	-2.64740	2.77756	3.11520
H	4.90239	3.05713	-2.15005
C	5.39943	2.12486	-0.27193
H	1.43162	3.04100	-2.12343
H	2.83448	3.93265	-2.76091
H	1.86064	4.59874	-1.42342
H	5.56503	1.27894	1.70444
H	3.86980	1.13195	3.29975
H	2.26859	0.81726	2.58487
H	2.63028	2.41518	3.24696
C	-6.42866	1.77867	-1.12703
C	6.82848	1.84982	-0.65444
H	-7.18400	1.88069	-0.33521
H	-6.45445	0.73329	-1.47651
H	-6.72696	2.41224	-1.97374
H	7.25044	2.67375	-1.24735
H	6.90165	0.93765	-1.26839
H	7.46175	1.70201	0.23067

5.5.6 [$^{\bullet}$P(μ-NTer)$_2$PEt] *endo* (3Et$^{\bullet}$)

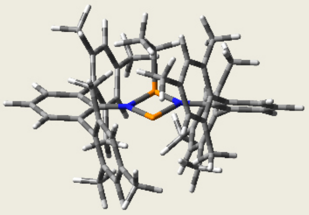

```
109
[P(μ-NTer)]2Et  @ PBE-PBE/def2-TZVP
C                  -3.30136200    2.21570700    1.09711700
 C                  -2.43124800    2.71721700    0.10755700
 C                  -4.58939400    1.81620300    0.72692700
 C                  -2.85535300    2.04696300    2.52278400
 C                  -2.87448200    2.83165300   -1.23384500
 C                  -1.09098200    3.27809400    0.44966100
 C                  -5.03360800    1.88416900   -0.59493700
 H                  -5.26296200    1.43457200    1.49727500
 H                  -3.70042500    2.15437500    3.21653600
 H                  -2.07611700    2.76839500    2.80010700
 H                  -2.43987400    1.03628600    2.67179700
 C                  -4.15835500    2.39283500   -1.56044100
 C                  -1.99744100    3.44620100   -2.29032600
 C                   0.11100200    2.52332600    0.38578800
 C                  -1.03528900    4.64029300    0.76030600
 C                  -6.41618000    1.43376800   -0.98120200
 H                  -4.49026600    2.46881400   -2.59937700
 H                  -1.04159700    2.91221000   -2.38217400
 H                  -1.76096700    4.49393700   -2.04925000
 H                  -2.49242300    3.41611700   -3.26913900
 C                   1.35269000    3.20470300    0.51245000
 N                   0.07212800    1.13185400    0.22551200
 C                   0.17515100    5.29219600    0.98331100
 H                  -1.97762500    5.18938600    0.81169600
 H                  -6.37457300    0.53792200   -1.62041400
 H                  -6.94495600    2.21125800   -1.55251900
 H                  -7.02013300    1.18939400   -0.09753100
 C                   1.35333400    4.57095300    0.82950600
 C                   2.68323900    2.59665400    0.21086400
 P                  -1.21153600    0.06623000   -0.20989700
 P                   1.38071700   -0.06979400    0.34107900
 H                   0.19981400    6.35396400    1.23046600
 H                   2.31867000    5.07220100    0.91939000
 C                   3.01246400    2.28945400   -1.13614600
 C                   3.68315600    2.51720700    1.20560300
 Br                 -0.99036000   -0.05829700   -2.63699600
 N                  -0.04138200   -1.14268500    0.22928000
 C                   1.67773600   -0.11737200    2.18210200
 C                   4.31595400    1.89968000   -1.44492200
 C                   1.99985900    2.41718000   -2.23662700
 C                   4.97332400    2.10390500    0.84910500
 C                   3.41524400    2.90276800    2.63879500
 C                  -0.10510900   -2.49628100   -0.16097300
 H                   1.90240600   -1.17622300    2.37905200
 H                   2.62055100    0.42158800    2.34615400
 C                   0.56412000    0.39257100    3.08695800
 H                   4.56215700    1.68053000   -2.48756800
 C                   5.31646600    1.80919900   -0.47153900
 H                   2.48580800    2.36755100   -3.21952200
 H                   1.44798400    3.36566700   -2.16483900
 H                   1.25189000    1.60911500   -2.19851000
 H                   5.73924600    2.04017900    1.62755800
 H                   2.36043500    2.77956300    2.91254200
 H                   3.66665800    3.96053400    2.81695700
 H                   4.03217500    2.30527700    3.32494900
```

C	-1.37090900	-3.14322000	-0.21543400
C	1.05798000	-3.24370500	-0.47172500
H	-0.39612300	-0.08529100	2.85020800
H	0.42335000	1.47651100	2.98417400
H	0.79182400	0.17790000	4.14162600
C	6.72864100	1.46494800	-0.85846000
C	-1.45403800	-4.44883900	-0.71240000
C	-2.61577000	-2.54106300	0.34589000
C	0.92350400	-4.55088300	-0.95903800
C	2.45132300	-2.77621200	-0.20180500
H	7.35760800	1.28270500	0.02257200
H	7.18546600	2.28742400	-1.43116500
H	6.76575000	0.57040100	-1.49688100
H	-2.43687000	-4.92171200	-0.75007400
C	-0.32183600	-5.15077000	-1.11533700
C	-2.71961600	-2.38171600	1.75139200
C	-3.73553400	-2.27146300	-0.47026400
H	1.83538000	-5.09949300	-1.20401400
C	3.02704300	-3.10203700	1.04791000
C	3.24011000	-2.17115600	-1.20475800
H	-0.40562600	-6.16542600	-1.50612800
C	-3.91203000	-1.90822800	2.29908400
C	-1.59340800	-2.78103900	2.66568500
C	-4.91574700	-1.81083100	0.12778800
C	-3.71021800	-2.48630100	-1.96028200
C	4.36013900	-2.76278900	1.29221800
C	2.23704600	-3.83715700	2.09870600
C	4.57481500	-1.86571600	-0.92226200
C	2.64681700	-1.83680300	-2.54157100
H	-3.98262000	-1.79153300	3.38442400
C	-5.02803400	-1.61969700	1.50426100
H	-1.31109100	-3.83262100	2.50453100
H	-1.88259900	-2.65727100	3.71744100
H	-0.69184500	-2.17977400	2.48499200
H	-5.77591400	-1.59577400	-0.51146900
H	-2.70575600	-2.35604600	-2.37904700
H	-4.38405900	-1.77851700	-2.46210600
H	-4.04947200	-3.50308300	-2.21685500
H	4.79875600	-3.01045400	2.26300500
C	5.15224300	-2.14297800	0.32079900
H	1.26658500	-3.36071000	2.29884000
H	2.79798300	-3.89436600	3.04081100
H	2.00675900	-4.86336300	1.77290900
H	5.17918300	-1.39169300	-1.69981400
H	3.40504500	-1.41955200	-3.21675500
H	1.83494900	-1.09831100	-2.43702800
H	2.19797800	-2.72088500	-3.01780100
C	-6.31861300	-1.16617000	2.13116300
C	6.58264900	-1.78734400	0.62142700
H	-7.01440200	-0.77306000	1.37820700
H	-6.14796900	-0.38383400	2.88575600
H	-6.82190000	-2.00112400	2.64433500
H	7.05842100	-2.54981000	1.25433600
H	6.64109000	-0.82989200	1.16286400
H	7.17495100	-1.68275300	-0.29712800

5.5.7 [$^{\bullet}$P(μ-NTer)$_2$PEt] *exo*

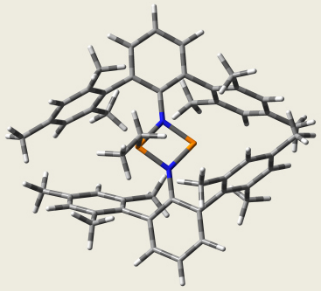

```
109
[P(μ-NTer)]₂Et (exo)  @ PBE-PBE/def2-TZVP
N          0.02416        1.14290        0.01057
N          0.13820       -1.13810        0.16873
P         -1.17471       -0.03435        0.57170
P          1.36625        0.03401       -0.26871
C          0.17294       -2.52949        0.04566
C         -0.96098       -3.26688       -0.38864
C          1.37765       -3.22635        0.33842
C         -0.88747       -4.66491       -0.44188
C          1.40990       -4.62115        0.24567
C          0.28195       -5.35145       -0.12438
H         -1.76925       -5.21153       -0.78205
H          2.34503       -5.13201        0.48497
H          0.31968       -6.43977       -0.18363
C         -0.00050        2.53215        0.04179
C         -1.17710        3.25962        0.38751
C          1.17524        3.26437       -0.28902
C         -1.11687        4.65504        0.48426
C          1.18295        4.65712       -0.18025
C          0.05222        5.36534        0.22378
H         -2.03279        5.18644        0.75054
H          2.10263        5.18577       -0.44020
H          0.07262        6.45270        0.30069
C          2.39444        2.59556       -0.82705
C          3.53259        2.38999       -0.01699
C          2.42740        2.23332       -2.19629
C          4.67726        1.82493       -0.58800
C          3.58929        1.66196       -2.72036
C          4.72478        1.44681       -1.93228
H          5.55334        1.65911        0.04348
H          3.60696        1.37831       -3.77639
C         -2.50485        2.61249        0.59003
C         -3.21419        2.10660       -0.52781
C         -3.11592        2.59885        1.86450
C         -4.48327        1.55947       -0.33971
C         -4.37218        1.99913        2.01258
C         -5.06951        1.46633        0.92598
H         -5.02174        1.17520       -1.20767
H         -4.82357        1.96104        3.00827
C          2.61494       -2.51134        0.76757
C          3.63809       -2.23090       -0.16180
C          2.78300       -2.16389        2.12676
C          4.79965       -1.58793        0.28320
C          3.94642       -1.50156        2.52206
C          4.96862       -1.20166        1.61330
H          5.58887       -1.36904       -0.44012
H          4.06489       -1.22414        3.57356
C         -2.20864       -2.62360       -0.88903
C         -2.19034       -1.93562       -2.12491
C         -3.42593       -2.78513       -0.19571
C         -3.38835       -1.43141       -2.63763
C         -4.60018       -2.25692       -0.74431
C         -4.60694       -1.58702       -1.96883
H         -3.36926       -0.90832       -3.59800
H         -5.53938       -2.38069       -0.19706
C          3.47984       -2.56086       -1.62213
```

H	4.44375	-2.49366	-2.14402
H	3.06439	-3.56579	-1.77603
H	2.78714	-1.84859	-2.10196
C	1.73828	-2.53414	3.14364
H	0.73219	-2.24235	2.81583
H	1.70985	-3.62444	3.29793
H	1.94217	-2.05766	4.11165
C	6.21009	-0.48098	2.06523
H	5.97300	0.53882	2.40748
H	6.69095	-0.99860	2.90894
H	6.94478	-0.40169	1.25268
C	-3.49675	-3.51723	1.11966
H	-4.29331	-3.10185	1.75259
H	-3.71850	-4.58644	0.97232
H	-2.54891	-3.46460	1.66927
C	-0.91433	-1.74815	-2.90047
H	-0.26541	-0.98461	-2.43910
H	-0.32428	-2.67528	-2.93593
H	-1.12782	-1.42926	-3.92918
C	-5.89059	-1.08132	-2.57093
H	-5.72182	-0.19061	-3.19310
H	-6.34977	-1.84556	-3.21869
H	-6.62684	-0.82703	-1.79554
C	-6.40175	0.79226	1.10851
H	-6.28610	-0.30357	1.08885
H	-6.86356	1.06192	2.06791
H	-7.10032	1.05787	0.30191
C	5.95747	0.81435	-2.51943
H	5.75752	-0.22217	-2.83400
H	6.30231	1.35896	-3.41127
H	6.78163	0.79371	-1.79382
C	1.22737	2.45017	-3.07715
H	0.86414	3.48645	-3.01499
H	1.46462	2.22185	-4.12425
H	0.39099	1.80292	-2.77006
C	-2.46393	3.23006	3.06825
H	-2.80900	4.26848	3.19897
H	-2.72399	2.68409	3.98620
H	-1.37111	3.27500	2.97594
C	-2.61551	2.13934	-1.90535
H	-3.38671	1.97826	-2.66948
H	-2.10577	3.09206	-2.10529
H	-1.86590	1.33939	-2.02038
C	3.49992	2.68433	1.45728
H	4.51544	2.72292	1.87351
H	2.98847	3.62935	1.68186
H	2.95059	1.88444	1.98276
C	-0.86516	0.12173	2.40990
C	-1.74480	-0.82701	3.21950
H	0.20946	-0.04458	2.57914
H	-1.08424	1.16447	2.67177
H	-2.81046	-0.63700	3.02331
H	-1.57005	-0.70337	4.29833
H	-1.54476	-1.88017	2.97341

5.5.8 [BrP(μ-NTer)$_2$PBr] *trans* (4)

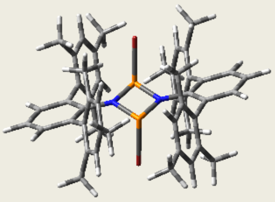

```
104
[BrP(μ-NTer)₂PBr] @ PBE-PBE/def2-TZVP
C                -3.08882200    2.24184200    1.27955000
 C                -2.32478200    2.79948100    0.23175300
 C                -4.42189300    1.90469400    1.03564900
 C                -2.46745900    1.99043500    2.62508500
 C                -2.90653600    3.01641000   -1.03876200
 C                -0.94152400    3.30065000    0.47060200
 C                -5.01545400    2.09673000   -0.21699300
 H                -5.01268400    1.47740400    1.84912600
 H                -3.20013800    1.56610700    3.32346500
 H                -2.05936200    2.91402200    3.06204900
 H                -1.62249300    1.28575700    2.55142600
 C                -4.23801200    2.64533900   -1.24063500
 C                -2.12635400    3.66424500   -2.15045000
 C                 0.21266700    2.50684100    0.27088600
 C                -0.79879300    4.64492300    0.83172800
 C                -6.44931900    1.72175000   -0.47237900
 H                -4.68618500    2.80840400   -2.22441800
 H                -1.19184600    3.12392200   -2.35699900
 H                -1.84683300    4.69693300   -1.89141600
 H                -2.71565100    3.68579300   -3.07589200
 C                 1.49232600    3.11697100    0.32858200
 N                 0.09962700    1.12648000    0.01697200
 C                 0.45618600    5.22839000    0.98111000
 H                -1.70597400    5.23208200    0.98759200
 H                -6.51401500    0.72019100   -0.92576500
 H                -6.92701800    2.42673600   -1.16712500
 H                -7.03373700    1.70188900    0.45678600
 C                 1.58478700    4.46392400    0.70535000
 C                 2.75707500    2.45332300   -0.10403800
 P                -1.28479400    0.12407300   -0.33346200
 P                 1.28475000   -0.12413500    0.33333300
 H                 0.55206500    6.27485000    1.27271400
 H                 2.57739500    4.91533000    0.74630500
 C                 2.95053300    2.18147900   -1.48462300
 C                 3.83104800    2.28663000    0.79766800
 Br               -1.28439100    0.07309500   -2.69001200
 N                -0.09986800   -1.12655600   -0.01677700
 C                 4.20100700    1.74555300   -1.92126500
 C                 1.85810800    2.40986600   -2.49167300
 C                 5.06693600    1.84250800    0.30875800
 C                 3.70872300    2.61665800    2.26234300
 C                -0.21274800   -2.50684100   -0.27108700
 H                 4.34680600    1.56068600   -2.98937500
 C                 5.28094000    1.58474400   -1.04483600
 H                 2.25695600    2.35921800   -3.51305300
 H                 1.38391100    3.39229300   -2.35078400
 H                 1.05850000    1.65784000   -2.41176000
 H                 5.89554100    1.72526900    1.01310500
 H                 2.67066400    2.57816200    2.61010500
 H                 4.09161500    3.62918200    2.46958300
 H                 4.29950600    1.91335300    2.86517100
 C                -1.49240600   -3.11694800   -0.32901400
 C                 0.94143400   -3.30059600   -0.47090400
 C                 6.63811300    1.20243300   -1.56961000
 C                -1.58491800   -4.46376100   -0.70624300
```

C	-2.75708100	-2.45333300	0.10388500
C	0.79867900	-4.64474200	-0.83254000
C	2.32469000	-2.79959100	-0.23167900
H	7.35711600	1.04605100	-0.75534000
H	7.03825900	1.99153800	-2.22518100
H	6.59657000	0.28070000	-2.16927200
H	-2.57754300	-4.91511100	-0.74738100
C	-0.45629700	-5.22813300	-0.98224100
C	-2.95038600	-2.18170500	1.48456000
C	-3.83110800	-2.28631300	-0.79768200
H	1.70586400	-5.23185600	-0.98855500
C	2.90616500	-3.01681600	1.03892700
C	3.08898000	-2.24180800	-1.27919600
H	-0.55215200	-6.27449200	-1.27421400
C	-4.20077100	-1.74575300	1.92139100
C	-1.85785300	-2.41008200	2.49150400
C	-5.06693700	-1.84222800	-0.30855400
C	-3.70897900	-2.61566900	-2.26253300
C	4.23760400	-2.64584000	1.24115700
C	2.12567500	-3.66480000	2.15031000
C	4.42201900	-1.90475900	-1.03494300
C	2.46786700	-1.98994100	-2.62475900
H	-4.34647700	-1.56108200	2.98954900
C	-5.28079100	-1.58472900	1.04509800
H	-1.38194600	-3.39143400	2.34909000
H	-2.25704800	-2.36181000	3.51286700
H	-1.05949300	-1.65655700	2.41302700
H	-5.89558700	-1.72475600	-1.01281300
H	-2.67068500	-2.57975600	-2.60981700
H	-4.29752700	-1.91029100	-2.86513300
H	-4.09463900	-3.62697200	-2.47063100
H	4.68558700	-2.80916000	2.22498500
C	5.01528600	-2.09702700	0.21779400
H	1.19200600	-3.12339400	2.35787900
H	2.71537700	-3.68805800	3.07545600
H	1.84463400	-4.69682600	1.89034000
H	5.01298800	-1.47738900	-1.84824200
H	3.20095700	-1.56644400	-3.32320800
H	1.62364300	-1.28435400	-2.55112100
H	2.05874900	-2.91312600	-3.06160600
C	-6.63788900	-1.20250400	1.57013200
C	6.44906900	-1.72202200	0.47359700
H	-7.35690500	-1.04554900	0.75598300
H	-6.59616700	-0.28113100	2.17033300
H	-7.03812400	-1.99193800	2.22525500
H	6.92633200	-2.42647100	1.16918000
H	6.51364600	-0.72008400	0.92616600
H	7.03396300	-1.70299200	-0.45529200
Br	1.28470800	-0.07310900	2.68980500

5.5.9 [BrP(μ-NTer)$_2$PBr] *cis*

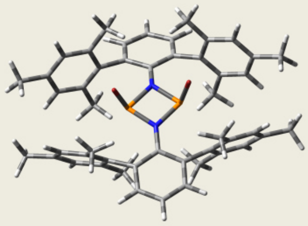

```
104
[BrP(μ-NTer)₂PBr] @ PBE-PBE/def2-TZVP
C           3.55963        2.69196       -0.64273
C           2.58531        2.62567        0.37800
C           4.86599        2.27495       -0.36328
C           3.23551        3.23599       -2.00789
C           2.95452        2.17491        1.67098
C           1.23691        3.23610        0.17468
C           5.24995        1.83680        0.90547
H           5.61067        2.31367       -1.16322
H           3.36941        4.32956       -2.03710
H           2.19848        3.02612       -2.29560
H           3.90058        2.79941       -2.76464
C           4.27392        1.78640        1.90663
C           1.95626        2.13399        2.79379
C           0.01088        2.51687        0.15654
C           1.21192        4.63494        0.10204
C           6.68172        1.48832        1.20774
H           4.55072        1.44828        2.90900
H           1.20303        1.34502        2.63260
H           1.40439        3.08115        2.87815
H           2.45239        1.92836        3.75086
C          -1.20954        3.24509        0.19790
N           0.00494        1.11214        0.13771
C           0.01982        5.34836        0.04939
H           2.16780        5.16136        0.11656
H           6.75485        0.64956        1.91438
H           7.20081        2.34426        1.66845
H           7.23329        1.22020        0.29716
C          -1.17620        4.64363        0.12453
C          -2.55686        2.64192        0.42722
P           1.32681       -0.03047        0.07190
P          -1.32952       -0.01650        0.07460
H           0.02332        6.43741       -0.00536
H          -2.12781        5.17690        0.15742
C          -2.90009        2.18415        1.72416
C          -3.55240        2.71486       -0.57363
Br          1.88496        0.02078       -2.18079
N          -0.00776       -1.16384        0.09272
C          -4.21453        1.79133        1.98308
C          -1.88014        2.13252        2.82678
C          -4.85109        2.29182       -0.27201
C          -3.25684        3.27003       -1.94084
C          -0.01727       -2.47365        0.62088
H          -4.47123        1.44582        2.98815
C          -5.20872        1.84232        1.00126
H          -2.36051        1.94255        3.79508
H          -1.30823        3.06895        2.89339
H          -1.14600        1.32741        2.65596
H          -5.61089        2.33290       -1.05757
H          -2.22600        3.06319       -2.25193
H          -3.39183        4.36371       -1.95817
H          -3.93751        2.83937       -2.68701
C           1.19830       -3.14628        0.90511
C          -1.24340       -3.13592        0.88372
C          -6.63284        1.48085        1.32403
C           1.16154       -4.39012        1.54813
```

C	2.54207	-2.66194	0.47068
C	-1.22829	-4.37993	1.52734
C	-2.57520	-2.64129	0.42428
H	-7.14508	1.04306	0.45689
H	-7.20308	2.37506	1.62351
H	-6.68980	0.76594	2.15656
H	2.11366	-4.88202	1.75724
C	-0.03908	-5.00516	1.88849
C	2.97428	-2.96365	-0.84108
C	3.43875	-2.07705	1.39332
H	-2.18800	-4.86395	1.71942
C	-2.98075	-2.93220	-0.89837
C	-3.48924	-2.05909	1.33184
H	-0.04759	-5.97316	2.39050
C	4.28000	-2.63572	-1.21415
C	2.05134	-3.62713	-1.82516
C	4.73438	-1.76793	0.97585
C	2.99260	-1.74963	2.79020
C	-4.27727	-2.59709	-1.29665
C	-2.03870	-3.58924	-1.86849
C	-4.77444	-1.74154	0.88911
C	-3.07173	-1.74133	2.73974
H	4.60688	-2.85946	-2.23303
C	5.17300	-2.03017	-0.32657
H	1.59262	-4.53255	-1.40179
H	2.59017	-3.90002	-2.74148
H	1.23002	-2.95140	-2.10960
H	5.42064	-1.30562	1.68905
H	2.19032	-0.99252	2.77015
H	3.82205	-1.34476	3.38409
H	2.58355	-2.62833	3.30895
H	-4.58327	-2.81250	-2.32377
C	-5.18609	-1.99338	-0.42425
H	-1.21608	-2.90841	-2.13716
H	-2.56073	-3.86175	-2.79459
H	-1.58281	-4.49368	-1.44013
H	-5.47412	-1.28089	1.59009
H	-3.91736	-1.35654	3.32401
H	-2.28240	-0.97028	2.74046
H	-2.65665	-2.61890	3.25528
C	6.55694	-1.64847	-0.77499
C	-6.55827	-1.60072	-0.89880
H	7.25109	-1.57379	0.07286
H	6.54167	-0.66837	-1.27871
H	6.96137	-2.37643	-1.49219
H	-6.93820	-2.30274	-1.65412
H	-6.53350	-0.60235	-1.36548
H	-7.27734	-1.56088	-0.06929
Br	-1.89058	0.06629	-2.17604

5.5.10 [$^{\bullet}$P(μ-NTer)$_2$PBr] (3Br$^{\bullet}$)

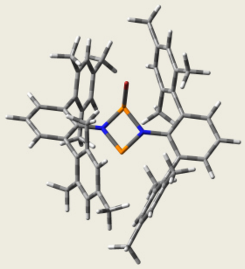

```
103
[•P(μ-NTer)₂PBr] @ PBE-PBE/def2-TZVP
N          0.06534       -1.13358        0.06065
N          0.18769        1.12173        0.07580
P         -1.16708        0.06501       -0.20254
P          1.46611       -0.06442        0.20968
C          0.23908        2.52122        0.08400
C         -0.90070        3.27349        0.46432
C          1.43589        3.19575       -0.26151
C         -0.84000        4.67136        0.42623
C          1.45438        4.59495       -0.27153
C          0.32339        5.34014        0.05419
H         -1.72544        5.23336        0.72907
H          2.38519        5.09462       -0.54746
H          0.35437        6.43018        0.03726
C          0.00582       -2.53409        0.04287
C         -1.20223       -3.22714       -0.22821
C          1.17798       -3.28390        0.31970
C         -1.18759       -4.62587       -0.29277
C          1.14518       -4.67987        0.24069
C         -0.02550       -5.36180       -0.07973
H         -2.13033       -5.13481       -0.50217
H          2.06391       -5.22783        0.45964
H         -0.03855       -6.45076       -0.13538
C          2.44418       -2.64756        0.78100
C          3.53697       -2.49031       -0.09790
C          2.56960       -2.28245        2.14312
C          4.73744       -1.97650        0.40311
C          3.78486       -1.76546        2.59653
C          4.88331       -1.61047        1.74364
H          5.58057       -1.84802       -0.27931
H          3.87710       -1.47950        3.64800
C         -2.52257       -2.55610       -0.39838
C         -3.24113       -2.12322        0.74108
C         -3.12210       -2.49307       -1.67636
C         -4.52587       -1.60357        0.57449
C         -4.39957       -1.93934       -1.79717
C         -5.11635       -1.48438       -0.68724
H         -5.07827       -1.27586        1.45723
H         -4.84712       -1.86302       -2.79166
C          2.69576        2.47461       -0.60493
C          3.66114        2.23666        0.39704
C          2.96155        2.11716       -1.94715
C          4.87206        1.63348        0.04029
C          4.17723        1.50119       -2.25254
C          5.14792        1.25466       -1.27514
H          5.61788        1.44996        0.81716
H          4.37649        1.21820       -3.28995
C         -2.14296        2.63974        0.98903
C         -2.13550        2.06114        2.28010
C         -3.34353        2.69687        0.24735
C         -3.32869        1.55529        2.80267
C         -4.51253        2.17760        0.81329
C         -4.53141        1.61371        2.09125
H         -3.31977        1.11420        3.80347
H         -5.43891        2.21819        0.23392
C          3.39214        2.59595        1.83421
```

H	4.28330	2.42518	2.45224
H	3.08552	3.64620	1.94252
H	2.57455	1.98156	2.24559
C	1.95766	2.39820	-3.02818
H	1.68379	3.46367	-3.05057
H	2.35418	2.11934	-4.01304
H	1.02469	1.83686	-2.86475
C	6.46155	0.62065	-1.64599
H	6.31031	-0.31616	-2.20357
H	7.05429	1.28585	-2.29315
H	7.06478	0.39653	-0.75614
C	-3.38015	3.28752	-1.13722
H	-4.33510	3.05946	-1.62840
H	-3.26277	4.38159	-1.11630
H	-2.56910	2.88949	-1.76377
C	-0.87239	1.98448	3.09564
H	-0.18216	1.22290	2.69692
H	-0.32606	2.93858	3.08426
H	-1.09700	1.72174	4.13754
C	-5.81511	1.12194	2.70345
H	-5.64244	0.26573	3.37124
H	-6.28917	1.91241	3.30754
H	-6.53883	0.82122	1.93362
C	-6.47491	-0.85909	-0.84831
H	-6.38588	0.23175	-0.97901
H	-6.99808	-1.25206	-1.73088
H	-7.10595	-1.03301	0.03456
C	6.18871	-1.07684	2.26818
H	6.04185	-0.14174	2.82934
H	6.66097	-1.79416	2.95762
H	6.89837	-0.88057	1.45366
C	1.40987	-2.43465	3.08912
H	1.00205	-3.45579	3.06590
H	1.71052	-2.19943	4.11812
H	0.58393	-1.75949	2.81383
C	-2.41634	-3.02312	-2.89446
H	-2.46535	-4.12303	-2.93376
H	-2.87640	-2.62985	-3.80992
H	-1.35391	-2.74678	-2.89757
C	-2.63568	-2.19271	2.11563
H	-3.40612	-2.07345	2.88843
H	-2.10602	-3.14067	2.28381
H	-1.90184	-1.38082	2.25908
C	3.40376	-2.81959	-1.55979
H	4.34712	-2.63424	-2.08954
H	3.11377	-3.86812	-1.71958
H	2.62246	-2.19971	-2.02746
Br	-1.25359	0.30755	-2.60248

5.5.11 EtBr

```
8
EtBr @ PBE-PBE/def2-TZVP
C         -0.59827      -1.08231       0.00000
H         -1.22375      -1.18027       0.89384
H         -1.22375      -1.18027      -0.89384
C          0.57303      -2.03870       0.00000
H          1.20106      -1.90190       0.89010
H          0.19680      -3.07474       0.00000
H          1.20106      -1.90190      -0.89010
Br         0.00000       0.79900       0.00000
```

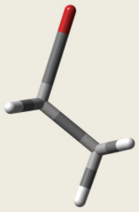

5.5.12 $Et^{\bullet}$

```
7
Ethyl radical @ PBE-PBE/def2-TZVP
C          0.00958      -0.69069       0.00000
H          0.50461      -1.10377       0.89211
H         -1.01960      -1.10672       0.00000
H          0.50461      -1.10377      -0.89211
C          0.00958       0.79200      -0.00000
H         -0.05226       1.35320      -0.93181
H         -0.05226       1.35320       0.93181
```

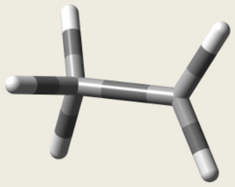

5.5.13 PH_3

```
4
PH3 @ PBE-PBE/def2-TZVP
P         -0.00000       0.00000       0.13202
H          0.00000       1.19102      -0.66012
H         -1.03145      -0.59551      -0.66012
H          1.03145      -0.59551      -0.66012
```

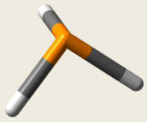

5.5.14 Br_2

```
2
Br2 @ PBE-PBE/def2-TZVP
Br        -0.00000      -0.00000       1.15569
Br         0.00000      -0.00000      -1.15569
```

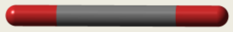

5.5.15 butane

```
14
Butane @ PBE-PBE/def2-TZVP
C         -0.70278       1.82960       0.00000
H         -0.18511       2.22540       0.88761
H         -0.18511       2.22540      -0.88761
H         -1.72460       2.23637       0.00000
C         -0.70278       0.30151       0.00000
H         -1.25456      -0.07010       0.88108
H         -1.25456      -0.07010      -0.88108
C          0.70278      -0.30151       0.00000
H          1.25456       0.07010      -0.88108
```

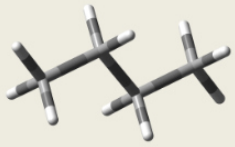

H	1.25456	0.07010	0.88108
C	0.70278	-1.82960	0.00000
H	0.18511	-2.22540	0.88761
H	0.18511	-2.22540	-0.88761
H	1.72460	-2.23637	0.00000

6 References

[1] J. Bresien, T. Kröger-Badge, S. Lochbrunner, D. Michalik, H. Müller, A. Schulz, E. Zander, *Chem. Sci.* **2019**, *10*, 3486–3493.

[2] G. R. Fulmer, A. J. M. Miller, N. H. Sherden, H. E. Gottlieb, A. Nudelman, B. M. Stoltz, J. E. Bercaw, K. I. Goldberg, *Organometallics* **2010**, *29*, 2176–2179.

[3] G. M. Sheldrick, *Acta Crystallogr. Sect. A Found. Adv.* **2015**, *71*, 3–8.

[4] G. M. Sheldrick, *Acta Crystallogr. Sect. C Struct. Chem.* **2015**, *71*, 3–8.

[5] G. M. Sheldrick, *SADABS Version 2*, University of Göttingen, Germany, **2004**.

[6] *Gaussian 09, Revision E.01*, M. J. Frisch, G. W. Trucks, H. B. Schlegel, G. E. Scuseria, M. A. Robb, J. R. Cheeseman, G. Scalmani, V. Barone, B. Mennucci, G. A. Petersson, H. Nakatsuji, M. Caricato, X. Li, H. P. Hratchian, A. F. Izmaylov, J. Bloino, G. Zheng, J. L. Sonnenberg, M. Hada, M. Ehara, K. Toyota, R. Fukuda, J. Hasegawa, M. Ishida, T. Nakajima, Y. Honda, O. Kitao, H. Nakai, T. Vreven, J. A. Montgomery Jr., J. E. Peralta, F. Ogliaro, M. Bearpark, J. J. Heyd, E. Brothers, K. N. Kudin, V. N. Staroverov, T. Keith, R. Kobayashi, J. Normand, K. Raghavachari, A. Rendell, J. C. Burant, S. S. Iyengar, J. Tomasi, M. Cossi, N. Rega, J. M. Millam, M. Klene, J. E. Knox, J. B. Cross, V. Bakken, C. Adamo, J. Jaramillo, R. Gomperts, R. E. Stratmann, O. Yazyev, A. J. Austin, R. Cammi, C. Pomelli, J. W. Ochterski, R. L. Martin, K. Morokuma, V. G. Zakrzewski, G. A. Voth, P. Salvador, J. J. Dannenberg, S. Dapprich, A. D. Daniels, O. Farkas, J. B. Foresman, J. V. Ortiz, J. Cioslowski, D. J. Fox, Gaussian, Inc., Wallingford CT, **2013**.

[7] F. Neese, *Wiley Interdiscip. Rev.-Comput. Mol. Sci.* **2018**, *8*, e1327.

[8] F. Neese, F. Wennmohs, U. Becker, C. Riplinger, *J. Chem. Phys.* **2020**, *152*, 224108.

[9] T. Lu, F. Chen, *J. Comput. Chem.* **2012**, *33*, 580–592.

[10] E. D. Glendening, J. K. Badenhoop, A. E. Reed, J. E. Carpenter, J. A. Bohmann, C. M. Morales, C. R. Landis, F. Weinhold, *NBO 6.0*, Theoretical Chemistry Institute, University of Wisconsin, Madison, **2013**.

[11] J. E. Carpenter, F. Weinhold, *J. Mol. Struct. THEOCHEM* **1988**, *169*, 41–62.

[12] F. Weinhold, J. E. Carpenter, *The Structure of Small Molecules and Ions*, Plenum Press, **1988**.

[13] F. Weinhold, C. R. Landis, *Valency and Bonding. A Natural Bond Orbital Donor-Acceptor Perspective*, Cambridge University Press, **2005**.

[14] J. P. Perdew, K. Burke, M. Ernzerhof, *Phys. Rev. Lett.* **1996**, *77*, 3865–3868.

[15] J. P. Perdew, K. Burke, M. Ernzerhof, *Phys. Rev. Lett.* **1997**, *78*, 1396–1396.

[16] S. Grimme, J. Antony, S. Ehrlich, H. Krieg, *J. Chem. Phys.* **2010**, *132*, 154104.

[17] S. Grimme, S. Ehrlich, L. Goerigk, *J. Comput. Chem.* **2011**, *32*, 1456–1465.

[18] F. Weigend, R. Ahlrichs, *Phys. Chem. Chem. Phys.* **2005**, *7*, 3297–305.

[19] F. Weigend, *Phys. Chem. Chem. Phys.* **2006**, *8*, 1057–1065.

[20] F. London, *J. Phys. le Radium* **1937**, *8*, 397–409.

[21] R. McWeeny, *Phys. Rev.* **1962**, *126*, 1028–1034.

[22] R. Ditchfield, *Mol. Phys.* **1974**, *27*, 789–807.

[23] K. Wolinski, J. F. Hinton, P. Pulay, *J. Am. Chem. Soc.* **1990**, *112*, 8251–8260.

[24] J. R. Cheeseman, G. W. Trucks, T. A. Keith, M. J. Frisch, *J. Chem. Phys.* **1996**, *104*, 5497–5509.

[25] C. J. Jameson, A. De Dios, A. Keith Jameson, *Chem. Phys. Lett.* **1990**, *167*, 575–582.

[26] C. van Wüllen, *Phys. Chem. Chem. Phys.* **2000**, *2*, 2137–2144.

[27] F. Neese, *J. Chem. Phys.* **2001**, *115*, 11080–11096.

[28] F. Neese, *J. Chem. Phys.* **2003**, *118*, 3939–3948.

[29] F. Neese, *J. Chem. Phys.* **2005**, *122*, 34107.

[30] F. Neese, *eMagRes* **2017**, 1–22.

[31] B. A. Heß, C. M. Marian, U. Wahlgren, O. Gropen, *Chem. Phys. Lett.* **1996**, *251*, 365–371.

[32] C. Adamo, V. Barone, *J. Chem. Phys.* **1999**, *110*, 6158–6170.

[33] F. Neese, F. Wennmohs, A. Hansen, U. Becker, *Chem. Phys.* **2009**, *356*, 98–109.

[34] R. Bauernschmitt, R. Ahlrichs, *Chem. Phys. Lett.* **1996**, *256*, 454–464.

[35] R. E. Stratmann, G. E. Scuseria, M. J. Frisch, *J. Chem. Phys.* **1998**, *109*, 8218–8224.

[36] M. E. Casida, C. Jamorski, K. C. Casida, D. R. Salahub, *J. Chem. Phys.* **1998**, *108*, 4439–4449.

[37] C. Riplinger, F. Neese, *J. Chem. Phys.* **2013**, *138*, 034106.

[38] D. G. Liakos, M. Sparta, M. K. Kesharwani, J. M. L. Martin, F. Neese, *J. Chem. Theory Comput.* **2015**, *11*, 1525–1539.

[39] C. Riplinger, P. Pinski, U. Becker, E. F. Valeev, F. Neese, *J. Chem. Phys.* **2016**, *144*, 024109.

[40] D. G. Liakos, Y. Guo, F. Neese, *J. Phys. Chem. A* **2020**, *124*, 90–100.

[41] A. Hellweg, C. Hättig, S. Höfener, W. Klopper, *Theor. Chem. Acc.* **2007**, *117*, 587–597.

[42] T. J. Lee, P. R. Taylor, *Int. J. Quantum Chem.* **1989**, *36*, 199–207.

[43] C. J. Cramer, *Essentials of Computational Chemistry: Theories and Models*, John Wiley & Sons, Ltd, Chichester, UK, **2004**.

[44] D. Hegarty, M. A. Robb, *Mol. Phys.* **1979**, *38*, 1795–1812.

[45] R. H. A. Eade, M. A. Robb, *Chem. Phys. Lett.* **1981**, *83*, 362–368.

[46] H. B. Schlegel, M. A. Robb, *Chem. Phys. Lett.* **1982**, *93*, 43–46.

[47] F. Bernardi, A. Bottoni, J. J. W. McDouall, M. A. Robb, H. B. Schlegel, *Faraday Symp. Chem. Soc.* **1984**, *19*, 137.

[48] P. E. M. Siegbahn, *Chem. Phys. Lett.* **1984**, *109*, 417–423.

[49] M. A. Robb, U. Niazi, in *Reports in Molecular Theory, Vol. 1* (Eds.: H. Weinstein, G. Náray-Szabó), CRC Press, Boca Raton, FL, **1990**, pp. 23–55.

[50] M. J. Frisch, I. N. Ragazos, M. A. Robb, H. B. Schlegel, *Chem. Phys. Lett.* **1992**, *189*, 524–528.

[51] N. Yamamoto, T. Vreven, M. A. Robb, M. J. Frisch, H. B. Schlegel, *Chem. Phys. Lett.* **1996**, *250*, 373–378.

[52] M. Klene, M. A. Robb, M. J. Frisch, P. Celani, *J. Chem. Phys.* **2000**, *113*, 5653–5665.

[53] C. Angeli, R. Cimiraglia, S. Evangelisti, T. Leininger, J.-P. Malrieu, *J. Chem. Phys.* **2001**, *114*, 10252–10264.

[54] C. Angeli, R. Cimiraglia, J.-P. Malrieu, *Chem. Phys. Lett.* **2001**, *350*, 297–305.

[55] C. Angeli, R. Cimiraglia, J.-P. Malrieu, *J. Chem. Phys.* **2002**, *117*, 9138–9153.

[56] G. Mills, H. Jónsson, G. K. Schenter, *Surf. Sci.* **1995**, *324*, 305–337.

[57] H. Jónsson, G. Mills, K. W. Jacobsen, in *Classical and Quantum Dynamics in Condensed Phase Simulations*, WORLD SCIENTIFIC, **1998**, pp. 385–404.

[58] G. Henkelman, B. P. Uberuaga, H. Jónsson, *J. Chem. Phys.* **2000**, *113*, 9901–9904.

[59] G. Henkelman, H. Jónsson, *J. Chem. Phys.* **2000**, *113*, 9978–9985.

[60] E. Maras, O. Trushin, A. Stukowski, T. Ala-Nissila, H. Jónsson, *Comput. Phys. Commun.* **2016**, *205*, 13–21.

[61] A. Hinz, A. Schulz, A. Villinger, *Angew. Chem., Int. Ed.* **2016**, *55*, 12214–12218.

[62] V. V. Zhivonitko, J. Bresien, A. Schulz, I. V. Koptyug, *Phys. Chem. Chem. Phys.* **2019**, *21*, 5890–5893.

[63] V. V. Zhivonitko, H. Beer, D. O. Zakharov, J. Bresien, A. Schulz, *ChemPhysChem* **2021**, *22*, 813–817.

[64] J. M. Foster, S. F. Boys, *Rev. Mod. Phys.* **1960**, *32*, 300–302.

[65] D. A. Kleier, T. A. Halgren, J. H. Hall, W. N. Lipscomb, *J. Chem. Phys.* **1974**, *61*, 3905–3919.

[66] E. Ramos-Cordoba, P. Salvador, E. Matito, *Phys. Chem. Chem. Phys.* **2016**, *18*, 24015–24023.

[67] E. Ramos-Cordoba, E. Matito, *J. Chem. Theory Comput.* **2017**, *13*, 2705–2711.

[68] M. D. Hanwell, D. E. Curtis, D. C. Lonie, T. Vandermeersch, E. Zurek, G. R. Hutchison, *J. Cheminform.* **2012**, *4*, 17.

[69] S. Grimme, A. Hansen, *Angew. Chem., Int. Ed.* **2015**, *54*, 12308–12313.

[70] S. Stoll, A. Schweiger, *J. Magn. Reson.* **2006**, *178*, 42–55.

[71] Victor Chechik, “EPR Simulator,” can be found under https://www.eprsimulator.org/, **2022**
